# Millisecond-Long Red Phosphorescence in Chiral Heteroleptic Ag(I) Complexes Bearing Benzothiazole Ligands

**DOI:** 10.1021/acs.inorgchem.6c01416

**Published:** 2026-08-06

**Authors:** Valerio Giuso, Christophe Gourlaouen, Lorenzo Arrico, Lorenzo Di Bari, Matteo Mauro

**Affiliations:** † 9308 Université de Strasbourg & CNRS, Institut de Physique Et Chimie Des Matériaux de Strasbourg UMR 7504, Strasbourg F-67034, France; ‡ Laboratoire de Modélisation Et Simulation Moléculaire, UMR7140 Chimie de la Matière Complexe, 27083 Institut Le Bel, Strasbourg F-67081, France; § Dipartimento di Chimica E Chimica Industriale, 9310 Università di Pisa, Via Moruzzi 13, Pisa 56124, Italy; ∥ Department of Chemical Sciences, University of Padova, Via Marzolo 1, Padova 35121, Italy

## Abstract

Six orange-red phosphorescent chiral Ag­(I) complexes of general structure [Ag­(N^1^^N^2^)­(P^P)]­PF_6_, where N^1^^N^2^ is a pyridyl- or quinolyl-functionalized benzo­[*d*]­thiazole and P^P is an enantiopure diphosphine of the BINAP family with axial chirality, are herein presented. The novel complexes have been extensively characterized from a chemical, structural, and photophysical point of view, resulting in a family of chiral Ag­(I) coordination compounds with red photoluminescence. Their emissive properties can be attributed to ligand-centered triplet manifold excited states localized on the benzothiazole ligand (^3^LC_btz_). Low photoluminescence quantum yield (PLQY) values are recorded in solution, as is typical of Ag­(I) derivatives, whereas a remarkable switch-on of the emission is observed in the solid state, with samples of quinolyl-benzothiazole *xyl*BINAP complexes *R*-**3** and *S*-**3** displaying PLQY as high as 50% and excited-state lifetimes in the millisecond range. Electron circular dichroism (ECD) spectra show strong activity in both solution and solid state as thin films for all the complexes. Time-dependent density functional theory (TD-DFT) calculations confirm the strong LC_btz_ nature of the major absorbing singlet and emissive triplet states and provide an argument behind the absence of circularly polarized luminescence (CPL) in these samples.

## Graphical abstract

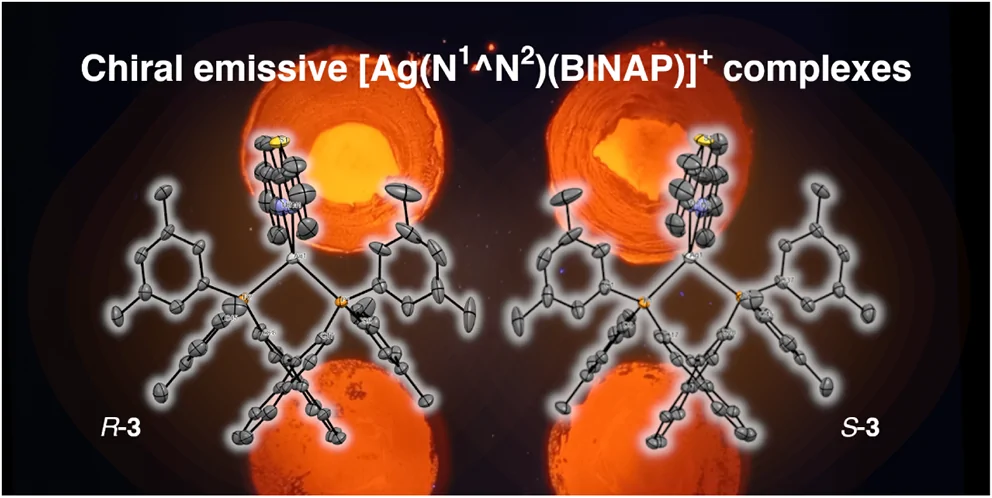

## Introduction

Photoactive transition metal complexes are attracting a great deal of attention due to their application as electroactive materials in light-emitting devices and optoelectronic technologies, and in solar energy harvesting,^1–3^ as well as the role as efficient photocatalysts in chemical transformations.^4,5^ To date, leading compounds are based on cyclometalated Ir­(III) complexes owing to the ease of modulation of both redox and optical properties by chemical design, strong spin–orbit coupling (SOC), excellent photoluminescence quantum yield, and stability that prompted real-market applications in organic light-emitting diode (OLED) technology.^2,6,7^ However, there is increasing concern about the sustainability of the long-term use of iridium due to the scarcity of this element, which is the least abundant in the Earth’s crust (<0.001 ppm), and its high price.^8^ Alternative complexes based on cheaper and more abundant first-row transition metals are attracting growing interest, although they require profound rethinking of the design strategies for the coordination environment.^4,9,10^ Among these, Cu­(I) complexes play an important role owing to their closed-shell *d* ^10^ electronic configuration that rules out the presence of low-lying quenching metal-centered states (MC), ease of synthesis, and modulation of the emissive excited state, allowing for photo- and electroluminescence in the blue- to red-region,^11^ and up to the near-infrared as recently demonstrated by us,^12,13^ as well as possibility of having a radiative process involving a thermally activated delayed fluorescence (TADF) mechanism.^14–16^ The latter enables efficient harvesting of 100% of the electrogenerated excitons in light-emitting devices. The vast majority of the most efficient photoactive copper complexes have the general structure [Cu­(N^N)­(P^P)]^+^, where N^N is a chelating α,α′-diimine of the 2,2′-bipyridine and 1,10-phenanthroline series, and P^P is a chelating, rigid, diphosphine ligand, such as Xantphos and DPEphos. Tetracoordinated Cu­(I) complexes adopt a pseudotetrahedral coordination geometry in their electronic ground state; upon photoexcitation into the metal-to-ligand charge transfer (MLCT) excited state, the formal oxidation to a *d* ^9^ Cu­(II) center promotes large structural deformations toward a square-pyramidal geometry due to pseudo Jahn–Teller distortion. This effect favors detrimental nonradiative processes from the ^3^MLCT states that may efficiently quench the emission properties of the compounds. Ag­(I) complexes have recently emerged as a trade-off alternative that shares a similar electronic configuration and coordination environment with Cu­(I) counterparts. However, compared to the latter, the former possesses *i*) a larger ionic radius and *ii*) 4d orbitals that lie at lower energy, resulting in higher oxidation potential of the metal center. Consequently, Ag­(I) complexes are characterized by ^1,3^MLCT states that are pushed up to higher energy, and therefore their lower-lying emissive excited state possesses mainly ligand-centered (^3^LC) and ligand-to-ligand charge transfer (^3^LLCT) character. Nevertheless, defining the nature of the emissive excited state in Ag­(I) complexes is sometimes not an easy task, and examples of TADF,^15–19^ phosphorescence,^20,21^ and dual-phosphorescence^22^ emission have been reported so far, partly due to the little participation of the metal center in the excited-state dynamics. In addition, the little d_π_(Ag)–p orbital interactions between the metal d orbital and the π systems of the ligand yield metal complexes that are often hypsochromically shifted compared to the Cu­(I) counterparts, making the design of efficient red-emissive Ag­(I) complexes particularly challenging.^11,23^ This is made more difficult if one considers that *i*) red to NIR emitters possess intrinsically smaller *k*_r_ due to the third-power dependency with emission energy, as described by Einstein’s theory of spontaneous emission; *ii*) the smaller spin-orbit coupling (SOC) constant associated with the silver ion compared to iridium yields complexes that further slow down *k*_r_; and *iii*) vibronic coupling between the T_1_ and ground state becomes very efficient for longer wavelength emitters due to the negative Napierian logarithmic dependency on the T_1_–S_0_ energy difference (*energy gap law*).^24^ Examples of red-emissive Ag­(I) complexes are still extremely rare in the literature. An example of note is the complex [Ag­(deebq)­(Xantphos)]­PF_6_ reported by Costa and coworkers,^22^ where deebq is 4,4′-diethylester-2,2′-biquinoline, that display λ_em_ = 655 nm, a photoluminescence quantum yield (PLQY) of ca. 42%, and τ = 2.38 μs in a doped PMMA thin film.

Ag­(I) complexes that emit in the longer-wavelength region with high efficiency and millisecond-long phosphorescence are very scarce. Design strategies would require judicious considerations in terms of both the electronic and steric properties of the coordination geometry around the metal center, also minimizing radiationless channels. A few examples of chiral Ag­(I) complexes that display some emission properties have been reported to date, but they mainly deal with coordination polymers.^25–27^

Herein, a design strategy is proposed yielding a family of mononuclear [Ag­(N^1^^N^2^)­(P^P)]­PF_6_ complexes that combines the extension of the π-conjugation in a nonsymmetric benzothiazole chromophoric ligand N^1^^N^2^ to bathochromically shift the emission wavelength, aided by a tightening of the coordination sphere through increased steric hindrance of the P^P chelate, which will also introduce a chiral element to enable chiroptical properties.

## Results and Discussion

### Synthesis and Characterization

The two N^1^^N^2^ ligands **L1–L2** were prepared following a microwave-assisted condensation already reported by our group, and their synthesis and optical properties are reported elsewhere by us.^28^ The six cationic enantiopure Ag­(I) complexes (Scheme 1) were obtained by stepwise addition of AgPF_6_ to the corresponding enantiopure chiral diphosphine (P^P) in dry CH_2_Cl_2_, followed by the addition of the corresponding bidentate N^N ligand. The crude mixture was then added dropwise to a large volume of petroleum ether, where the desired complexes flocculated and could be collected in excellent yield with purity sufficient for further chemical and photophysical characterization. Further details about the synthetic procedure and the relative ^1^H, ^13^C­{^1^H}, and ^31^P­{^1^H} NMR spectra along with the high-resolution electrospray mass spectrometry (HR-ESI-MS) characterization are reported in the Supporting Information (Figures S1–S12). The ^31^P­{^1^H} NMR spectrum of the complexes recorded in CD_2_Cl_2_ is characterized by two doublets (^1^ *J*_Ag–P_ = 200–400 Hz) of about 1:1 intensity at δ ca. 15 ppm owing to the coupling with the two NMR-active nuclei of the Ag isotopes, namely ^107^Ag and ^109^Ag, of natural abundance of 52% and 48%, respectively.^29^

**Scheme 1:**
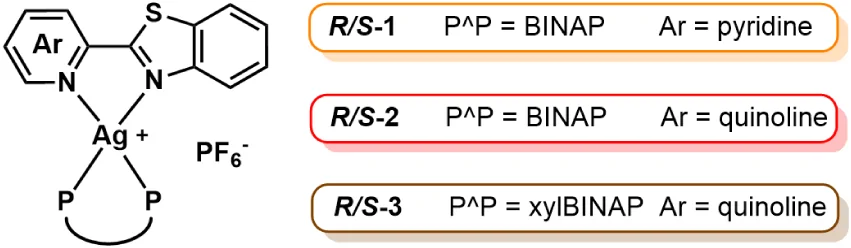
Structure of the Six Cationic Enantiopure Mononuclear Ag­(I) Complexes of the Current Work

Single crystals of complexes *S*-**2**, *R*-**3,** and *S*-**3** suitable for X-ray crystallographic analysis were obtained by vapor diffusion of Et_2_O in a CH_2_Cl_2_ solution of the corresponding cationic complex kept at −30 °C. The molecular structures of *R*-**3** and *S*-**3** are shown in Figure 1, while the structure of *S*-**2**, alongside other crystallographic data, can be found in the Supporting Information (see Figure S13 and Tables S1–S3 of the Supporting Information).

**Figure 1:**
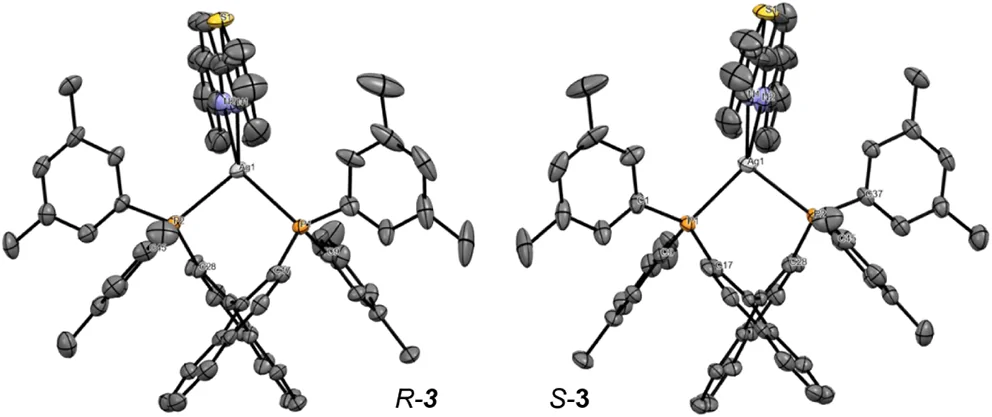
ORTEP diagrams of *R*-**3** (*left*) and *S*-**3** (*right*) shown at 50% probability. Hydrogen atoms and the PF_6_^–^ counteranion are omitted for clarity.

The crystallographic structures confirm the four-coordinated sphere around the metal center with the general formula [Ag­(N^N)­(P^P)]^+^, and the structural parameters of our complexes are comparable to those of other relevant achiral [Ag­(N^N)­(P^P)]^+^ complexes similar in size and bearing N^N = substituted 2,2′-bipyridine,^22^ phenanthroline,^30^ or benzothiazole^31^ ligands already reported in the literature, and to some benzothiazole-based [Cu­(N^N)­(P^P)]^+^ analogues.^12,13,32^ In contrast with previously reported achiral DPEphos and Xantphos complexes, the axial torsion of the BINAP, which locks the planes of the two naphthyl moieties at an acute angle (76.4° and 74.2° for *R*-**3** and *S*-**3**, respectively), allows for better intramolecular π-stacking between the naphthyl and xylyl moieties. The π-stacking distances, calculated between the plane of the flatter naphthyl and the centroid of the more distorted xylyl, are in the range of *d* = 3.37 and 3.34 Å for *R*-**3** and *S*-**3**, respectively. These values are similar in complex *S*-**2**, with the naphthyl angle being 77.6° and the π-stacking distance *d* = 3.34 Å.

The structural parameters for complexes complexes *S*-**2**, *R*-**3,** and *S*-**3** are summarized in Table 1. For *R*-**3,** the Ag–N_btz_, Ag–N_py_, Ag–P(1), and Ag–P(2) bond lengths are 2.327(4), 2.378(4), 2.4533(18), and 2.454(2) Å, respectively. Similar parameters are obtained for the structure of the enantiomer *S*-**3** as well as for complex *S*-**2**. As expected, the Ag–N and Ag–P bonds are about 0.2–0.3 Å longer than the corresponding Cu–N and Cu–P distances in related [Cu­(N^N)­(P^P)]^+^ complexes owing to the larger radius of the Ag­(I) compared to the Cu­(I) center (*r*_Cu_ = 1.54 Å vs *r*_Ag_ = 1.65 Å).^12,13,32^ The near-zero Flack parameter value obtained for all the investigated structures confirmed the enantiopurity of the prepared complexes (see Tables S1–S3 of the Supporting Information).

**Table 1:** Selected Bond Lengths [Å] and Angles [°] around the Ag­(I) Center for Complexes *S*-**2**, *R*-**3,** and *S*-**3** with Their Tetrahedral Geometric Indexes Calculated According to eqs 1–2 ^a^

	Ag–N_btz_	Ag–N_py_	Ag–P(1)	Ag–P(2)	τ_4_	$\tau_{4}^{\prime }$
*S*-**2**	2.352(3)	2.333(2)	2.4399(7)	2.4907(7)	0.70	0.66
*R*-**3**	2.327(4)	2.378(4)	2.4533(18)	2.454(2)	0.70	0.65
*S*-**3**	2.328(3)	2.386(4)	2.4456(14)	2.4654(16)	0.69	0.64

a N_btz_ = Nitrogen of the benzothiaazole moiety, N_py_ = Nitrogen of the pyridyl moiety.

In an ideal *T*_d_ environment, the angle between any two ligands is θ ≈ 109.5°. A deviation from the ideal geometry causes the ligands to be at different angles α and β from each other. This deformation can be quantified through the geometry index for four-coordinated metal centers τ_4_ proposed by Houser et al. (eq 1)^33^ or its improved version $\tau_{4}^{\prime }$ by Okuniewski et al. (eq 2).^34^

1 $$\tau_{4}=\frac{360^{{}^{\circ}}-\left(\alpha +\beta \right)}{360^{{}^{\circ}}-2\cdot \theta }\approx -0.00709\alpha -0.00709\beta +2.55$$

2 $$\tau_{4}^{\prime }=\frac{\beta -\alpha }{360^{{}^{\circ}}-\theta }+\frac{180^{{}^{\circ}}-\beta }{180^{{}^{\circ}}-\theta }\approx -0.00399\alpha -0.01019\beta +2.55$$

where τ_4_ = $\tau_{4}^{\prime }$ = 1 for an ideal tetrahedral geometry, τ_4_ ≈ 0.43 and $\tau_{4}^{\prime }$ ≈ 0.24 for a seesaw geometry where and α = 120° and β = 180°, and τ_4_ = $\tau_{4}^{\prime }$ = 0 for an ideal square planar geometry. The *τ*_4_ and $\tau_{4}^{\prime }$ values are 0.70, 0.65 and 0.69, 0.64 for *R*-**3** and *S*-**3**, respectively, indicating a slight distortion of the Ag­(I) center from the coordination geometry with ideal *T*_d_ symmetry. These values are in accordance with those of previously reported complexes: τ_4_ and $\tau_{4}^{\prime }$ are 0.74, 0.75 and 0.83, 0.84 for [Ag­(dmp)­(DPEphos)]^+^ ^31^ and [Ag­(deebq)­(Xantphos)]^+^ ^25^ respectively, where dmp is 2,9-dimethyl-1,10-phenanthroline. For complex *S*-**2**, τ_4_ and $\tau_{4}^{\prime }$ are 0.70 and 0.66, respectively, suggesting a negligible effect of the methyl groups on the geometry distortion. No intermolecular π–π interaction is observed in the crystal structure, and the supramolecular organization of the complexes seems to be driven by C–H···F short contacts between the aromatic moieties of both the N^N and P^P ligands and the PF_6_^–^ counterion. Moreover, Small and Wide-Angle X-ray Scattering (SWAXS) diffractograms (Figures S14) recorded for the as-obtained powders appear to be amorphous for the BINAP derivatives, whereas samples of complexes *R*-**3** and *S*-**3** are semicrystalline.

The thermal properties of the six complexes were assessed by thermogravimetric analysis (TGA) under a nitrogen atmosphere. The pairs of *R/S* enantiomers display virtually identical TGA profiles (Figures S22–27), with 5% weight loss decomposition temperatures (T_5%_) slowly increasing down the series **1** (≈255 °C) → **2** (≈260 °C) → **3** (≈270 °C).

### Photophysical Investigation

For the sake of clarity, only the photophysical data related to the silver complexes of *R*-BINAP (*R*-**1**, *R*-**2**) and *R*-*xyl*BINAP (*R-* **3**) will be reported hereafter and discussed in detail, unless a specific comparison between the *R*- and *S*-enantiomers is needed. All photophysical data related to the complexes of *S*-BINAP (*S*-**1**, *S*-**2**) and *S-xyl*BINAP (*S-* **3**) can be found in its entirety in the Supporting Information. The photophysical properties of ligands **L1** and **L2** along with related derivatives are reported elsewhere by us.^25^

Owing to the labile nature of the complexes, CH_2_Cl_2_ is the only solvent employed for measurements in solution at low temperatures and for the preparation of spin-coated thin films. Absorption and emission spectra collected in dilute CH_2_Cl_2_ are displayed in Figure 2, and the corresponding data can be found in Table 2 (see Figure S15 and Table S4 of the Supporting Information for the *S*-series). It is important to note that CH_2_Cl_2_ solutions of complexes **1** and **3** are photolabile upon UV irradiation, while complex **2** is virtually nonemissive in both air-equilibrated and degassed CH_2_Cl_2_ solution, with photoluminescence quantum yields (PLQYs) <0.1%. For these reasons, no detailed photophysical investigation of the solution in dilute CH_2_Cl_2_ will be presented.

**Figure 2:**
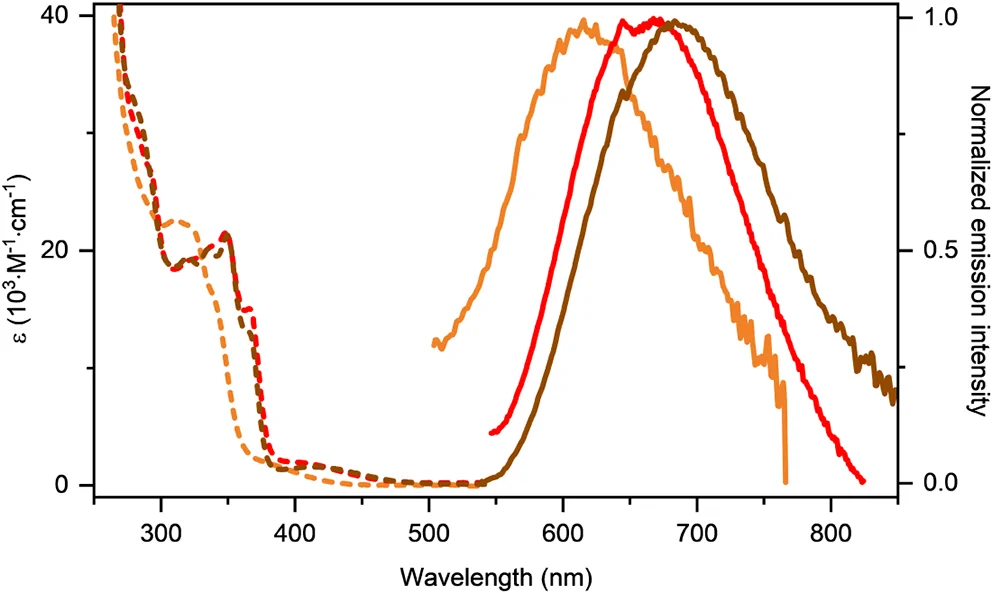
Electronic absorption (dashed) and emission spectra (solid traces) of complexes R-**1** (orange), *R*-**2** (red), *R*-**3** (brown) in dilute (2 × 10^–5^ M) degassed CH_2_Cl_2_ at room temperature upon excitation at λ_exc_ = 350, 360, and 370 nm, respectively.

**Table 2:** Photophysical Data Recorded for the *R*-Enantiomers in Dilute Degassed CH_2_Cl_2_ at Room Temperature^a^

	λ_max_, Abs(ε) [nm (10^3^ M^–1^ cm^–1^)]	λ_em_ [nm]
*R*-**1**	278*sh* (28.13), 293*sh* (23.38), 311 (22.68), 325*sh* (21.74), 341 (15.55), 379*sh* (1.99)	610 (*dec.*)
*R*-**2**	283*sh* (29.91), 293*sh* (26.42), 321*sh* (19.09), 337*sh* (20.20), 349 (21.32), 366 (14.42) 405*sh* (1.64)	670
*R*-**3**	283*sh* (32.81), 293*sh* (19.40), 335*sh* (20.10), 349 (21.45), 366 (13.76) 414*sh* (1.78)	685 (*dec.*)

a *sh* denotes the shoulder; *dec*. indicates decomposition of the sample.

In their electronic absorption spectrum, all complexes show a couple of intense shoulders at higher energies (λ_abs_ = 270–290 nm, ε = 1.8–3.2 × 10^4^ M^–1^ cm^–1^) and a set of less intense bands at lower energies (λ_abs_ = 310–370 nm, ε = 1.3–2.1 × 10^4^ M^–1^ cm^–1^). The nature, shape, and energies observed for these bands can be easily explained by comparison between the electronic absorption spectra of the ligands and the corresponding complexes.^28^ The bands at higher energies, which are absent in the free **L1**–**L2** ligands, can be attributed to singlet ligand-centered (^1^LC) π–π* transitions mostly involving the flexible aryls of the chelating diphosphine. The bands at lower energy, practically identical in shape between the **L1**–**L2** ligands and their corresponding complexes, are attributable to π–π* ^1^LC transitions mostly centered on the chromophoric N^N scaffolds. This assignment explains the bathochromic shift of the λ_abs,max_ (ca. 3400 cm^–1^) and the vibronic progression observed upon extending the π-system from a pyridyl- (*R*-**1**) to a quinolyl-substituted btz (*R*-**2** and *R*-**3**) (Figure 2 and S15 of the Supporting Information). On a similar note, the increase in intensity of these π–π* ^1^LC bands and the modest bathochromic shifts observed when moving from the proligand to the complex, being, for example, of ca. 1200 cm^–1^ from **L2** to *R*-**3**, are due to the stabilization of the accepting π* levels of the system upon coordination to the Ag­(I) center. Moving to even lower energies, it is possible to note a weak, broad band (λ_abs_ = 380–415 nm, ε = 2 × 10^3^ M^–1^ cm^–1^) which is most prominent in *R*-**2** and *R*-**3**, and can be ascribed to a singlet ligand-to-ligand charge transfer (^1^LLCT, ^1^π_P_ → π_btz_*) transition with little admixing with singlet metal-to-ligand charge transfer (^1^MLCT, ^1^d_π_(Ag) → π_btz_*). The limited ^1^MLCT character is expected due to the little d_p_–π interaction between the Ag­(I) center and the π system of the chromophoric ligand owing to the deeper energy localization of the 4d orbitals.

The six chiral complexes are weakly emissive in both concentrated and diluted, degassed, and air-equilibrated CH_2_Cl_2_. Upon excitation in the lower-lying band (λ_exc_ = 380–420 nm), freshly prepared solutions of all complexes display a very broad and featureless photoluminescence with its maximum in the red to deep-red region (λ_em_ = 610–685 nm) of the visible spectrum (see Table 2, Figure 1 as well as Table S4 and Figure S15 of the Supporting Information). Solutions of complexes **1** and **3** are somewhat photolabile. Regardless, their emission energy and PLQY in degassed dilute CH_2_Cl_2_ were roughly estimated at λ_em_ ≈ 610 nm (PLQY ≈ 0.13%) and λ_em_ ≈ 685 nm (PLQY ≈ 0.38%) for **1** and **3**, respectively (cf. Figure 2). Complex **2** does not seem to be photolabile in solution, but its very low PLQY (<0.1%) renders further time-resolved characterizations in solution unreliable.

On the other hand, all complexes are photostable and highly emissive in the solid state, and were thus further characterized as neat powders, in spin-coated polymer-doped thin films, and in a frozen glassy matrix in CH_2_Cl_2_ at 77 K. Data relative to the emission of *R*-enantiomers of **1**–**3** in powder form and at low temperature are summarized in Table 3 and the corresponding spectra are presented in Figure 3, while the data for complexes *S*-counterparts are found in Table S5 and Figures S16–S17 of the Supporting Information. In the solid state, excited-state lifetimes and PLQYs are significantly lowered by the presence of dioxygen.

**Figure 3:**
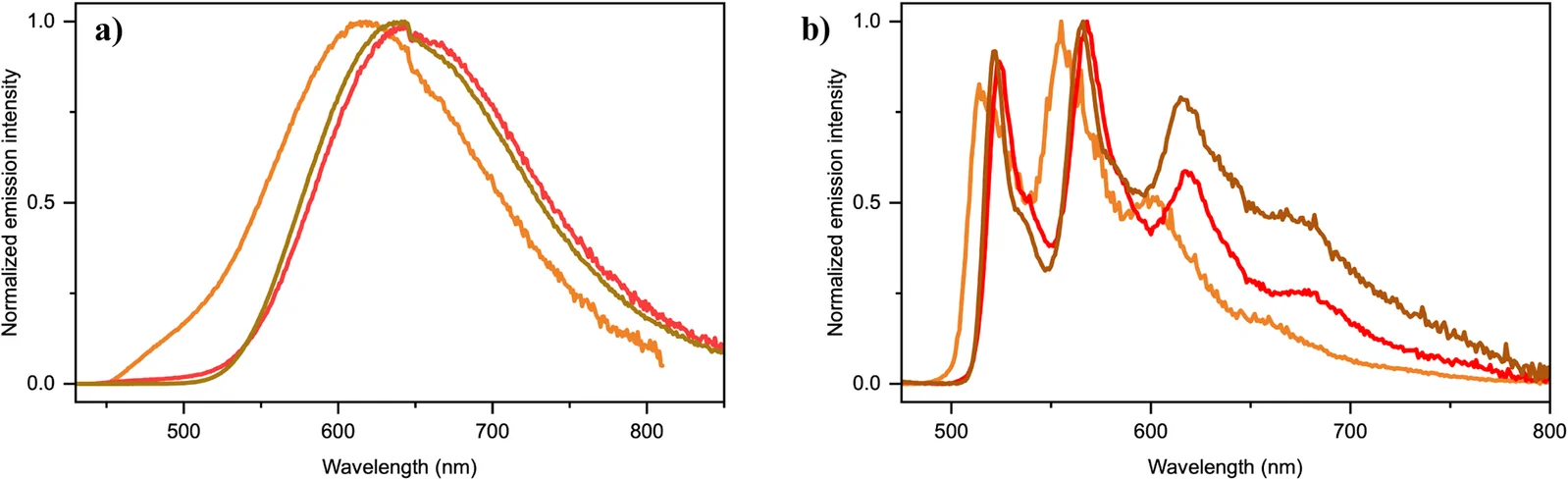
Emission spectra of complexes *R*-**1** (orange), *R*-**2** (red), and *R*-**3** (brown) a) in the solid state as neat powders under an oxygen-free atmosphere at room temperature upon excitation at λ_exc_ = 400 nm and b) in CH_2_Cl_2_ glassy matrix at 77 K upon excitation at λ_exc_ = 400 nm.

**Table 3:** Photophysical Data Recorded for the *R*-Enantiomers of Complexes **1–3** as Neat Powders under Vacuum, in CH_2_Cl_2_ Glassy Matrix at 77 K, and in PMMA 120k Polymer-Doped Spin-Coated Thin Films at 25 wt % under Vacuum (10 mbar)^a^

	λ_em_ [nm]			PLQY (%)		τ̅		
cmpd	*Powder*	77 K (CH_2_Cl_2_)	25 wt % in PMMA	*Powder*	25 wt % in PMMA	*Powder*	77 K (CH_2_Cl_2_)	25 wt % in PMMA
*R-* **1**	620	515, **555**, 600, 650*sh*	585	2	6	6.93 ms	10.9 ms	4.22 ms
*R*-**2**	647	525, 538*sh*, **568**, 585*sh*, 618, 674	615	7	16	5.20 ms	1.93 ms	1.27 ms
*R*-**3**	645	521, 535*sh*, **566**, 582*sh*, 615, 675	620	27	50	7.08 ms	1.89 ms	0.925 ms

a *sh* denotes the shoulder.

The solids were characterized in quartz tubes equipped with a rubber septum where the samples could be kept under dynamic vacuum (ca. 10 mbar) during measurements. In the absence of dioxygen, powders show broad featureless red emission (λ_em_ = 620–650 nm) with average excited-state lifetimes in the range of τ_ave,vac_ as long as 4.30–8.71 ms for all complexes. This photoluminescence can be ascribed to an excited state with admixed ^3^LC­(^3^π_btz_ → π_btz_*)/^3^LLCT (^3^π_P_ → π_btz_*) character. The little involvement of the Ag­(I) center in the radiative process is supported by the very long excited-state lifetime. As a matter of comparison, related Cu­(I) complexes [Cu­(**L1**)­(DPEphos)]­PF_6_ and [Cu­(**L2**)­(DPEphos)]­PF_6_ feature a slightly bathochromically shifted emission (λ_em_ = 622 and 672 nm for the former and the latter, respectively) and much shorter (a few microseconds) lifetimes in the solid state assigned to an ^3^MLCT state.^32^ Similar photophysical data were found by us for red- to near-IR emissive congeners.^12,13^ It is also worth noting that while moving from *R*-**1** to *R*-**2** to *R*-**3**, an enhancement of the PLQY is observed from 2% to 7% to 27%, respectively, which may be attributed to the increase in steric hindrance provided by the extended π-conjugation in the quinolyl-btz compared to the pyridyl group as well as the enhanced bulkiness and rigidity of the xylyl groups in *xyl*-BINAP that provide a locked and tightly packed structure less prone to excited-state molecular distortion that can detrimentally affect the emission properties. This effect is in accordance with previously reported works dealing with Cu­(I) and Ag­(I) complexes bearing α,α′-diimine ligands with *ortho*-substitutions.^11,35,36^ In addition, this explanation resonates also with the observation that complexes **3** could be easily and rapidly crystallized into single crystals suitable for X-ray diffraction studies, whereas complex *S*-**2** required a few months to crystallize. Overall, these data allow us to estimate the radiative rate constant, *k*_r_, by using eq 3 and employing the τ_ave,vac_ value as an estimation, despite the multiexponential decays, as often observed for solid-state samples.

3 $$k_{r}=\frac{PLQY}{\tau }$$

Noteworthy, *k*_r_ values for the T_1_ → S_0_ transition are as slow as 2.3–45 s^–1^ due to the extremely weak SOC effect exerted by the Ag­(I) center, findings that agree nicely with related complexes bearing the dmp ligand reported recently by Yersin and coworkers.^30^

To further elucidate the nature of the emissive excited state, photoluminescence features were recorded in CH_2_Cl_2_ glassy matrix at 77 K. The emission profiles of *R*-enantiomers are displayed in Figure 3. When compared to room temperature, their emission profiles become more structured and are hypsochromically shifted by ca. 1890–2150 cm^–1^, showing the appearance of a vibronic progression typical of heteroaromatic vibrational modes and displaying averaged excited state lifetimes up to τ_avg_ = 11 ms (Table 3). The emission lifetimes at 77 K are shorter than those of the powder samples under vacuum likely due to the presence of dissolved oxygen in the frozen matrix. Similar data are obtained for the *S*-series (see Figure S17 and Table S5 of the Supporting Information). These observations further hint at a large ^3^LC^3^π_btz_ → π_btz_* character of the T_1_ excited state at lower temperatures and rule out TADF as the origin of the emission process.

A considerable switch-on of the emission is further observed in polymer-doped spin-coated thin films (see Figure 4, Figures S18–S21, and Tables S6–S7 of the Supporting Information). Indeed, by dispersing the complexes in PMMA 120k at varying concentrations (5, 25, 50 wt %), it is possible to obtain highly photostable films with broad, featureless, and intense phosphorescence in the yellow-orange to red region, with λ_em,max_ = 585, 615, and 620 nm for complexes *R*-**1**, *R*-**2** and *R*-**3**, respectively. For all complexes, an increase in the doping concentration was accompanied by a slight bathochromic shift, a decrease in the averaged lifetime decay, and a reduction in the PLQY value, indicative of the larger extent of intermolecular interactions between Ag­(I) complexes and subsequent aggregation-caused and triplet–triplet quenching phenomena. Overall, moving down the series, it is possible to obtain a linear shift in the CIE 1931 color coordinates from a yellowish-white to a deep orange emission. Similar to what was observed for powder samples, emission lifetimes and PLQY values were much affected by the presence of dioxygen, whereas emission profiles remained unchanged: repeating the measurements under dynamic vacuum for the best-performing samples (i.e., 25 wt % PMMA films) yielded considerable prolongation of the excited-state lifetimes up to τ_ave,vac_ = 4.22 ms in *R*-**1** and enhancement of the PLQY. Remarkably, the derivatives displaying longer emission wavelengths are also characterized by the higher PLQY values and shorter average excited-state lifetimes, that reach 50% and 0.925 ms, respectively, for *R*-**3**. This finding seems counterintuitive if one considers the energy gap law; nevertheless, this observation can be rationalized by the tighter packing upon increasing the bulkiness of the N^N and P^P ligands that mitigates aggregation-caused quenching phenomena. On the other hand, the increase in PLQY and the concomitant shortening of the excited-state lifetimes, when transitioning from crystalline to doped PMMA films, can be tentatively associated with the adoption of a coordination geometry that provides an emissive excited state characterized by a faster intrinsic radiative rate.

**Figure 4:**
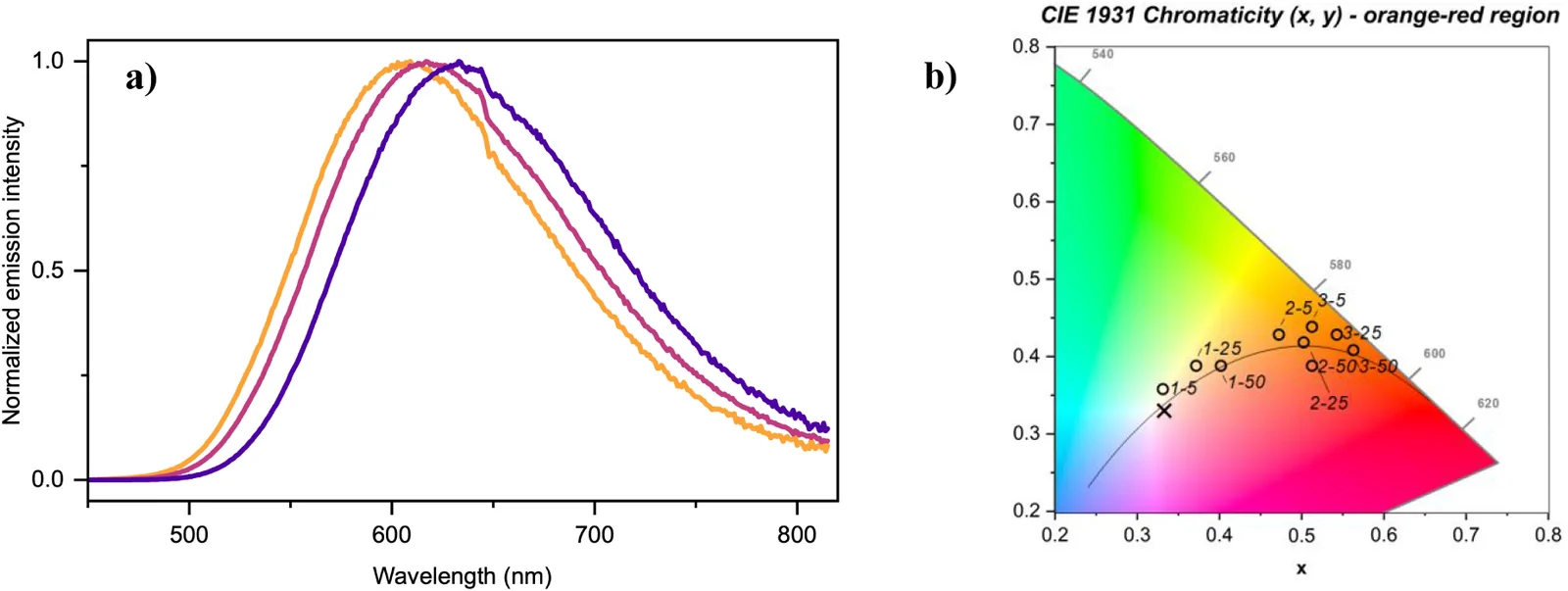
a) Emission spectra of air-equilibrated PMMA 120k polymer-doped spin-coated thin films of complex R-**2** at 5 (orange), 25 (pink), and 50 (violet) wt % concentration upon excitation at λ_exc_ = 420 nm. b) Magnification of the orange-red region of the CIE 1931 diagram displaying the change in color coordinates while moving down the series *R*-**1** > *R*-**2** > *R*-**3** and upon increasing the doping 5 > 25 > 50 wt %. “×” indicates the theoretical white illuminant E (0.33, 0.33).

Given the enantiopure nature of the complexes, their chiroptical properties in polymer-doped spin-coated thin films and in dilute CH_2_Cl_2_ solution were investigated by Electronic Circular Dichroism (ECD) and Circularly Polarized Luminescence (CPL). ECD spectra of complexes *R*-**1**, *R*-**2,** and *R*-**3** in diluted CH_2_Cl_2_ solutions (ca. 1 × 10^–5^ M) are reported in Figure 5, and the corresponding data can be found in Table 4. The spectra show four main peaks at ca. 245 (positive), 265 (negative), 310 (negative), and 345 nm (positive) which partly resemble the spectra of BINAP ligands, both in terms of the sign and sequence of the bands.^37^ Additional weak and broad CD bands can be observed in the range of 370–450 nm, especially for complexes *R*-**2** and *R*-**3**.

**Figure 5:**
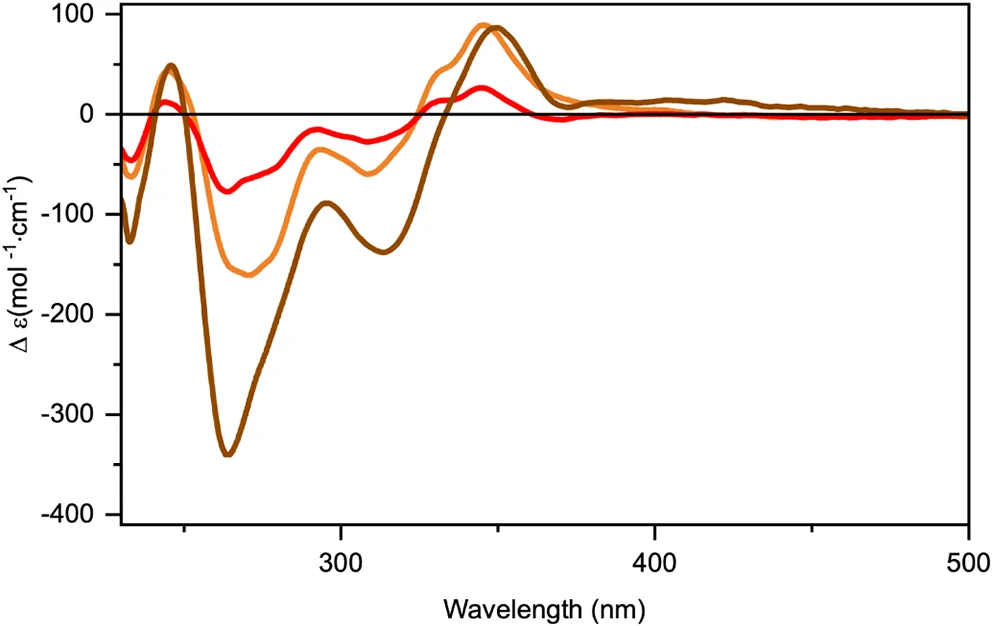
ECD spectra of complexes *R*-**1** (orange), *R*-**2** (red), and *R*-**3** (brown) recorded in diluted CH_2_Cl_2_ solution (ca. 10^–5^ M).

**Table 4:** ECD Data Recorded for the *R*-Enantiomers in Dilute Air-Equilibrated CH_2_Cl_2_ at Room Temperature^a^

cmpd	λ_max_, Δε [nm (M^–1^ cm^–1^)]
*R*-**1**	245 (+42.6), 264*sh* (−151), **271** (−160), 278*sh* (−143), 292 (−35.6), 308 (−59.7), 330*sh* (+39.3), 345 (+89.3)
*R*-**2**	244 (+12.2), **264** (−63.5), 279*sh* (−52.5), 292 (−15.2), 310 (−26.8), 330*sh* (+11.6), 345 (+26.4)
*R* **–3**	233 (−126), 246 (+48.9), **266** (−340), 296 (−88.9), 314 (−137), 349 (+86.2), 408*br* (13.4)

a sh = Shoulder.

When the complexes are dispersed in the PMMA matrix, the ECD spectra show some changes in the signal sequences and in the relative intensity of the peaks. The ECD spectra of PMMA thin films at 5, 25, and 50 wt % doping level are displayed in Figure 6. For complex *R*-**1**, the increase in its concentration inside the polymeric thin films causes an increase in intensity and a bathochromic shift of the lowest energy band, suggesting the presence of aggregates of complex *R*-**1** inside the PMMA matrix, in line with the variation of the photophysical data discussed above. The same trend can also be appreciated for complexes *R*-**2** and *R*-**3**, where the increase in the complex concentration inside the PMMA films translate in the appearance of positive bands in the region 380–450 nm, indicating the formation of chiral aggregates of the complexes.

**Figure 6:**
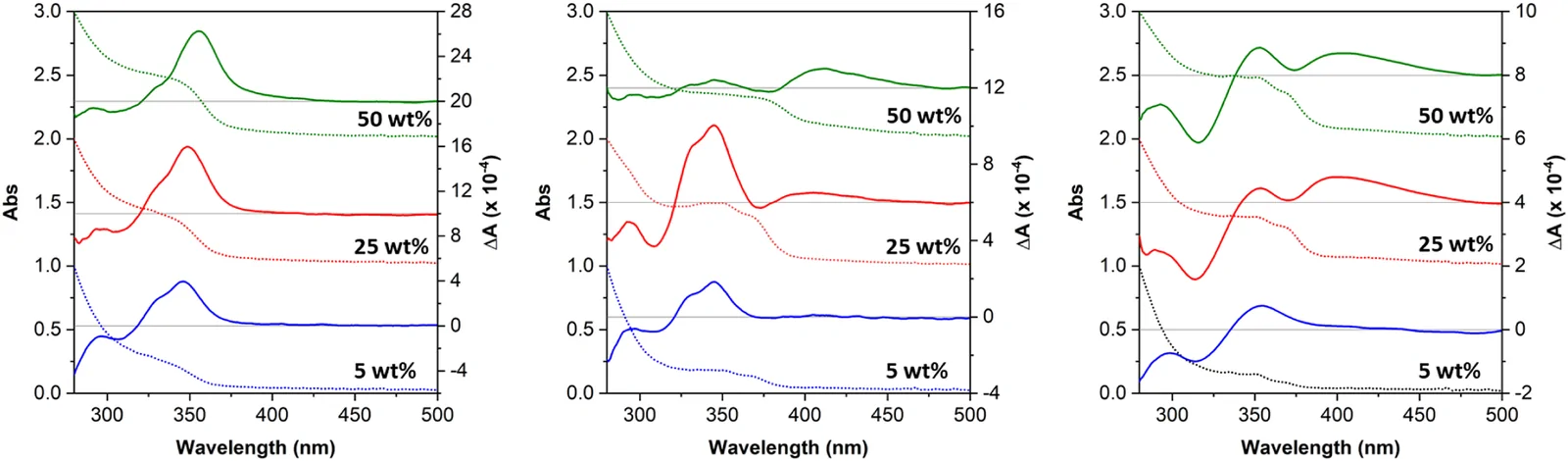
ECD (solid lines) and absorption spectra (dashed lines) of complexes *R*-**1** (left), *R*-**2** (middle), and *R*-**3** (right) recorded in thin PMMA films at 5 (blue lines), 25 (red lines), and 50 wt % (green lines) concentrations. For clarity, the spectra are reported with an offset.

Unfortunately, no CPL could be detected in thin-film samples at any of the investigated concentrations, irrespective of the postfabrication (thermal and solvent annealing) process.

### Computational Investigation

To further elucidate the photophysical behavior of these chiral Ag­(I) emitters, ground-state geometries, absorption spectra, and excited singlet and triplet states have been computed for *R*-**1**, *R*-**2**, and *R*-**3**.

Computed absorption spectra for *R*-**1**, *R*-**2,** and *R*-**3** display features overall similar to those of their achiral congeners (Figure 7, Tables S8 and S9 of the SI).^31^ In particular, the computed absorption of *R*-**1** is nearly identical to that of its Xantphos and DPEphos congeners and will not be discussed further (see Supporting Information). The computed absorption spectra of *R*-**2** and *R*-**3** are bathochromically shifted by 20–30 nm, with the S_0_,→S_1_ transition of mostly metal-perturbed ^1^LLCT character yielding a weak band in the visible domain at *ca*. 475 nm as shown by THEODore analysis (see Figure 8 and Figure S28 of the SI). For *R*-**2**, the S_0_ → S_2_ and S_0_ → S_3_ transitions yield a weaker shoulder at 400 nm and have the same nature, while the intense band around 350 nm is mainly due to the S_0_ → S_5_ transition admixed with S_0_ → S_4_, both of mainly ^1^LC character, albeit located on the N^N and P^P ligands, respectively. The picture is somewhat different for *R*-**3**, as the intense band at *ca*. 350 nm is generated by the convolution of the S_0_ → S_6_ (^1^LC_P^P_) with the S_0_ → S_7_, S_0_ → S_9_, and S_0_ → S_10_ transitions which are of admixed ^1^LLCT and ^1^LC_btz_ character.

**Figure 7:**
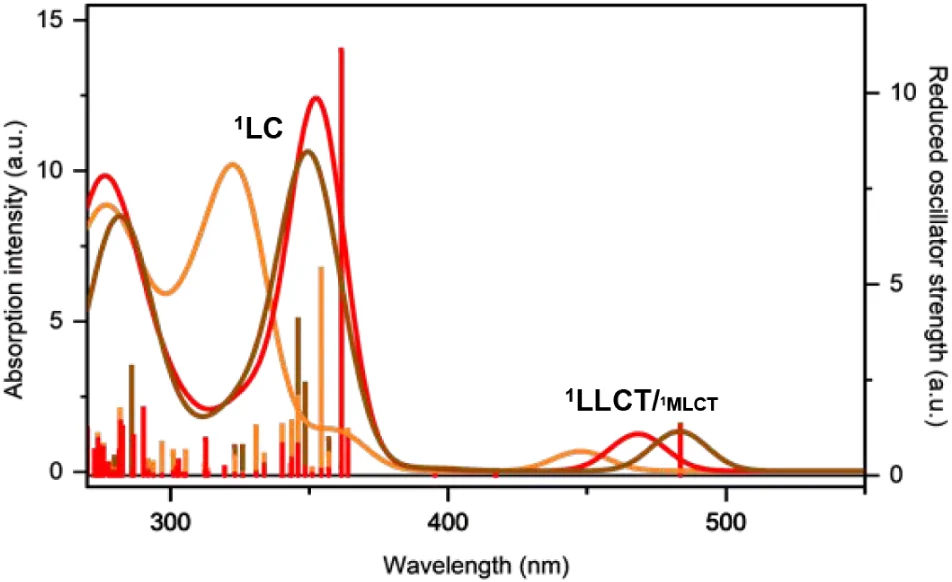
Computed electronic absorption spectra and oscillator strengths in CH_2_Cl_2_ for complexes *R*-1 (orange), *R*-**2** (red), and *R*-**3** (brown).

**Figure 8:**
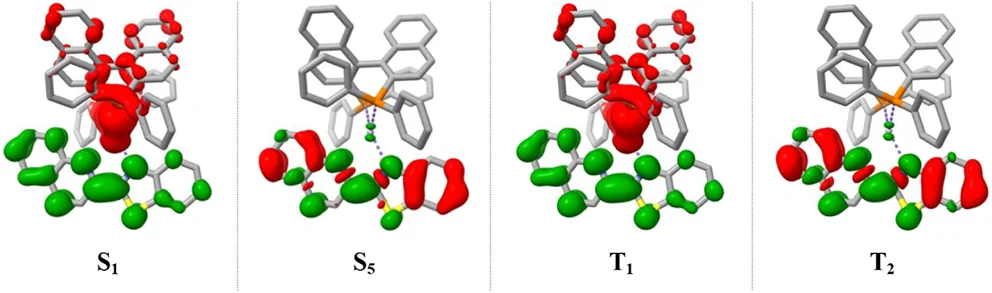
EDDMs between the ground state and the S_1_, S_5_, T_1_, and T_2_ excited states of R-**2**. Electronically depleted areas are in red and enriched areas are in green.

At the Franck–Condon geometry, the lowest T_1_ state of the three complexes is a metal-perturbed ^3^LLCT state involving CT from the lone pairs of the phosphorus atoms to a π* orbital on the benzothiazole with admixed ^3^LC/^3^MLCT character (Figure 8). For *R*-**2** and *R*-**3**, T_2_ is an almost pure ^3^LC state on the N^N ligand, while T_3_ is an admixed ^3^LLCT state with considerable ^3^LC character on the diphosphine. For *R*-**1**, T_2_ and T_3_ have mainly ^3^LC character on the BINAP, while T_4_ is the first ^3^LC state on the N^N ligand. The computed emission wavelengths for T_1_ are around *ca*. 865 nm, inconsistent with the experimental results: this suggests that the T_2_ state of ^3^LC_btz_ nature is likely the one responsible for the emission and that T_1_ acts as a quenching state, as is the case in similar systems.^28^ This would explain the very low estimated PLQY values in solution, which are in the 0.1–0.4% range.

The theoretical ECD spectra are computed for the three complexes and are displayed in Figure 9. The corresponding data are listed in Tables S10–S11. The computed spectra agree well with the experimental data presented in Figure 4 and overall present a similar shape, with the S_0_ → S_1_ transition (absent in the experimental spectra) visible as a weak, low-energy peak at *ca*. 470 nm. As far as the computed ECD spectrum of *R*-**3** is concerned, the first intense signal around λ_abs_ = 360 nm is mainly due to the first metal-perturbated ^1^LC_P^P_ transition (cf. S_0_ → S_6_, Figure 10) and exhibits a positive polarization bias. The following peak, of negative bias, is localized around λ_abs_ = 330 nm and is the convolution of several electronic transitions with a major contribution from the S_0_ → S_17_ transition, which is another metal-perturbated ^1^LC_P^P_ transition. The third intense band localized around λ_abs_ = 290 nm is mainly due to the S_0_ → S_33_ transition, again of ^1^LC_P^P_ nature.

**Figure 9:**
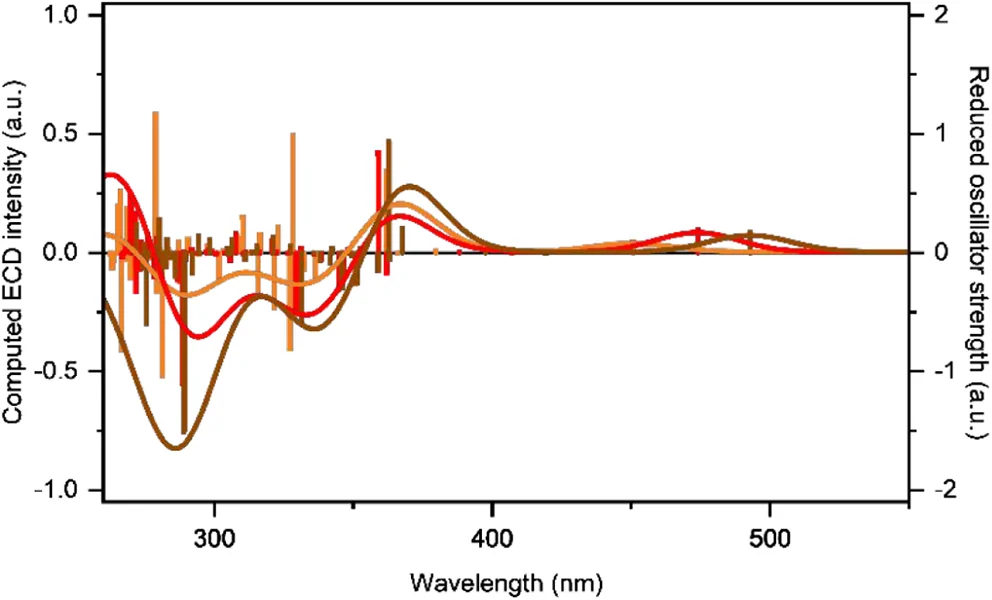
Computed electronic circular dichroism spectra and oscillator strengths in CH_2_Cl_2_ for complexes *R*-**1** (orange), *R*-**2** (red), and *R*-**3** (brown).

**Figure 10:**
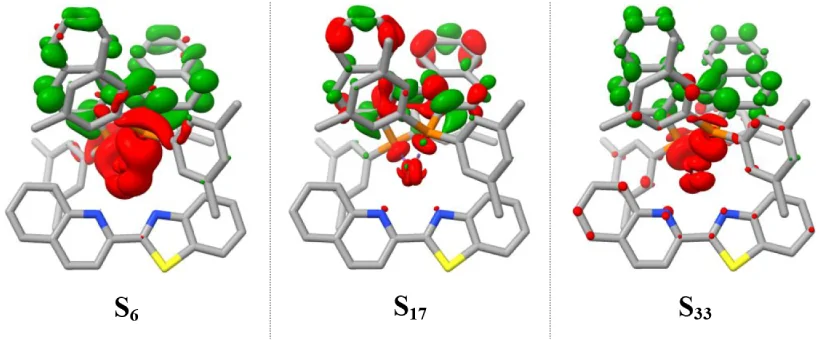
EDDMs between the ground state and the S_6_, S_17_, and S_33_ excited states of *R*-**3**. Electronically depleted areas are in red and enriched areas are in green.

Since the potentially emissive states in these compounds (i.e., ^3^LC_btz_ and metal-perturbed ^3^LLCT states) do not involve the chiral diphosphine, their rotatory strength is expected to be small. This is further supported by the negligible rotatory strength in ECD measurements of their singlet state counterparts (i.e., the weak ECD signal of the metal-perturbed ^1^LLCT states between 450–500 nm in Figure 9). As a consequence, no CPL emission is observed.

## Conclusions

The synthesis of a family of novel chiral mononuclear Ag­(I) complexes of general structure [Ag^I^(N^N)­(P^P)]^+^, where N^N is a pyridyl- or quinolyl-functionalized benzothiazole and P^P is an enantiopure BINAP or *xyl*BINAP diphosphine, was herein presented. Compared to their achiral Xantphos and DPEphos analogues,^31^ these six novel chiral complexes are not as stable or emissive in solution, but display a bright bathochromically shifted emission profile in the solid state, with complexes **3** reaching values as high as λ_em,max_ *ca*. 650 nm with PLQY = 30% in powder samples and 50% in spin-coated polymer-doped thin films. The better performance of the quinoline-functionalized benzothiazole ligand was assessed as well as the importance of a bulky bidentate diphosphine such as *xyl*BINAP to enhance the PLQY through solid-state aggregation. Lastly, the chiroptical properties of these complexes were investigated, but while solution and solid-state ECD spectra detected features typical of BINAP derivatives, no CPL could be detected due to the emissive states being of mostly metal-perturbed ligand-centered character localized on the achiral N^N ligand. These six complexes are rare examples of chiral emissive Ag­(I) complexes, and their intense deep-red emission in the solid state is a promising advancement toward the development of efficient and environmentally friendly Ag­(I) deep-red and NIR emitters.

## Supplementary Material



## Notes

Crystallographic structures, additional absorption and emission data, thermogravimetric data, additional computational data, and NMR spectra (PDF)
