# Supplementary material for: Millisecond-Long Red Phosphorescence in Chiral Heteroleptic Ag(I) Complexes Bearing Benzothiazole Ligands
Source: Inorg Chem. 2026 Aug 6;65(32):18554–62. doi: 10.1021/acs.inorgchem.6c01416 (PMC13488390; doi:10.1021/acs.inorgchem.6c01416)
Supplement: Supplementary file 1 [file ic6c01416_si_001.pdf]

Electronic Supplementary Information

for

Millisecond-long red phosphorescence in chiral heteroleptic Ag(I) complexes bearing benzothiazole ligands

Valerio Giuso,[a] Christophe Gourlaouen,[b] Lorenzo Arrico,[c] Lorenzo Di Bari,[c] and Matteo Mauro[a,d,*]

[a] *Université de Strasbourg & CNRS, Institut de Physique et Chimie des Matériaux de Strasbourg UMR 7504, F-67034 Strasbourg, France*
[b] *Laboratoire de Modélisation et Simulations Moléculaires, UMR7140 Chimie de la Matière Complexe, Institut Le Bel, F-67081 Strasbourg, France*
[c] *Dipartimento di Chimica e Chimica Industriale, Università di Pisa, Via Moruzzi 13, 56124 Pisa, Italy*
[d] *Department of Chemical Sciences, University of Padova, Via Marzolo 1, 35121 Padova, Italy*
* Corresponding author: matteo.mauro@unipd.it

Contents

Experimental section 4
General synthetic remarks 4
Supplementary procedures 5
NMR Spectra 7
ESI-HR Spectra 11
XRD and SWAXS data 13
Photophysical measurements 17
Instrument details 17
Methods 17
Supplementary photophysical data 18
Chiroptical measurements 23
Instrument details and methods 23
Thermogravimetric measurements 23
Computational studies 27
References 33

Schemes, figures and tables

Scheme S1. Schematic synthetic pathway employed for [Ag(**L1–L2**)(P^P)]PF_6^- complexes. . 4

Figure S1. ^{1}H NMR (500 MHz, CD_2Cl_2) spectrum of complex *R*-**1**. 7
Figure S2. ^{13}C{^{1}H} DEPT-135 NMR (126 MHz, CD_2Cl_2) spectrum of complex *R*-**1**. 7
Figure S3. ^{31}P{^{1}H} NMR (202 MHz, CD_2Cl_2) spectrum of complex *R*-**1**. 8
Figure S4. ^{1}H NMR (500 MHz, CD_2Cl_2) spectrum of complex *R*-**2**. 8
Figure S5. ^{13}C{^{1}H} DEPT-135 NMR (126 MHz, CD_2Cl_2) spectrum of complex *R*-**2**. 9
Figure S6. ^{31}P{^{1}H} NMR (202 MHz, CD_2Cl_2) spectrum of complex *R*-**2**. 9
Figure S7. ^{1}H NMR (500 MHz, CD_2Cl_2) spectrum of complex *R*-**3**. 10
Figure S8. ^{13}C{^{1}H} DEPT-135 NMR (126 MHz, CD_2Cl_2) spectrum of complex *R*-**3**. 10
Figure S9. ^{31}P{^{1}H} NMR (202 MHz, CD_2Cl_2) spectrum of *R*-**3**. 11
Figure S10. HR-MS-ESI spectrum recorded for complex *R*-**1**. 11
Figure S11. HR-MS-ESI spectrum recorded for complex *R*-**2**. 12
Figure S12. HR-MS-ESI spectrum recorded for complex *R*-**3**. 12
Figure S13. ORTEP diagram of *S*-**2** shown at 50% probability. Hydrogen atoms and the PF_6^- counter anion are omitted for clarity. 13
Figure S14. Powder SWAXS of complexes **1**–**3**. Complexes **1**–**2** are amorphous, complexes **3** are semicristalline. The crystalline fractions in *R*-**3** and *S*-**3** have the same structure. 16
Figure S15. Electronic absorption (solid) and emission (dashed) spectra of complexes *S*-**1** (orange), *S*-**2** (red), *S*-**3** (brown) in dilute (2·10^{-5} M) degassed CH_2Cl_2 at room temperature upon excitation at λ_{exc} = 350, 360, and 370 nm respectively. 19
Figure S16. Emission spectra of complexes *S*-**1** (orange), *S*-**2** (red), and *S*-**3** (brown) in the solid state as neat powders under oxygen-free atmosphere at room temperature upon excitation at λ_{exc} = 400 nm. 19
Figure S17. Emission spectra of complexes *S*-**1** (orange), *S*-**2** (red), and *S*-**3** (brown) in CH_2Cl_2 glassy matrix at 77 K upon excitation at λ_{exc} = 400 nm. 19
Figure S18 Emission spectra of complexes *R*-**1** and *R*-**3** in air-equilibrated PMMA 120k polymer-doped spin-coated thin films. *R*-**1** at 5 (teal), 25 (blue), and 50 (violet) wt.% concentration and *R*-**3** at 5 (purple), 25 (orange), and 50 (yellow) wt.% concentration upon excitation at λ_{exc} = 420 nm. 20
Figure S19. Emission spectra of complexes *S*-**1** in air-equilibrated PMMA 120k polymer-doped spin-coated thin films at 5 (green), 25 (teal), and 50 (blue) wt.% concentration upon excitation at λ_{exc} = 420 nm. 20
Figure S20. Emission spectra of complexes *S*-**2** in air-equilibrated PMMA 120k polymer-doped spin-coated thin films at 5 (orange), 25 (violet), and 50 (blue) wt.% concentration upon excitation at λ_{exc} = 420 nm. 20
Figure S21. Emission spectra of complexes *S*-**3** in air-equilibrated PMMA 120k polymer-doped spin-coated thin films at 5 (orange), 25 (violet), and 50 (blue) wt.% concentration upon excitation at λ_{exc} = 420 nm. 21
Figure S22. TGA curve of *R*-**1**. The product contains a volatile amount released between ca. 120 and 180°C. Significant weight loss from starting degradation observed above ca. 210°C. 5% weight loss temperature at 5°C/min: $T_{5\%}$ = 256°C (± 3°C). 24
Figure S23. TGA curve of *S*-**1**. The product contains a small volatile amount released below 180°C. Significant weight loss from starting degradation observed above ca. 220°C. 5% weight loss temperature at 5°C/min: $T_{5\%}$ = 255°C (± 3°C). 24
Figure S24. TGA curve of *R*-**2**. Significant weight loss from starting degradation observed above ca. 200°C. 5% weight loss temperature at 5°C/min: $T_{5\%}$ = 259°C (± 5°C). 25

Figure S25. TGA curve of *S*-**2**. Significant weight loss from starting degradation observed above ca. 190°C. 5% weight loss temperature at 5°C/min: $T_{5\%}$ = 240°C (± 3°C). 25
Figure S26. TGA curve of *R*-**3**. A sample measured after a preliminary drying at 180°C under nitrogen flux displayed significant weight loss from starting degradation above ca. 190°C. 5% weight loss temperature at 5°C/min: $T_{5\%}$ = 266°C (± 3°C). 26
Figure S27. TGA curve of *S*-**3**. A sample measured after a preliminary drying at 180°C under nitrogen flux displayed significant weight loss from starting degradation above ca. 190°C. 5% weight loss temperature at 5°C/min: $T_{5\%}$ = 271°C (± 3°C). 26
Figure S28. Theodore analysis of the singlet and triplet states of the three complexes computed at Franck-Condon geometry within the TDA approximation. Lp stands for the P^P ligand and Ln for the N^N ligand. 29

Table S1. Crystal data and structure refinement for complex *S*-**2**. 13
Table S2. Crystal data and structure refinement for complex *R*-**3**. 14
Table S3. Crystal data and structure refinement for complex *S*-**3**. 15
Table S4. Photophysical data recorded for complexes *S*-**1*S***, *S*-**2** and *S*-**3** in dilute degassed CH_2Cl_2 at room temperature. *sh* denotes a shoulder, *dec.* denotes photodecomposition. 18
Table S5. Photophysical data recorded for complexes *S*-**1**, *S*-**2** and *S*-**3** as neat powders under vacuum, in CH_2Cl_2 glassy matrix at 77 K, and in PMMA 120k polymer-doped spin-coated thin films at 25 wt.% under vacuum. *sh* denotes a shoulder, *br* denotes a broad signal. 18
Table S6. Photophysical data for *R*-**1**, *R*-**2** and *R*-**3** as air-equilibrated polymer-doped spin-coated thin films. 21
Table S7. Photophysical data for *S*-**1**, *S*-**2** and *S*-**3** as air-equilibrated polymer-doped spin-coated thin films. 22
Table S8. Absorption energies (E_{abs} in eV), absorption wavelength (λ_{abs} in nm) and oscillator strength (f_{osc}) of the computed singlet states of *R*-**1**, *R*-**2** and *R*-**3** within the TDA approximation. 27
Table S9. Absorption energies (E_{abs} in eV) and absorption wavelength (λ_{abs} in nm) of the computed triplet states of *R*-**1**, *R*-**2** and *R*-**3** within the TDA approximation. 28
Table S10. Absorption energies (E_{abs} in eV), absorption wavelength (λ_{abs} in nm), oscillator strength (f_{osc}) and Rotatory strength (R) of the computed singlet states of *R*-**1** without the TDA approximation. 30
Table S11. Absorption energies (E_{abs} in eV), absorption wavelength (λ_{abs} in nm), oscillator strength (f_{osc}) and Rotatory strength (R) of the computed singlet states of *R*-**2** without the TDA approximation. 31
Table S12. Absorption energies (E_{abs} in eV), absorption wavelength (λ_{abs} in nm), oscillator strength (f_{osc}) and Rotatory strength (R) of the computed singlet states of *R*-**3** without the TDA approximation. 32

Experimental section

General synthetic remarks

All reagents were used as received without further purification unless differently stated. ^{1}H and ^{13}C NMR spectra were recorded at 298 K on Bruker AV500 spectrometers in deuterated solvents and the residual solvent peak was used as the internal reference. ^{1}H and ^{13}C{^{1}H} NMR spectra were calibrated to residual solvent signals. All the chemical shifts (δ) are reported in ppm and coupling constants in Hz.

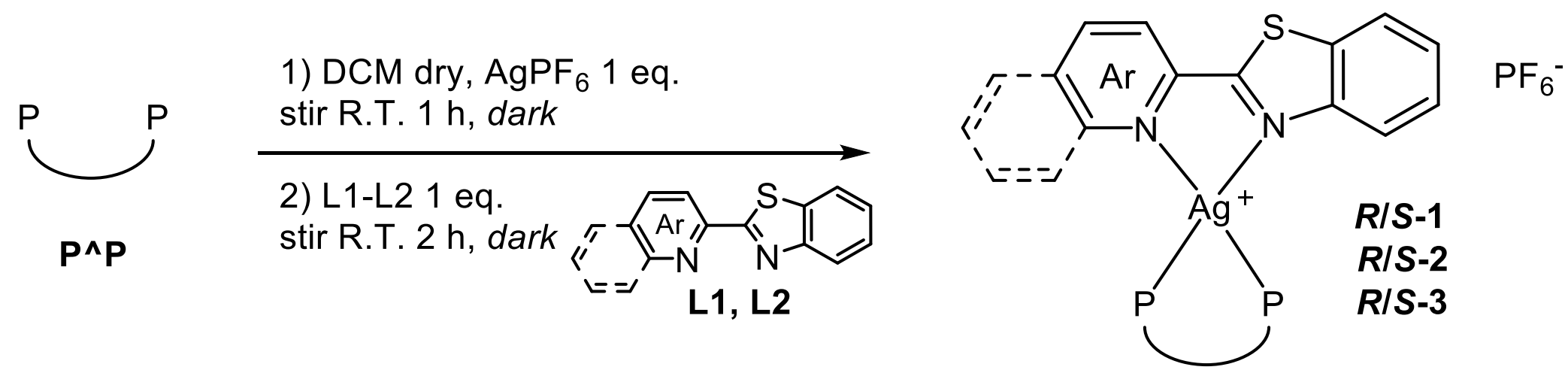

***R*-1** **(89%)** P^P = *R*-BINAP Ar = pyridine
***S*-1** **(90%)** P^P = *S*-BINAP Ar = pyridine
***R*-2** **(92%)** P^P = *R*-BINAP Ar = quinoline
***S*-2** **(93%)** P^P = *S*-BINAP Ar = quinoline
***R*-3** **(64%)** P^P = *R*-*xyl*BINAP Ar = quinoline
***S*-3** **(74%)** P^P = *S*-*xyl*BINAP Ar = quinoline

Scheme S1. Schematic synthetic pathway employed for [Ag(**L1**–**L2**)(P^P)]PF_6^- complexes.

All silver complexes were prepared according the same general procedure, employing anhydrous $CHCl_2$, in glassware shielded from light, and at room temperature (Scheme S1). As the ^{1}H, ^{13}C{^{1}H}, and ^{31}P{^{1}H} NMR spectra and MS-ESI peaks obtained for each pair of enantiomers *R*-**1**, *S*-**1**, *R*-**2** *S*-**2**, and *R*-**3** and *R*-**3** are identical, only the data relative to the *R* enantiomers is presented in full. The ^{31}P{^{1}H} NMR spectra of the complexes recorded in CD_2Cl_2 are often characterized by two doublets ($^{1}J_{H\text{-}P}$ = 200-400 Hz) of about 1:1 intensity at d *ca.* -15 ppm owing to the coupling with the two NMR active nuclei of the Ag isotopes, namely ^{107}Ag and ^{109}Ag, of natural abundance of 52% and 48%, respectively. The formation of homoleptic Ag(I)-phosphine complexes can be detected by ^{31}P{^{1}H} NMR, with a second pair of signals appearing; this, however, is not noticeable in ^{1}H and ^{13}C{^{1}H} NMR spectra, and does not seem to interfere with the photophysical studies. Additionally, due to the labile nature of the species in solution, the base peak observed in all MS-ESI spectra is relative to the Ag(P^P)$^+$ species, $C_{44}H_{32}P_2Ag^+$ (calcd 729.1030 m/z) for BINAP and $C_{52}H_{48}P_2Ag^+$ (calcd 841.2282 m/*z*) for *xyl*BINAP. The molecular peaks are thus reported with their relative % intensity.

Supplementary procedures

General synthetic procedure of [Ag(L1–L2)(P^P)]PF_6 complexes

In a 50 mL round bottom flask covered with aluminium foil were added dry dichloromethane (10 mL), $AgPF_6$ (0.10-0.40 mmol, 1 equiv.), and the desired phosphine (0.10-0.40 mmol, 1 equiv). After stirring the colourless solution for 1 h, the ligand was added (0.10-0.40 mmol, 1 equiv.) with the aid of dry dichloromethane (10 mL) and the solution was left to stir for 2 hours. The resulting yellow solution was added dropwise to a large volume of petroleum ether (500 mL) under heavy stirring, which caused the desired complex to flocculate instantly. The complex was collected by filtration on a G4 Gooch funnel, and cleaned by repeated washing with petroleum ether (5×20 mL). All complexes were obtained in excellent to near-quantitative yields with sufficient purity for chemical and photophysical characterizations.

*Complexes [Ag(**L1**)(R-BINAP)]PF_6 (R-**1**) and [Ag(**L1**)(S-BINAP)]PF_6 (S-**1**)*

White powders (*R*-**1**: 340 mg, 0.312 mmol, yield 89%; *S*-**1**: 334 mg, 0.306 mmol, yield 90%). ^{1}H NMR (500 MHz, 298 K, CD_2Cl_2) δ: 8.09 (1 H, m), 7.87 (2 H, m), 7.64–7.45 (10 H, m), 7.40-7.18 (11 H, m), 7.09 (1 H, ddd, *J* 8.3, 6.8, 1.3), 7.06–6.96 (6 H, m), 6.89 (1 H, dd, *J* 8.6, 1.0), 6.69-6.79 (3 H, m), 6.56 (2 H, t, *J* 7.6), 6.40 (1 H, tt, *J* 7.5, 1.1), 5.95 (2 H, t, *J* 7.6). ^{13}C{^{1}H} NMR DEPT135 (125.7 MHz, 298 K, CD_2Cl_2) δ: 136.3, 134.7, 133.4, 131.4, 130.9, 129.9, 129.3, 129.0, 128.8, 128.1, 127.9, 127.9, 127.6, 127.2, 126.9, 126.8, 126.7. ^{31}P{^{1}H} NMR (202 MHz, 298 K, CD_2Cl_2) δ: 15.15 (dd, $^1J_{Ag\text{-}P}$ = 241.5, $^2J_{P\text{-}P}$ = 17.4), -144.4 (*hept*, PF_6^-). ESI-MS calcd for $C_{56}H_{40}AgN_2P_2S^+$: 943.1439. Found for ([M -PF_6]$^+$): 943.1436 (13.3%).

*Complexes [Ag(**L2**)(R-BINAP)]PF_6 (R-**2**) and [Ag(**L2**)(S-BINAP)]PF_6 (S-**2**)*

Yellow powders (*R*-**2**: 416 mg, 0.365 mmol, yield 92%; *S*-**2**: 421 mg, 0.370 mmol, yield 93%). ^{1}H NMR (500 MHz, 298 K, CD_2Cl_2) δ: 8.71 (1 H, d, *J* 8.5), 8.34 (1 H, d, *J* 8.3), 8.22 (1 H, s), 8.17 – 8.02 (2 H, m), 7.96 (1 H, d, *J* 3.1), 7.76 (2 H, d, *J* 8.7), 7.73–7.64 (4 H, m), 7.63–7.53 (5 H, m), 7.50–7.35 (2 H, m), 7.31 (2 H, t, *J* 7.6), 7.21–7.05 (11 H, m), 6.98 (1 H, dd, *J* 8.6, 1.0), 6.90 (2 H, d, *J* 8.6), 6.81 (2 H, t, *J* 7.5), 6.56 (3 H, t, *J* 7.6), 6.48 (1 H, tt, *J* 7.5, 1.1), 6.04 (1 H, t, *J* 7.6). ^{13}C{^{1}H} NMR DEPT135 (125.7 MHz, 298 K, CD_2Cl_2) δ: 140.6, 136.3, 135.0, 134.9, 134.8, 133.4, 133.3, 133.2, 133.2, 131.9, 131.5, 130.9, 130.3, 129.8, 129.4, 129.3, 129.1, 129.1, 128.8, 128.1, 128.1, 127.8, 127.5, 127.3, 127.2, 127.2, 126.9, 126.8, 126.7, 123.5, 123.2, 121.4. ^{31}P{^{1}H} NMR (202 MHz, 298 K, CD_2Cl_2) δ: 15.16 (dd, $^1J_{Ag\text{-}P}$ = 241.5, $^2J_{P\text{-}P}$ = 16.6), -144.4 (*hept*, PF_6^-). ESI-MS calcd for $C_{60}H_{42}AgN_2P_2S^+$: 991.1594. Found for ([M -PF_6]$^+$): 991.1512 (6.91%).

*Complexes [Ag(**L2**)(R-xylBINAP)]PF₆ (R-**3**) and [Ag(**L2**)(S-xylBINAP)]PF₆ (S-**3**)*

Yellow powders (*R*-**3**: 133 mg, 0.102 mmol, yield 64%; *S*-**3**: 175 mg, 0.134 mmol, yield 74%).^{1}H NMR (500 MHz, 298 K, CD_2Cl_2) *δ*: 8.72 (1 H, d, *J* 8.4), 8.39 (1 H, d, *J* 8.4), 8.22 (1 H, d, *J* 8.1), 8.12 (1 H, d, *J* 8.2), 7.90 – 7.10 (20 H, m), 6.99 – 6.60 (8 H, m), 6.34 (2 H, s), 1.78 (24 H, s). ^{13}C{^{1}H} NMR DEPT135 (125.7 MHz, 298 K, CD_2Cl_2) *δ*: 132.9, 132.8, 132.6, 132.6, 131.9, 131.9, 131.8, 131.3, 130.5, 129.3, 128.8, 128.7, 127.9, 127.7, 127.6, 127.1, 126.8, 126.2, 124.7, 123.9, 123.1, 20.6. ^{31}P{^{1}H} NMR (202 MHz, 298 K, CD_2Cl_2) δ: 14.97 (d, $^{1}J_{Ag\text{-}P}$ = 366.5), -144.4 (PF_6^-). ESI-MS calcd for $C_{68}H_{58}AgN_2P_2S^+$: 1103.2846. Found for ($[M -PF_6]^+$): 1803.2814 (19.6%).

NMR Spectra

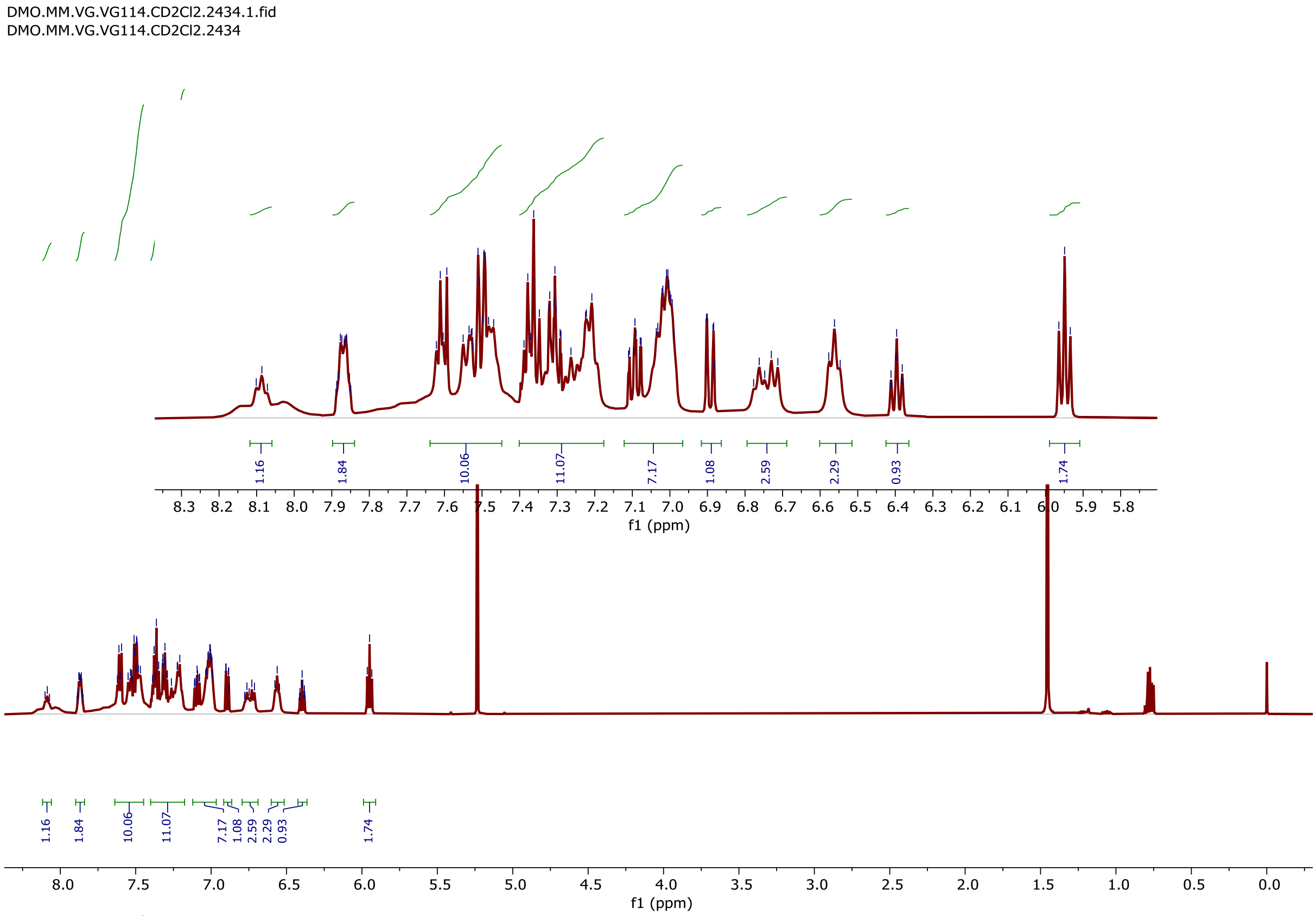

Figure S1. ^{1}H NMR (500 MHz, CD_2Cl_2) spectrum of complex *R*-**1**.

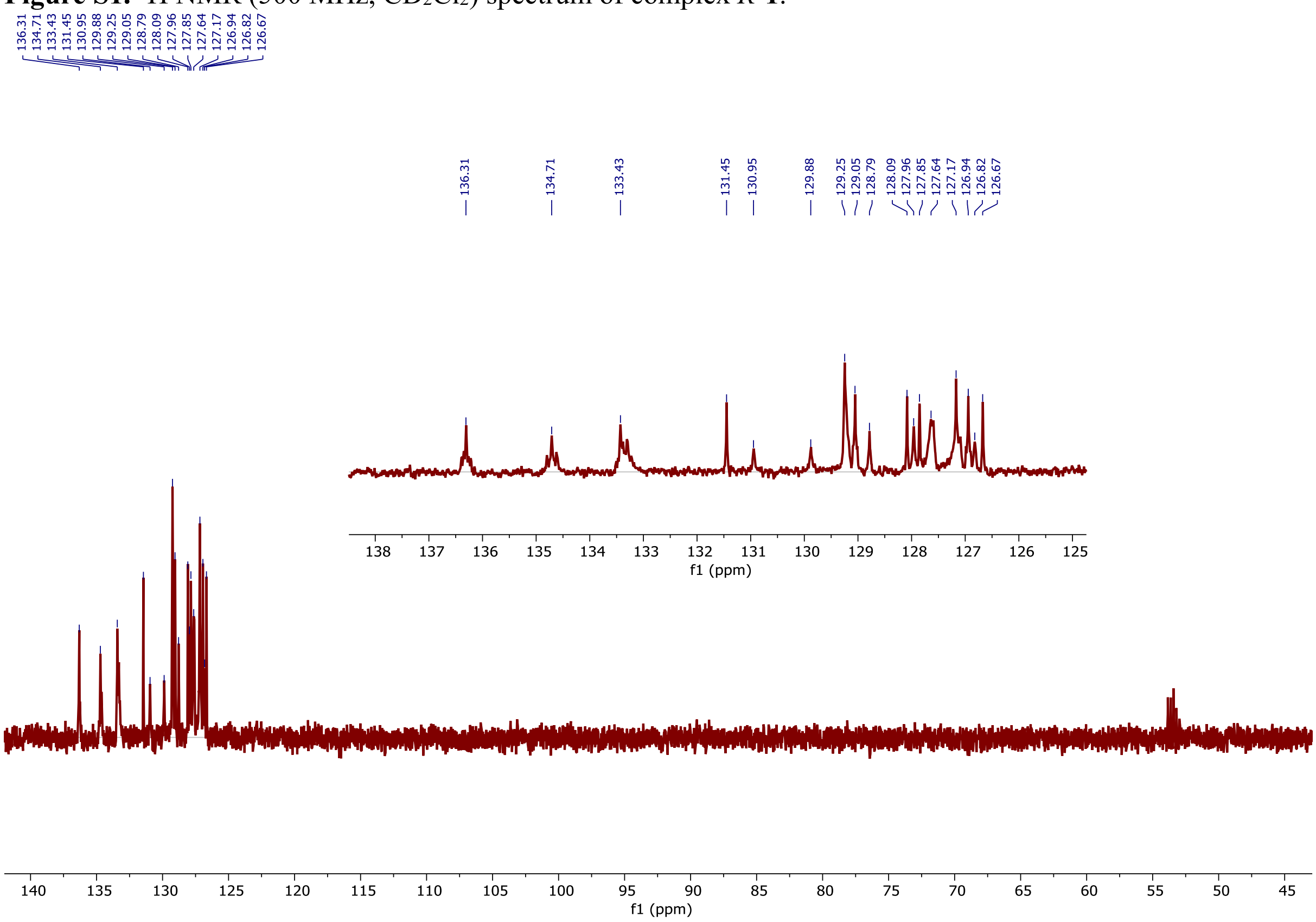

Figure S2. $^{13}C\{^{1}H\}$ DEPT-135 NMR (126 MHz, CD_2Cl_2) spectrum of complex *R*-**1**.

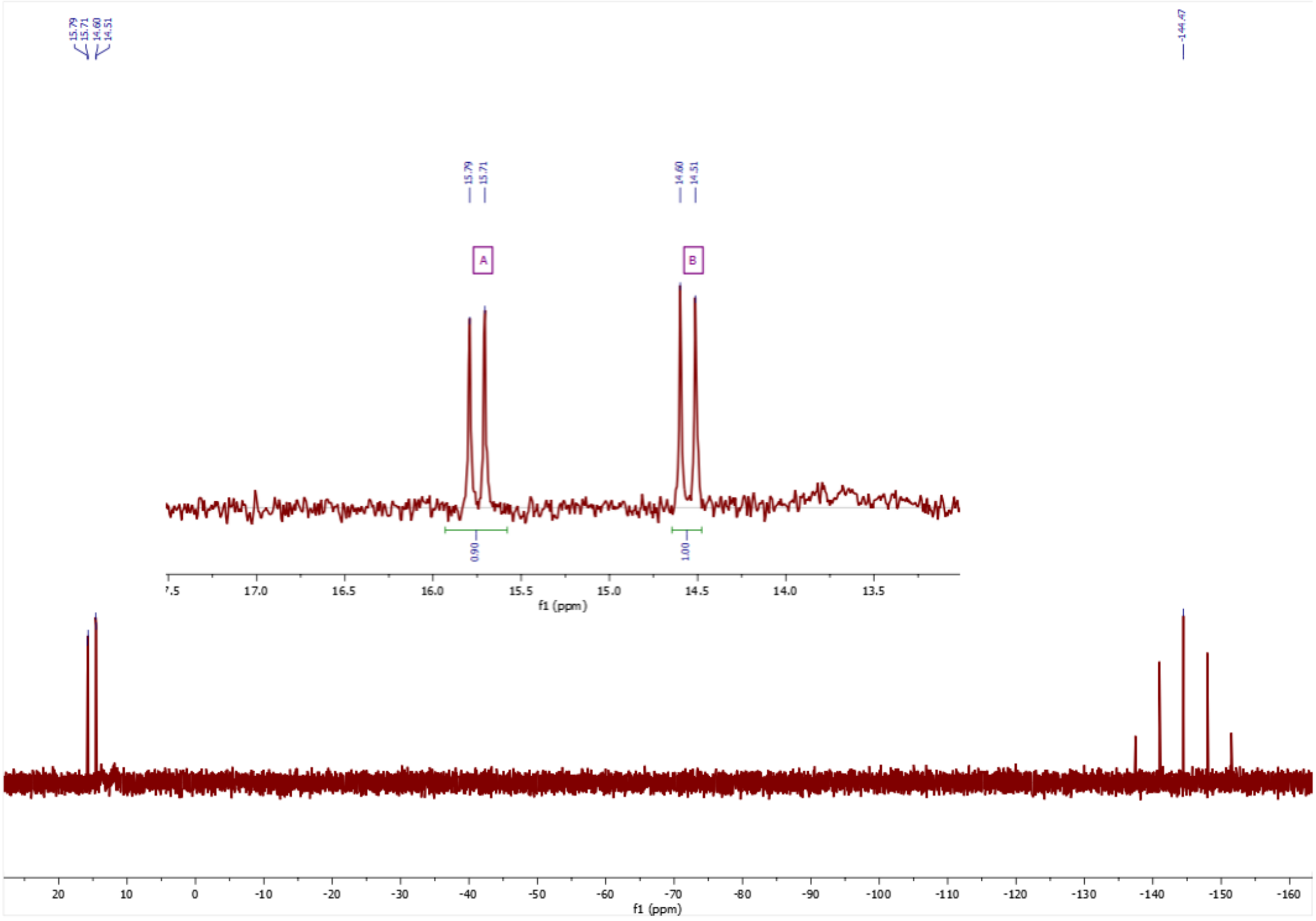

Figure S3. $^{31}P\{^{1}H\}$ NMR (202 MHz, CD_2Cl_2) spectrum of complex *R*-**1**.

DMO.MM.VG.VG126.CD2Cl2.2449.1.fid
DMO.MM.VG.VG126.CD2Cl2.2449

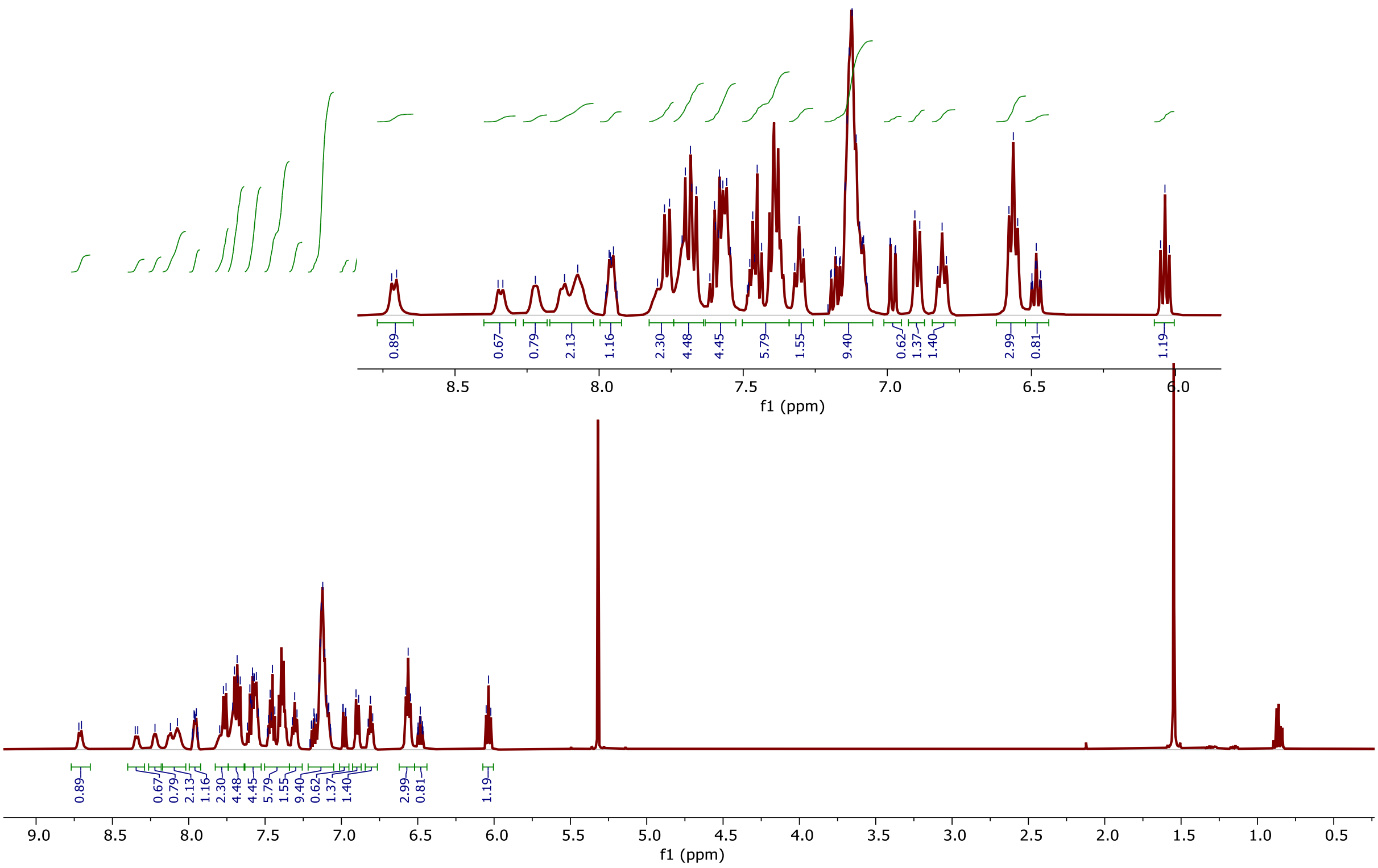

Figure S4. ^{1}H NMR (500 MHz, CD_2Cl_2) spectrum of complex *R*-**2**.

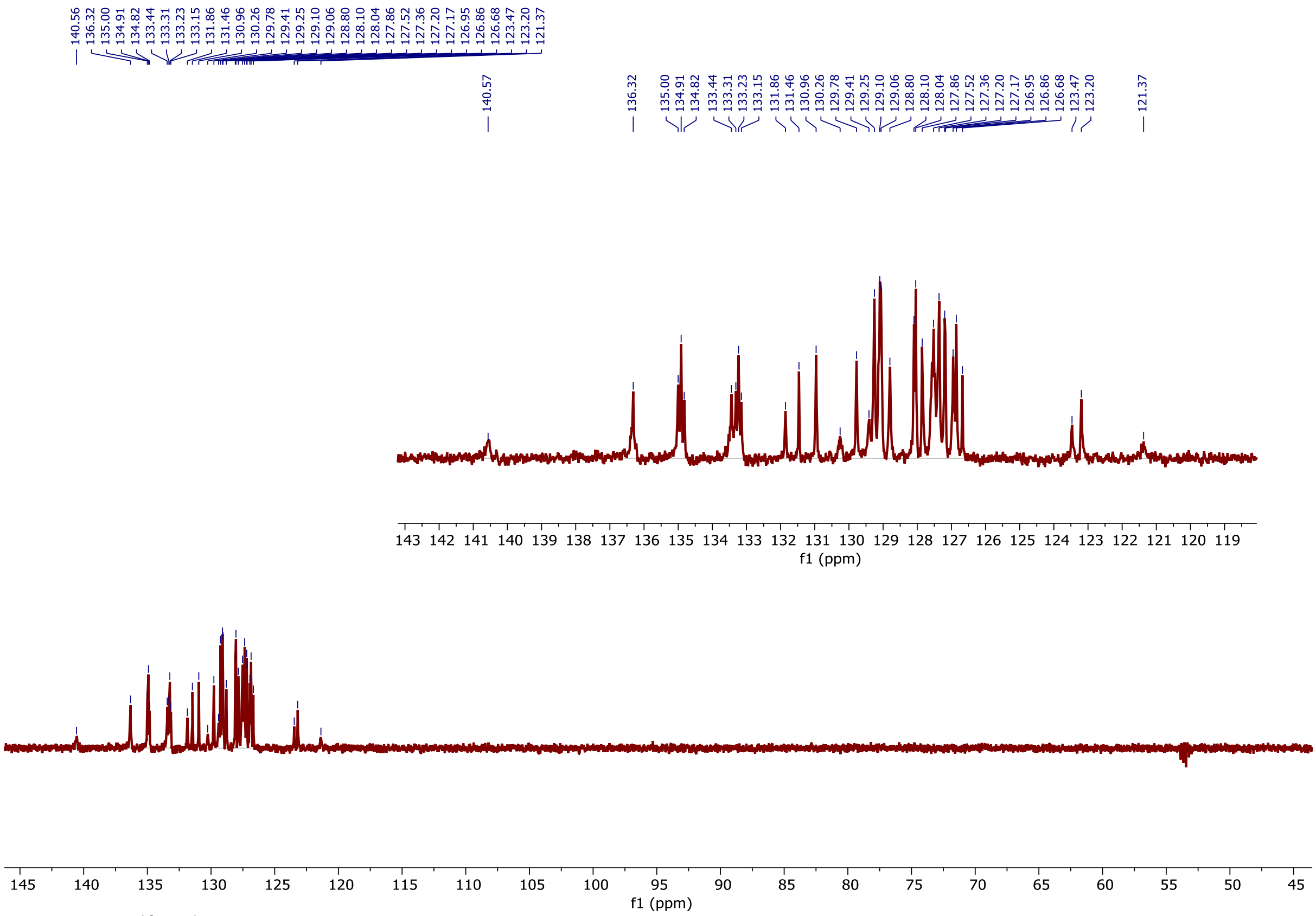

Figure S5. $^{13}C\{^1H\}$ DEPT-135 NMR (126 MHz, CD_2Cl_2) spectrum of complex *R*-**2**.

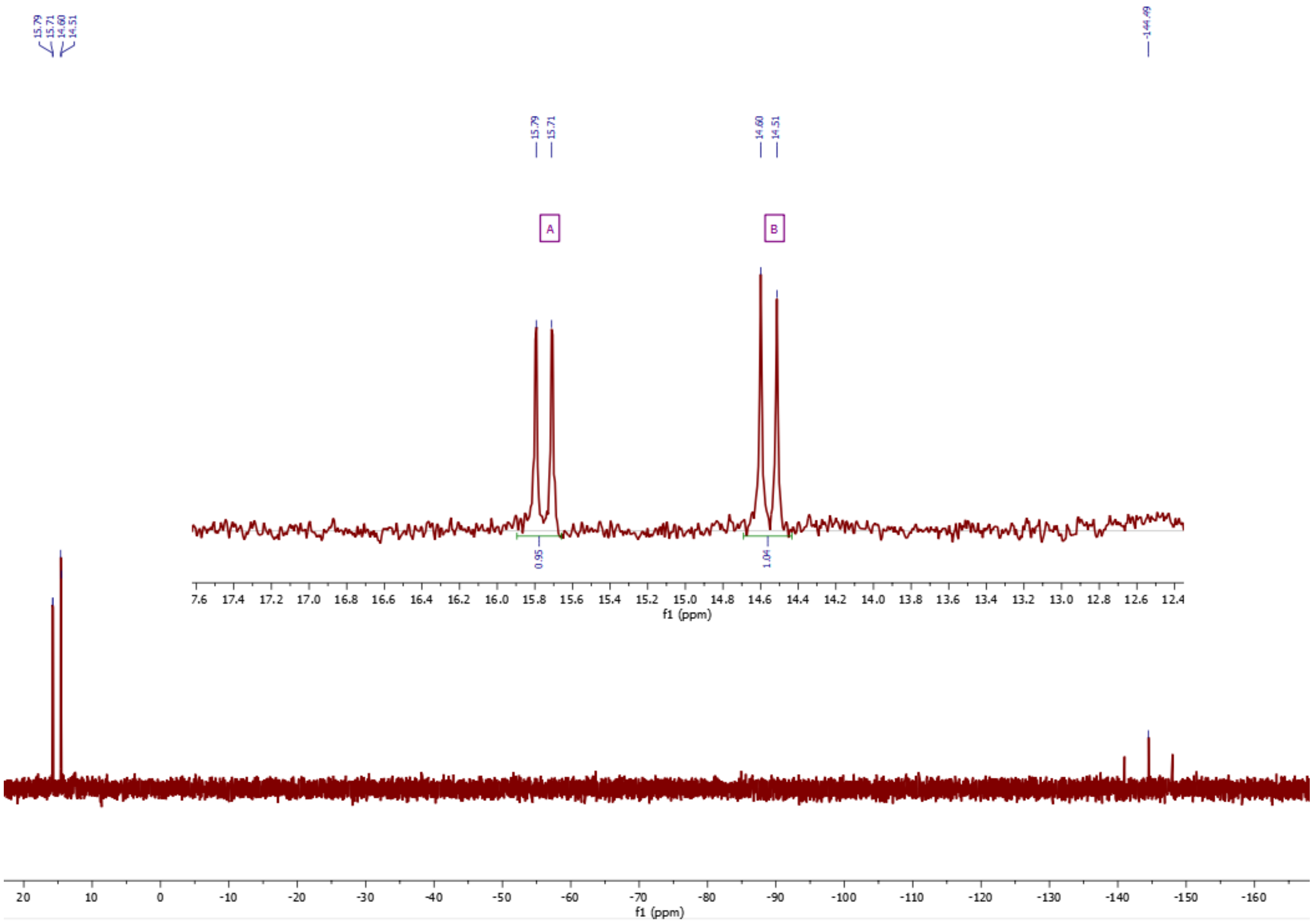

Figure S6. $^{31}P\{^1H\}$ NMR (202 MHz, CD_2Cl_2) spectrum of complex *R*-**2**.

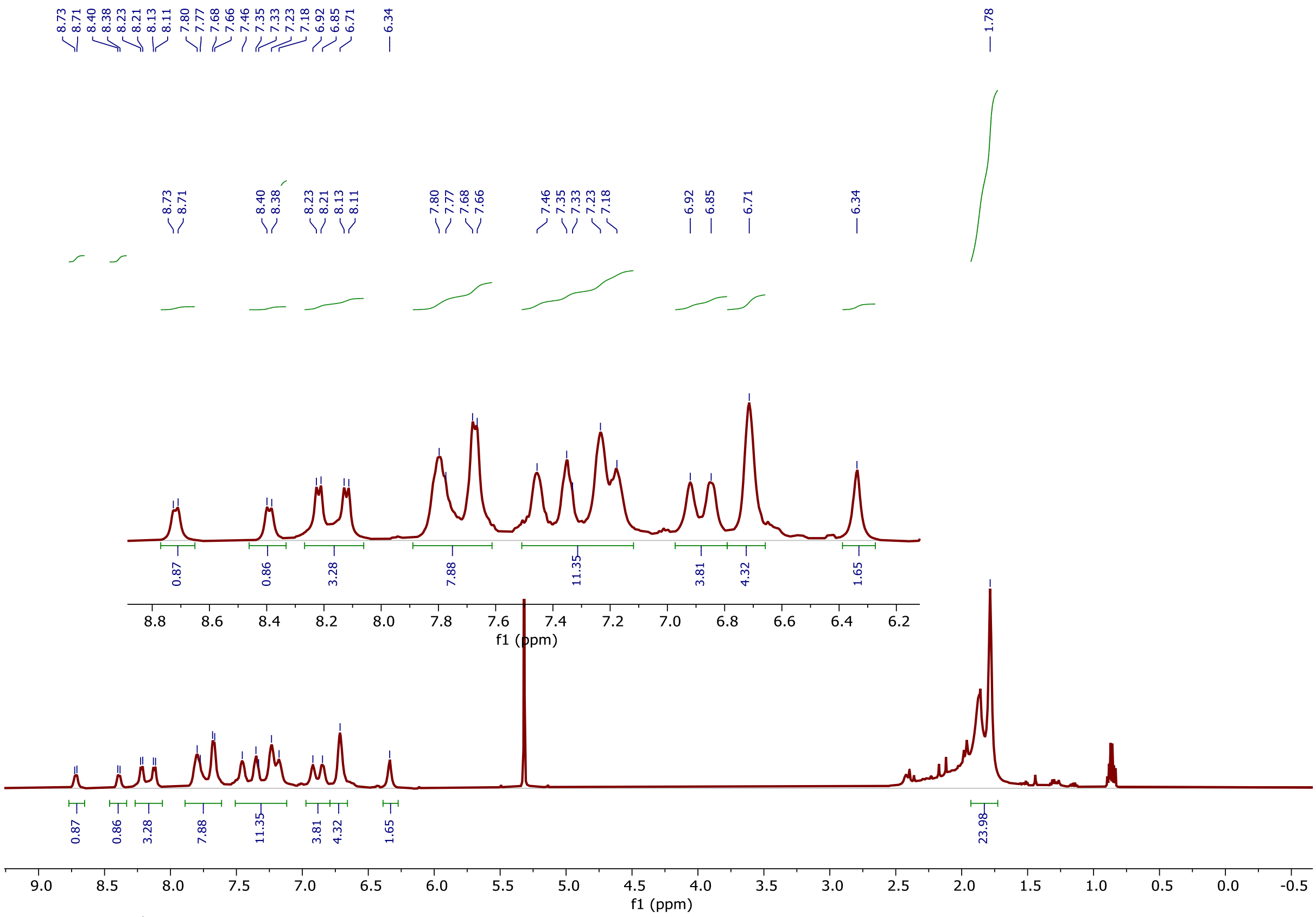

Figure S7. ^{1}H NMR (500 MHz, CD_2Cl_2) spectrum of complex *R*-**3**.

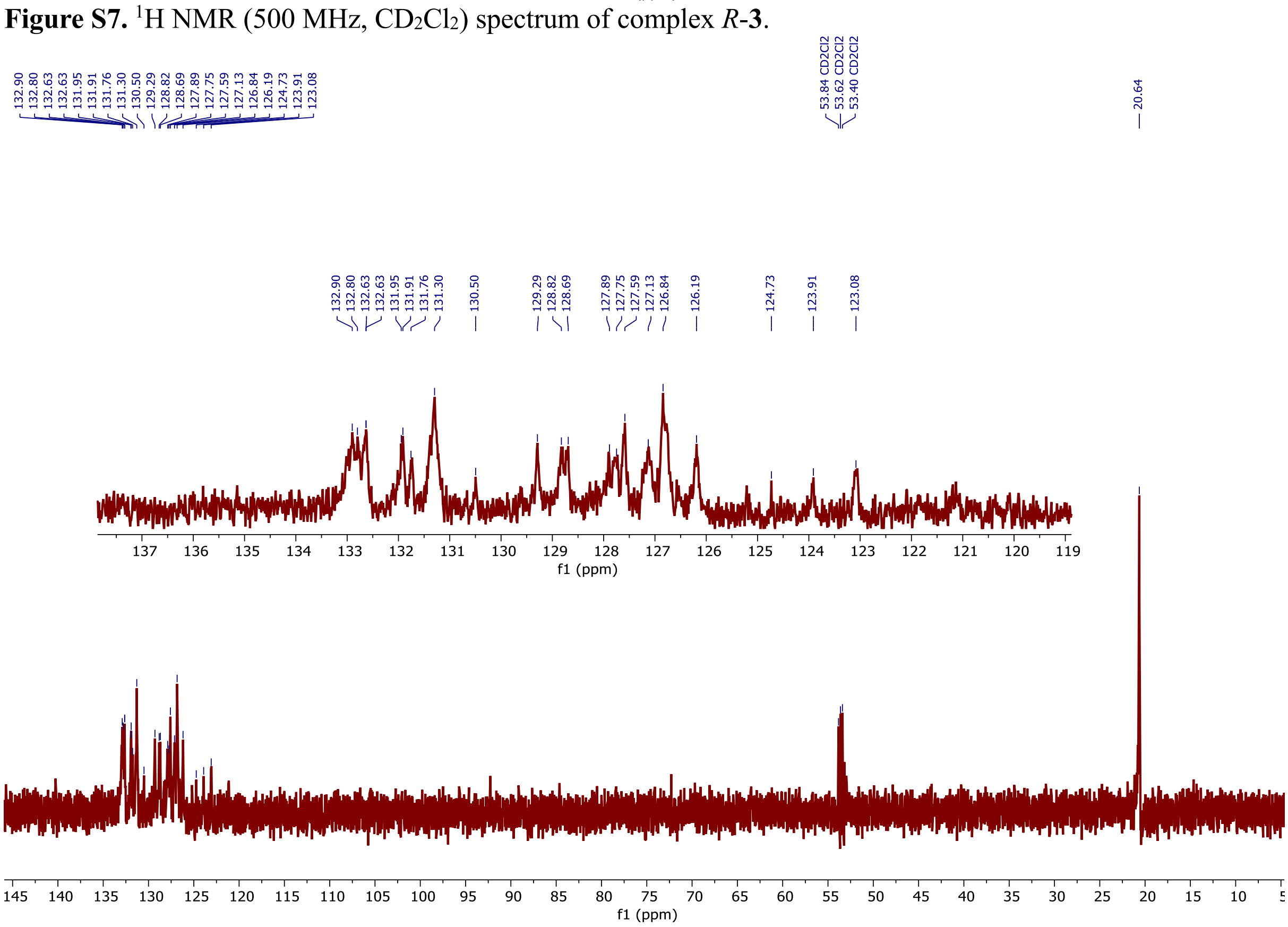

Figure S8. $^{13}C\{^{1}H\}$ DEPT-135 NMR (126 MHz, CD_2Cl_2) spectrum of complex *R*-**3**.

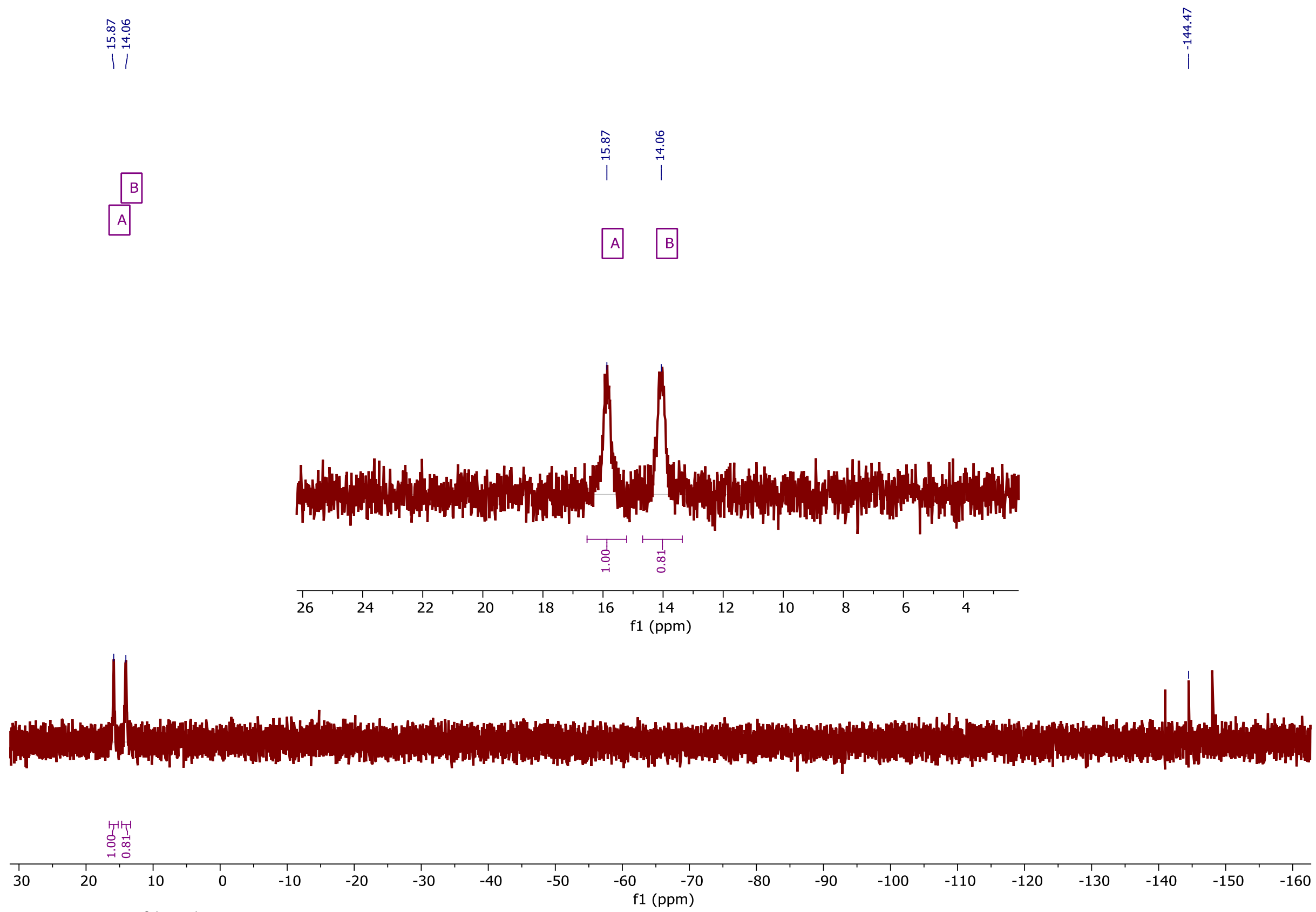

Figure S9. $^{31}P\{^{1}H\}$ NMR (202 MHz, CD_2Cl_2) spectrum of *R*-**3**.

ESI-HR Spectra

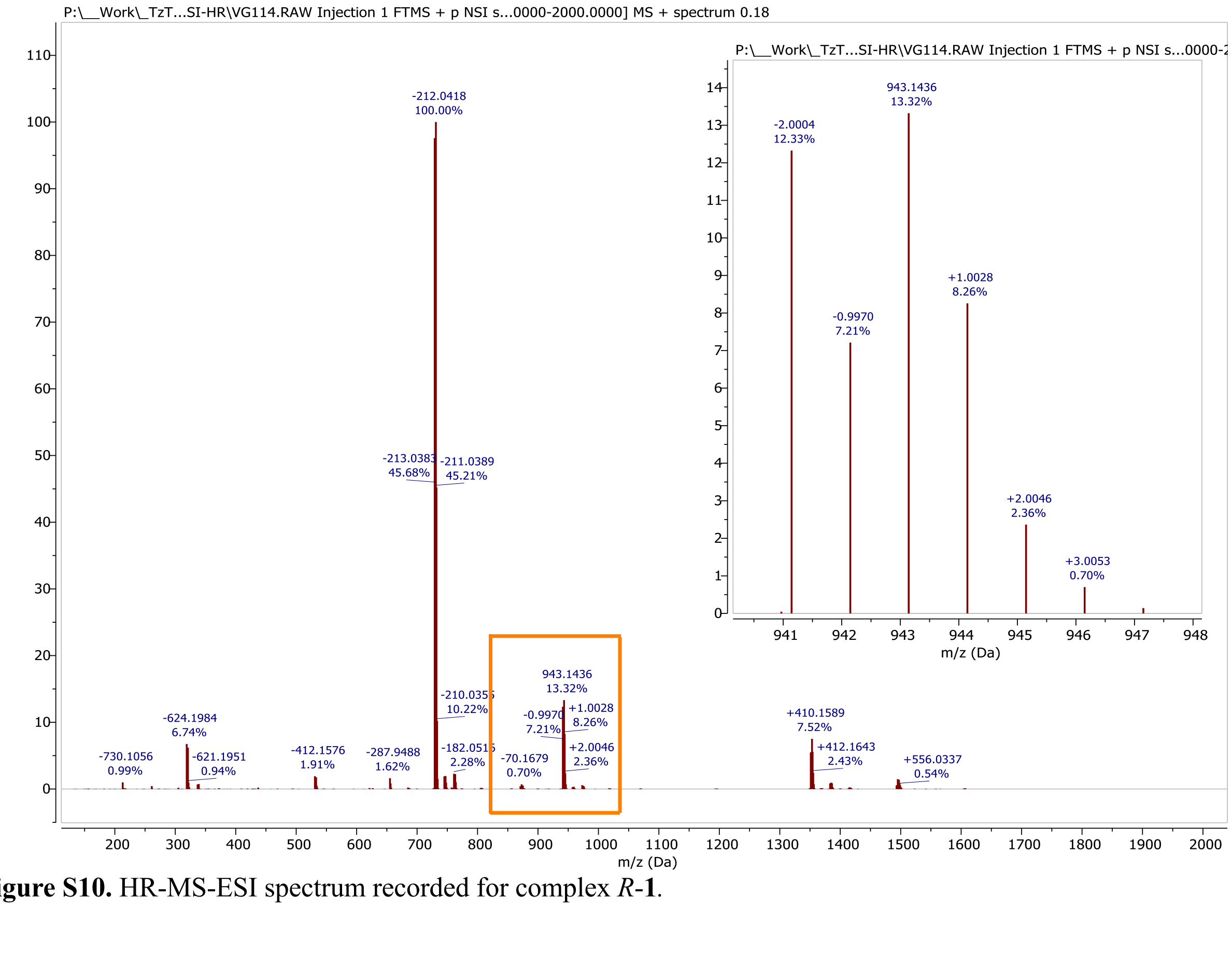

Figure S10. HR-MS-ESI spectrum recorded for complex *R*-**1**.

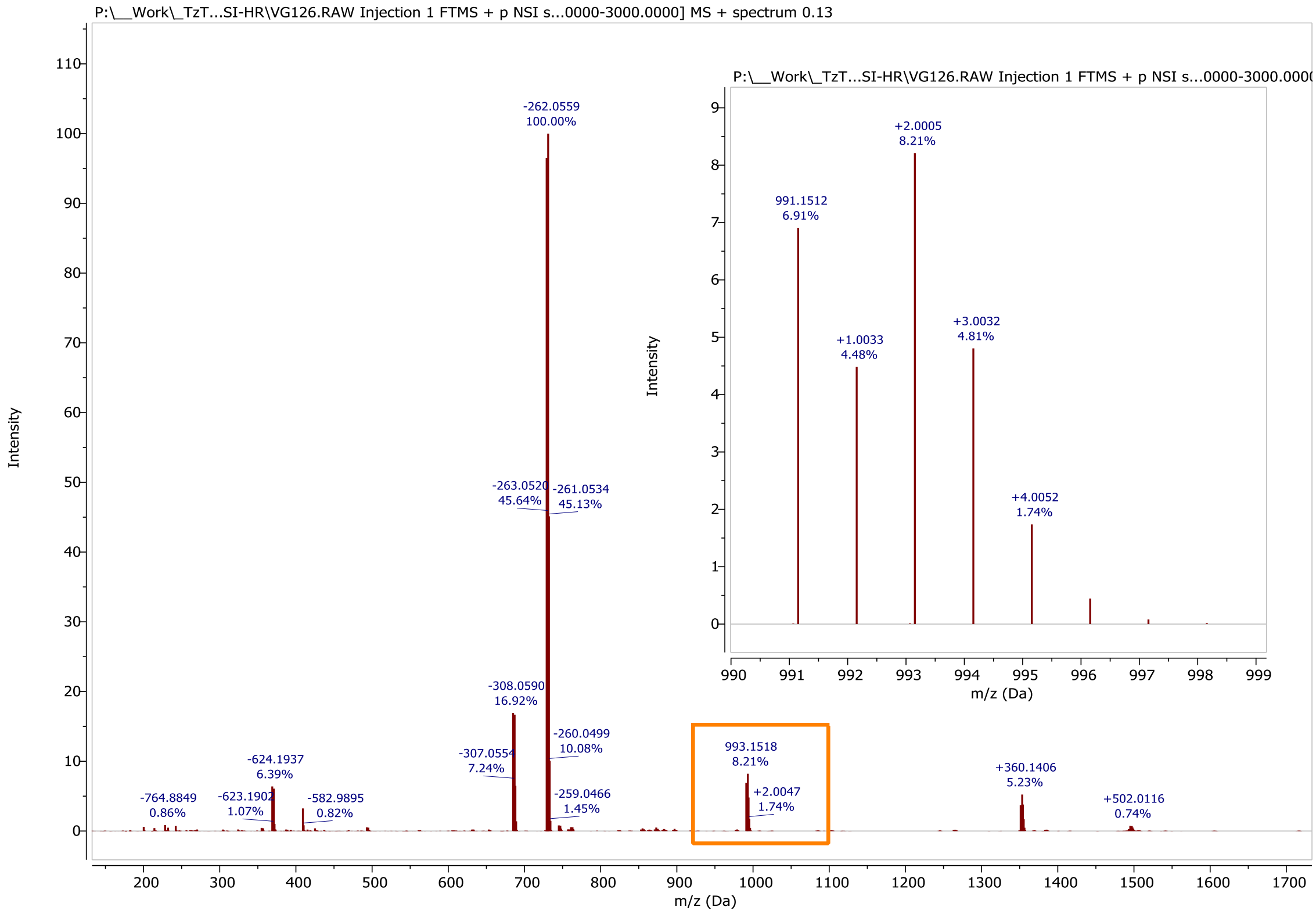

Figure S11. HR-MS-ESI spectrum recorded for complex *R*-**2**.

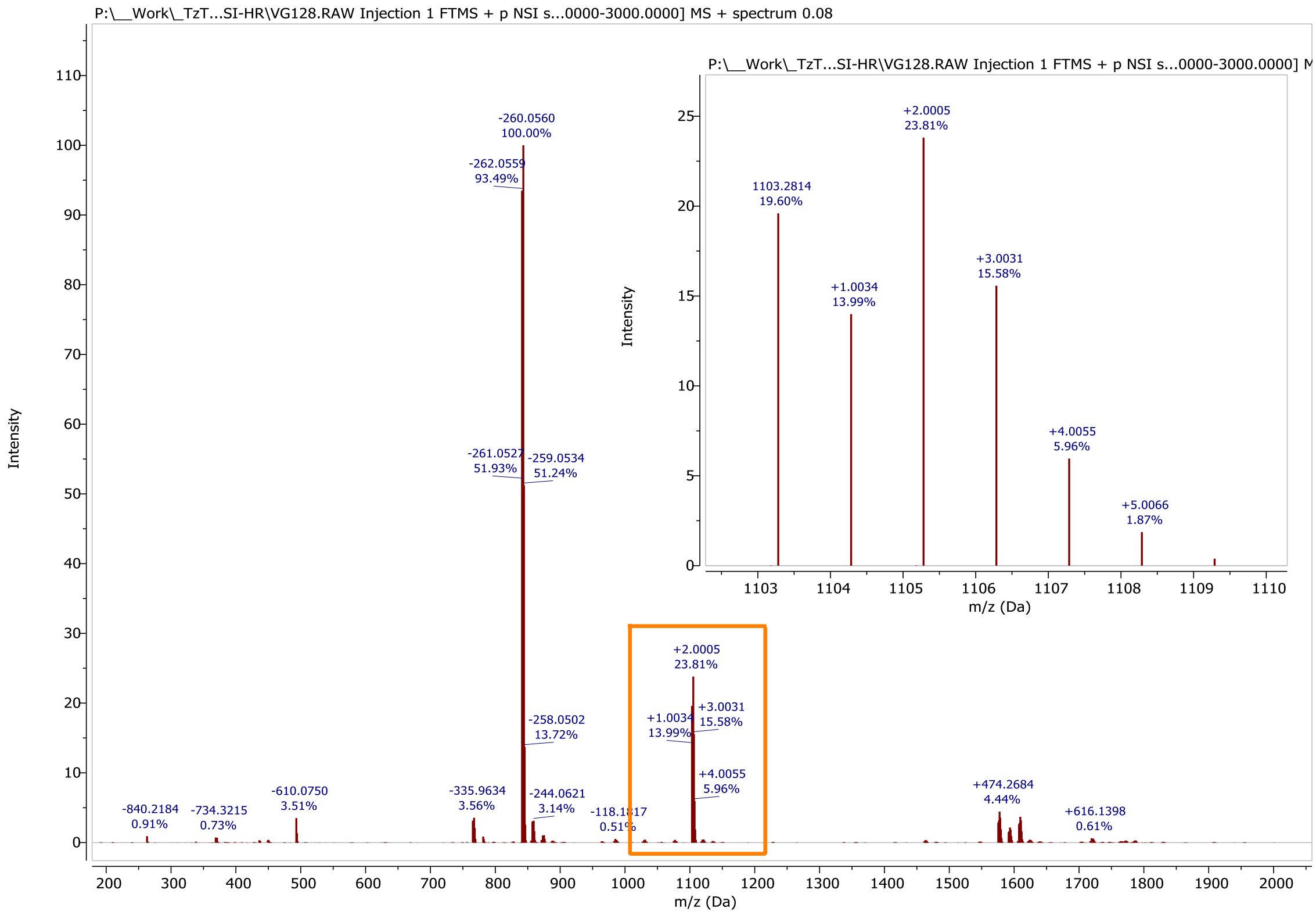

Figure S12. HR-MS-ESI spectrum recorded for complex *R*-**3**.

XRD and SWAXS data

Table S1. Crystal data and structure refinement for complex *S*-**2**.

Identification code	mmvg250429
Empirical formula	C60 H42 Ag F6 N2 P3 S
Formula weight	1137.79
Temperature	120(2) K
Wavelength	0.71073 Å
Crystal system, space group	Orthorhombic, P 21 21 21
Unit cell dimensions	*a* = 11.1943(2) Å *b* = 20.0712(5) Å *c* = 22.4897(6) Å<br>α = 90°, β = 90°, γ = 90°
Volume	5053.1(2) Å^3
Z, Calculated density	4, 1.496 Mg/m^3
Absorption coefficient	0.601 mm^{-1}
F(000)	2312
Crystal size	0.150 × 0.120 × 0.060 mm
Theta range for data collection	2.029 to 28.032°
Limiting indices	$-14 \leq h \leq 14$, $-26 \leq k \leq 26$, $-29 \leq l \leq 29$
Reflections collected / unique	112209 / 12227 [R(int) = 0.0413]
Completeness to θ = 25.242°	99.9%
Absorption correction	Semi-empirical from equivalents
Max. and min. transmission	0.7456 and 0.7062
Refinement method	Full-matrix least-squares on *F*^{2}
Data / restraints / parameters	12227 / 0 / 679
Goodness-of-fit on F^2	1.045
Final R indices [I > 2σ(I)]	R_1 = 0.0263, wR_2 = 0.0608
R indices (all data)	R_1 = 0.0289, wR_2 = 0.0623
Absolute structure parameter	-0.003(5)
Extinction coefficient	n/a
Largest diff. peak and hole	0.430 and -0.629 *e* Å^{-3}

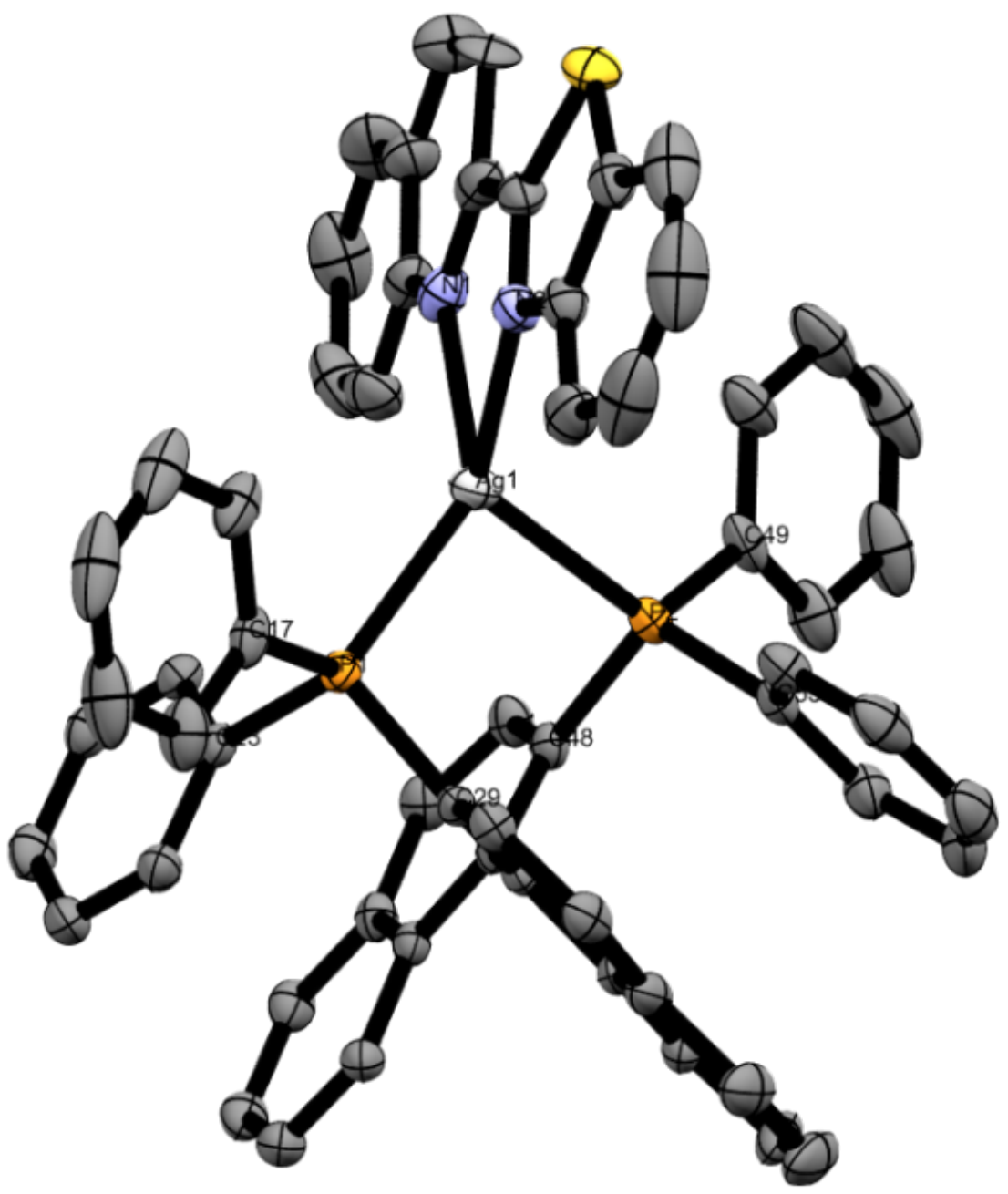

Figure S13. ORTEP diagram of *S*-**2** shown at 50% probability. Hydrogen atoms and the PF_6^- counter anion are omitted for clarity.

Table S2. Crystal data and structure refinement for complex *R*-**3**.

Identification code	emmvg250108
Empirical formula	C68 H58 Ag F6 N2 P3 S
Alternative formula	C68 H58 Ag N2 P2 S, F6 P
Formula weight	1250.00
Temperature	120(2) K
Wavelength	0.71073 Å
Crystal system, space group	Orthorhombic, P 21 21 21
Unit cell dimensions	a = 11.4376(3) Å<br>b = 21.3151(4) Å<br>c = 23.2053(6) Å<br>α = 90°, β = 90°, γ = 90°
Volume	5657.3(2) $Å^3$
Z, Calculated density	4, 1.468 Mg/m^3
Absorption coefficient	0.544 mm^{-1}
F(000)	2568
Crystal size	0.140 × 0.120 × 0.100 mm
Theta range for data collection	1.911 to 27.911°
Limiting indices	$-15 \leq h \leq 14$, $-28 \leq k \leq 27$, $-30 \leq l \leq 30$
Reflections collected / unique	56232 / 13502 [R(int) = 0.0624]
Completeness to θ = 25.242°	99.9%
Absorption correction	Semi-empirical from equivalents
Max. and min. transmission	0.7456 and 0.7062
Refinement method	Full-matrix least-squares on F^2
Data / restraints / parameters	13502 / 33 / 694
Goodness-of-fit on F^2	1.054
Final R indices [I > 2σ(I)]	R_1 = 0.0674, wR_2 = 0.1855
R indices (all data)	R_1 = 0.0829, wR_2 = 0.2000
Absolute structure parameter	-0.003(12)
Extinction coefficient	n/a
Largest diff. peak and hole	1.752 and -1.849 e $Å^{-3}$

Table S3. Crystal data and structure refinement for complex *S*-**3**.

Identification code	emmvg250108-02
Empirical formula	C68 H58 Ag F6 N2 P3 S
Alternative formula	C68 H58 Ag N2 P2 S, F6 P
Formula weight	1250.00
Temperature	120(2) K
Wavelength	0.71073 Å
Crystal system, space group	Orthorhombic, P 21 21 21
Unit cell dimensions	a = 11.5246(4) Å<br>b = 21.3811(6) Å<br>c = 23.1496(8) Å<br>$\alpha = 90°$, $\beta = 90°$, $\gamma = 90°$
Volume	5704.3(3) $Å^3$
Z, Calculated density	4, 1.456 Mg/m^3
Absorption coefficient	0.539 mm^{-1}
F(000)	2568
Crystal size	0.180 × 0.140 × 0.120 mm
Theta range for data collection	2.007 to 27.904°
Limiting indices	$-15 \leq h \leq 15$, $-24 \leq k \leq 28$, $-30 \leq l \leq 30$
Reflections collected / unique	101511 / 13616 [R(int) = 0.0881]
Completeness to θ = 25.242°	99.9%
Absorption correction	Semi-empirical from equivalents
Max. and min. transmission	0.7422 and 0.7057
Refinement method	Full-matrix least-squares on F^2
Data / restraints / parameters	13616 / 6 / 712
Goodness-of-fit on F^2	1.083
Final R indices [I > 2σ(I)]	$R_1 = 0.0550$, $wR_2 = 0.1469$
R indices (all data)	$R_1 = 0.0718$, $wR_2 = 0.1601$
Absolute structure parameter	0.006(10)
Extinction coefficient	n/a
Largest diff. peak and hole	1.056 and -1.441 e $Å^{-3}$

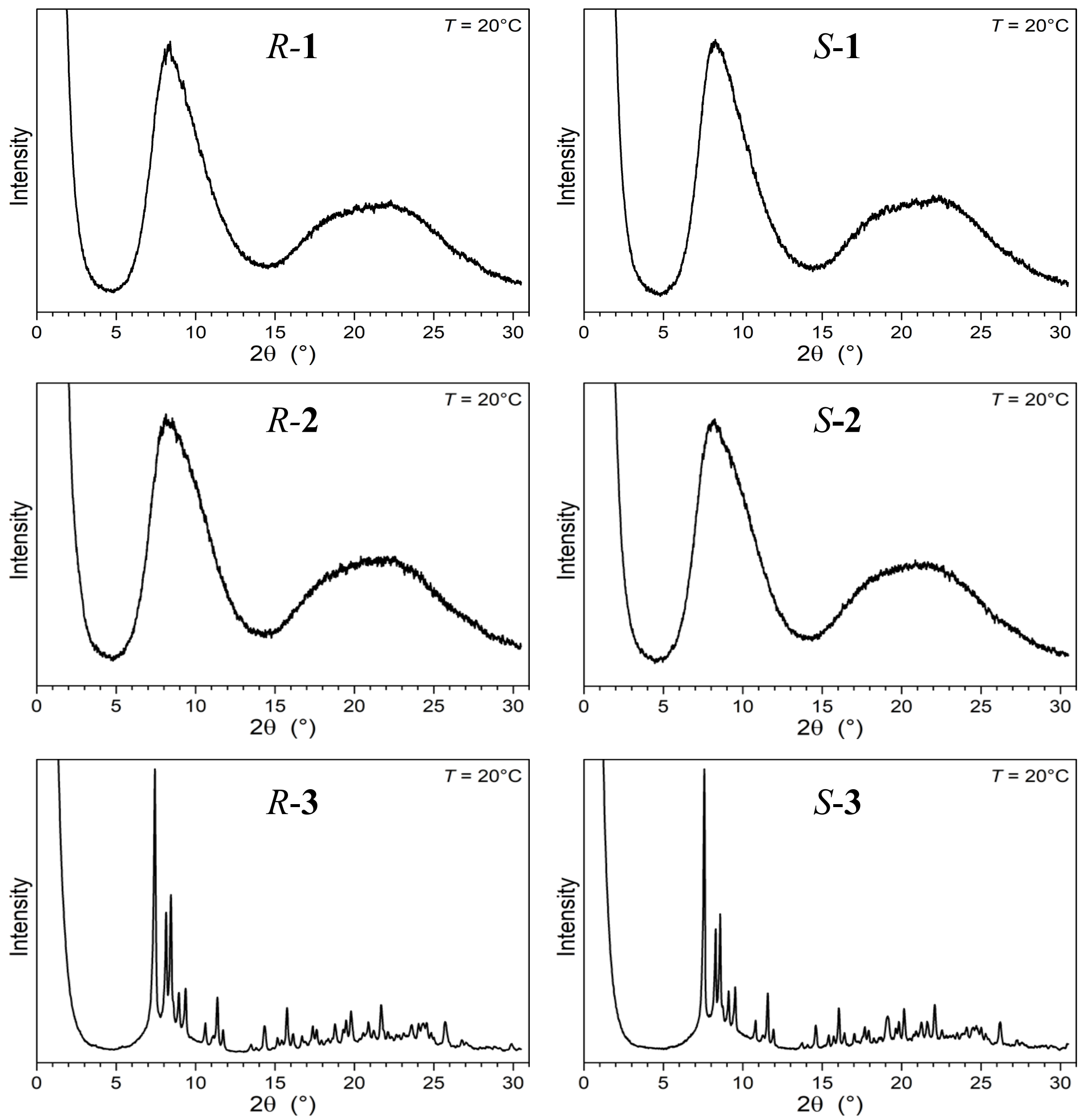

Figure S14. Powder SWAXS of complexes **1**–**3**. Complexes **1**–**2** are amorphous, complexes **3** are semicristalline. The crystalline fractions in *R*-**3** and *S*-**3** have the same structure.

Photophysical measurements

Instrument details

Absorption spectra were recorded using a Perkin Elmer Lambda 950 double-beam UV-VIS spectrophotometer and baseline corrected. Steady-state emission spectra were recorded on a Horiba Jobin−Yvon IBH FL-322 Fluorolog 3 spectrometer equipped with a 450 W xenon arc lamp, double-grating excitation, and emission monochromators (2.1 nm mm^{-1} of dispersion; 1200 grooves mm^{-1}) and a Hamamatsu R13456 red sensitive Peltier-cooled PMT detector. Emission and excitation spectra were corrected for source intensity (lamp and grating) and emission spectral response (detector and grating) by standard correction curves. Time-resolved measurements were performed using either the Time-Correlated Single-Photon Counting (TCSPC) or the Multi-Channel Scaling (MCS) electronics option of the TimeHarp 260 board installed on a PicoQuant FluoTime 300 fluorimeter (PicoQuant GmbH, Germany), equipped with a PDL 820 laser pulse driver. A pulsed laser diode LDH-P-C-375 (λ = 375 nm, pulse full width at half maximum <50 ps, repetition rate 200 kHz–40 MHz) was used to excite the sample and mounted directly on the sample chamber at 90°. The photons were collected by a PMA Hybrid-07 single photon counting detector. The data were acquired by using the commercially available software EasyTau II (PicoQuant GmbH, Germany), while data analysis was performed using the built-in software FluoFit (PicoQuant GmbH, Germany). All the solvents were spectrophotometric grade. Dearated samples were prepared by the freeze–pump–thaw technique by using a home-made quartz cuvette equipped with a Rotaflo stopcock. Luminescence quantum yields were measured in optically dilute solutions (optical density <0.15 at the excitation wavelength) and compared to reference emitter by following the method of Demas and Crosby.[1] The $Ru(bpy)_3Cl_2$ complex in air-equilibrated water solution at room temperature was used as reference (PLQY = 0.04).[2] Solid state PLQY values were recorded at a fixed excitation wavelength by using a Hamamatsu Photonics absolute PLQY measurements system Quantaurus QY equipped with CW Xenon light source (150 W), monochromator, integrating sphere, C7473 photonics multi-channel analyzer and employing the commercially available U6039-05 PLQY measurement software (Hamamatsu Photonics Ltd., Shizuoka, Japan). All measurements were repeated four times at the excitation wavelength λ_{exc} = 360 nm, unless otherwise stated.

Methods

For time resolved measurements, data fitting was performed by employing the maximum likelihood estimation (MLE) methods and the quality of the fit was assessed by

inspection of the reduced χ^2 function and of the weighted residuals. For multi-exponential decays, the intensity, namely I*(t)*, has been assumed to decay as the sum of individual single exponential decays (Eqn. 1):

$$I(t) = \sum_{i=1}^{n} \alpha_i exp\left(-\frac{t}{\tau_i}\right) \qquad \text{eqn. 1}$$

where τ_i are the decay times and α_i are the amplitude of the component at $t = 0$. In the tables, the percentages to the pre-exponential factors, α_i, are listed upon normalization.

Intensity average lifetimes were calculated by using the following equation (Eqn. 2):[3]

$$\bar{\tau} = \frac{a_1\tau_1^2 + a_2\tau_2^2}{a_1\tau_1 + a_2\tau_2} \qquad \text{eqn. 2}$$

Supplementary photophysical data

Table S4. Photophysical data recorded for complexes *S*-**1**, *S*-**2** and *S*-**3** in dilute degassed CH_2Cl_2 at room temperature. *sh* denotes a shoulder, *dec*. denotes photodecomposition.

complex	$\lambda_{max}, abs\ (\varepsilon)\ [nm, (10^3 \cdot M^{-1} \cdot cm^{-1})]$	$\lambda_{em}\ [nm]$
S-**1**	278*sh* (28.13), 293*sh* (23.38), 311 (22.68), 325*sh* (21.74), 341 (15.55), 379*sh* (1.99)	610 *(dec.)*
S-**2**	283*sh* (31.11), 293*sh* (28.15), 349 (21.92), 366 (17.16) *405sh,br* (2.10)	670
S-**3**	283*sh* (29.26), 321*sh* (18.25), 335*sh* (19.43), 349 (21.45), 366 (15.38) *414sh* (1.78)	685 *(dec.)*

Table S5. Photophysical data recorded for complexes *S*-**1**, *S*-**2** and *S*-**3** as neat powders under vacuum, in CH_2Cl_2 glassy matrix at 77 K, and in PMMA 120k polymer-doped spin-coated thin films at 25 wt.% under vacuum. *sh* denotes a shoulder, *br* denotes a broad signal.

cmpd	$\lambda_{em}\ [nm]$			$PLQY$ (%)		τ_{obs} [μs] (% A_{rel})			$\bar{\tau}$		
	powder	77 K (CH_2Cl_2)	25 wt.% in PMMA	*powder*	25 wt.% in PMMA	*powder*	77 K (CH_2Cl_2)	25 wt.% in PMMA	*powder*	77 K (CH_2Cl_2)	25 wt.% in PMMA
S-1	**620**	515, **555**, 600, 650*sh*	**590**	2	6	15.2 ms (12%) 3.93 ms (40%) 1.33 ms (47%)	15.4 ms (25%) 7.39 ms (53%) 1.49 ms (21%)	5.57 ms (13%) 1.59 ms (38%) 0.435 ms (49%)	8.71 ms	11.0 ms	3.28 ms
S-2	**647**	525, 538*sh*, **568**, 585*sh*, 618, 674	**615**	5	16	9.44 ms (7%) 2.42 ms (41%) 0.80 ms (52%)	4.49 ms (4%) 1.60 ms (54%) 0.540 ms (43%)	1.72 ms (16%) 0.635 ms (44%) 0.216 ms (40%)	4.30 ms	1.79 ms	1.05 ms
S-3	**645**	521, 535*sh*, **566**, 582*sh*, 615, 675	**620**	30	45	12.4 ms (10%) 3.26 ms (46%) 0.75 ms (43%)	3.86 ms (6%) 1.38 ms (54%) 0.453 ms (40%)	2.01 ms (10%) 0.656 ms (43%) 0.217 ms (47%)	6.72 ms	1.70 ms	1.04 ms

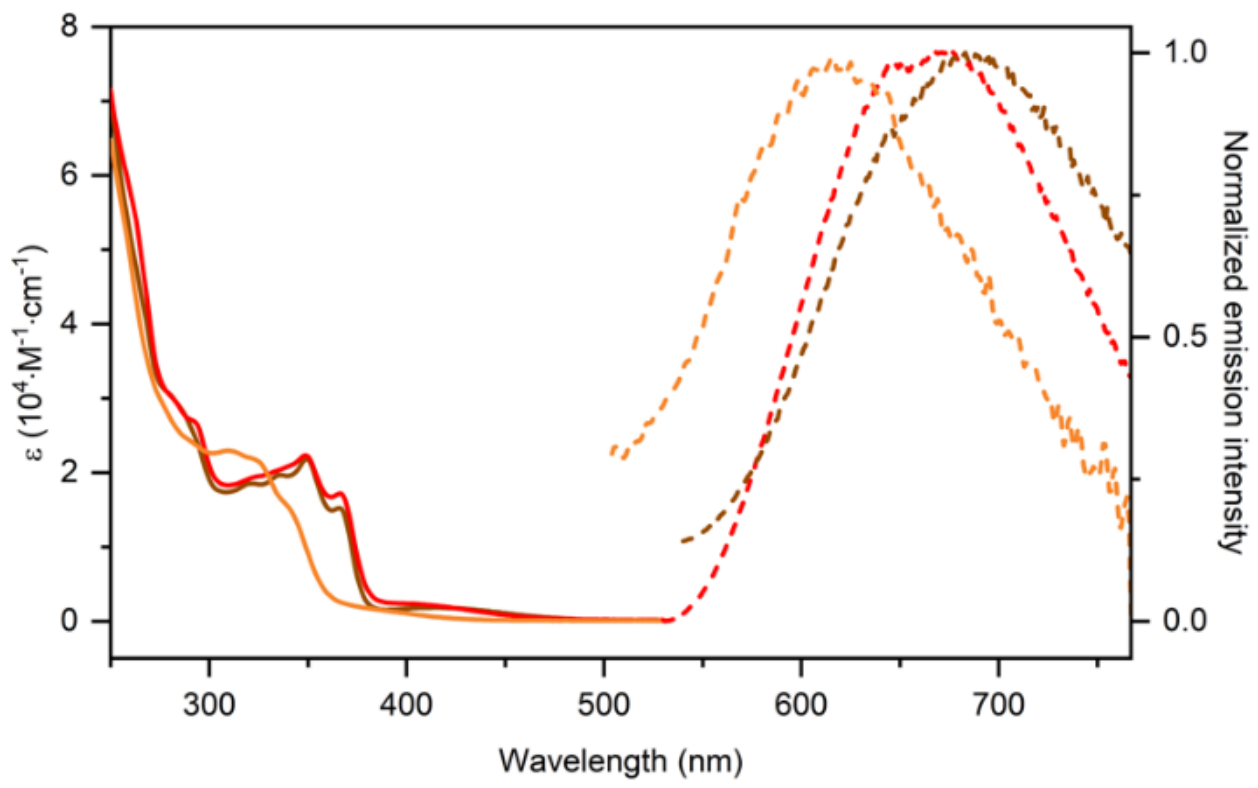

Figure S15. Electronic absorption (solid) and emission (dashed) spectra of complexes *S*-**1** (orange), *S*-**2** (red), *S*-**3** (brown) in dilute ($2 \cdot 10^{-5}$ M) degassed CH_2Cl_2 at room temperature upon excitation at λ_{exc} = 350, 360, and 370 nm respectively.

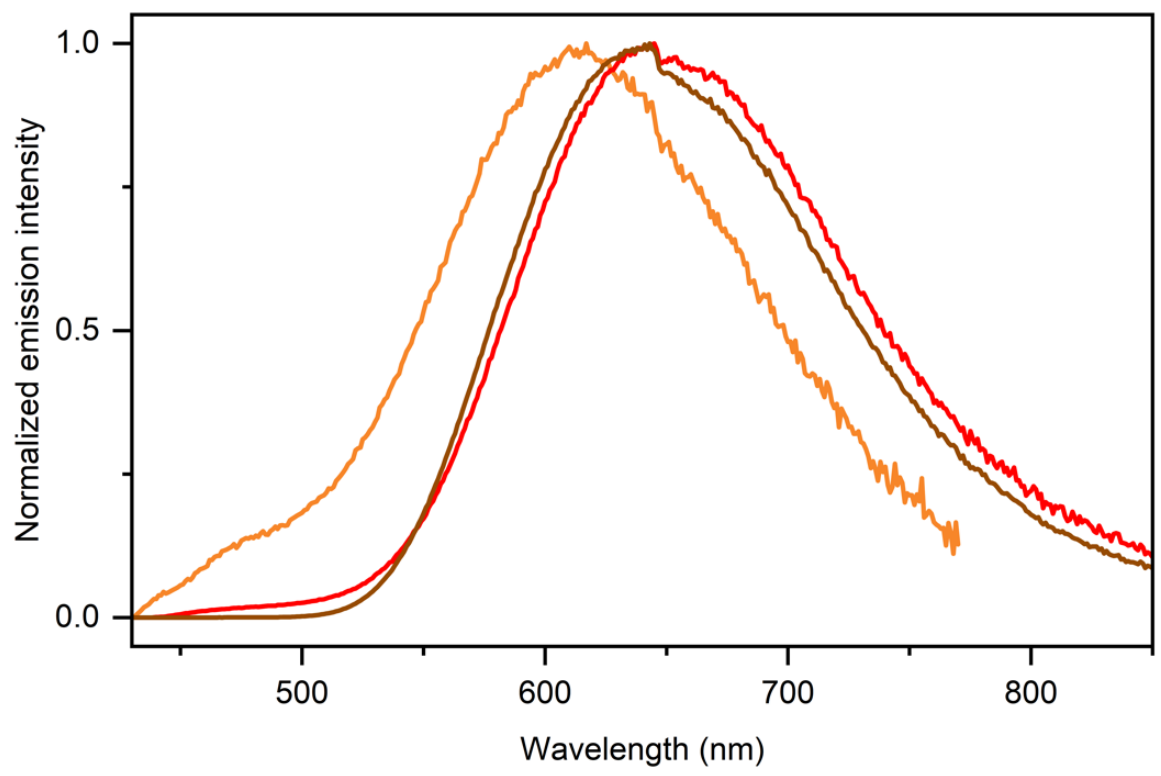

Figure S16. Emission spectra of complexes *S*-**1** (orange), *S*-**2** (red), and *S*-**3** (brown) in the solid state as neat powders under oxygen-free atmosphere at room temperature upon excitation at λ_{exc} = 400 nm.

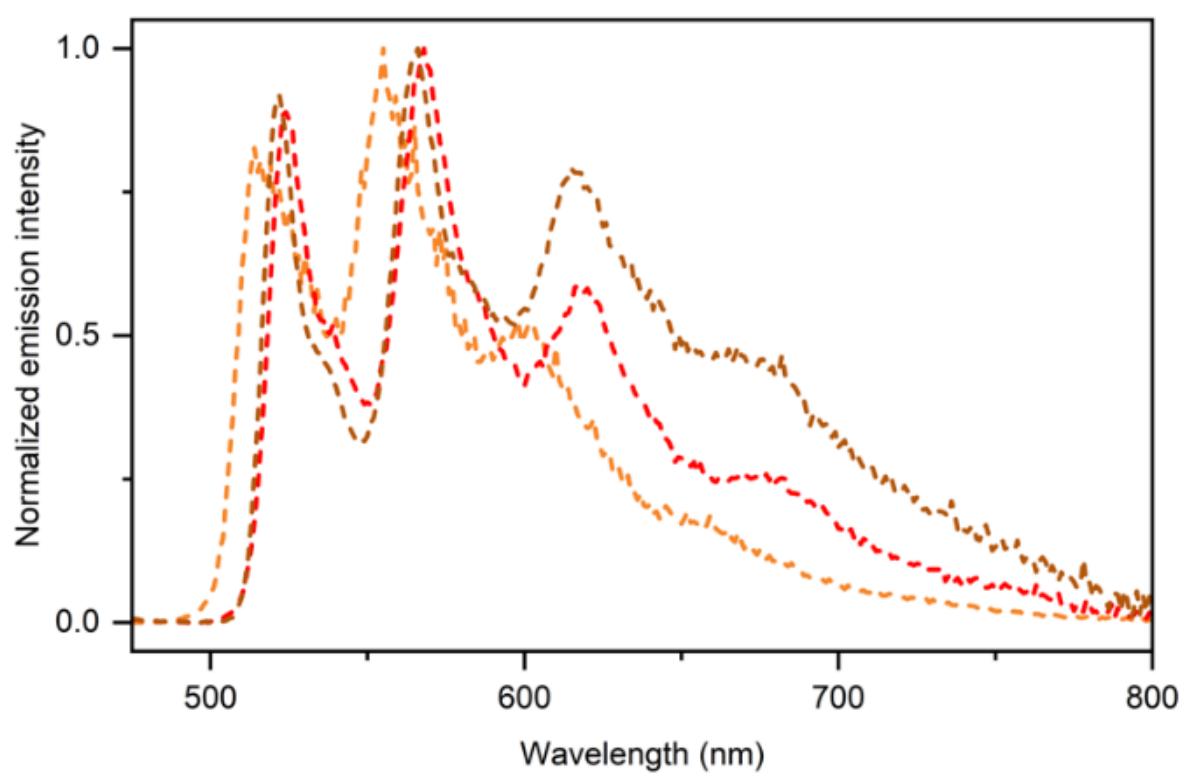

Figure S17. Emission spectra of complexes *S*-**1** (orange), *S*-**2** (red), and *S*-**3** (brown) in CH_2Cl_2 glassy matrix at 77 K upon excitation at λ_{exc} = 400 nm.

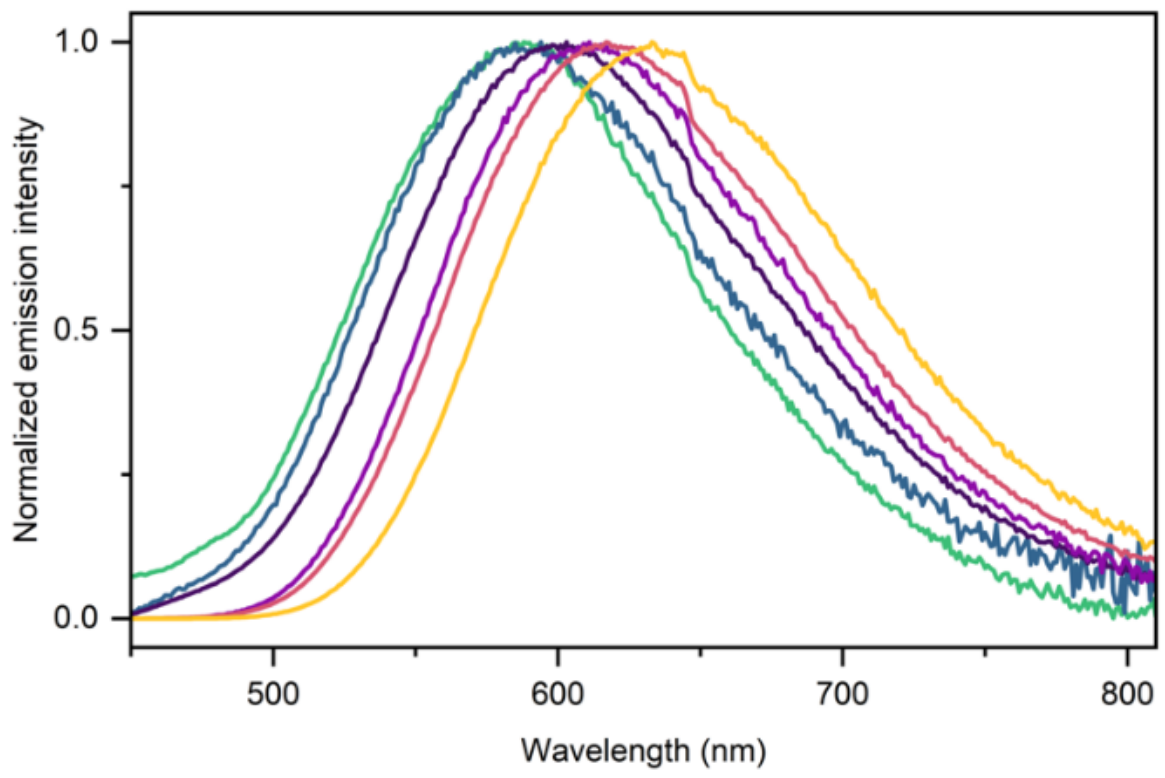

Figure S18 Emission spectra of complexes *R*-**1** and *R*-**3** in air-equilibrated PMMA 120k polymer-doped spin-coated thin films. *R*-**1** at 5 (teal), 25 (blue), and 50 (violet) wt.% concentration and *R*-**3** at 5 (purple), 25 (orange), and 50 (yellow) wt.% concentration upon excitation at λ_{exc} = 420 nm.

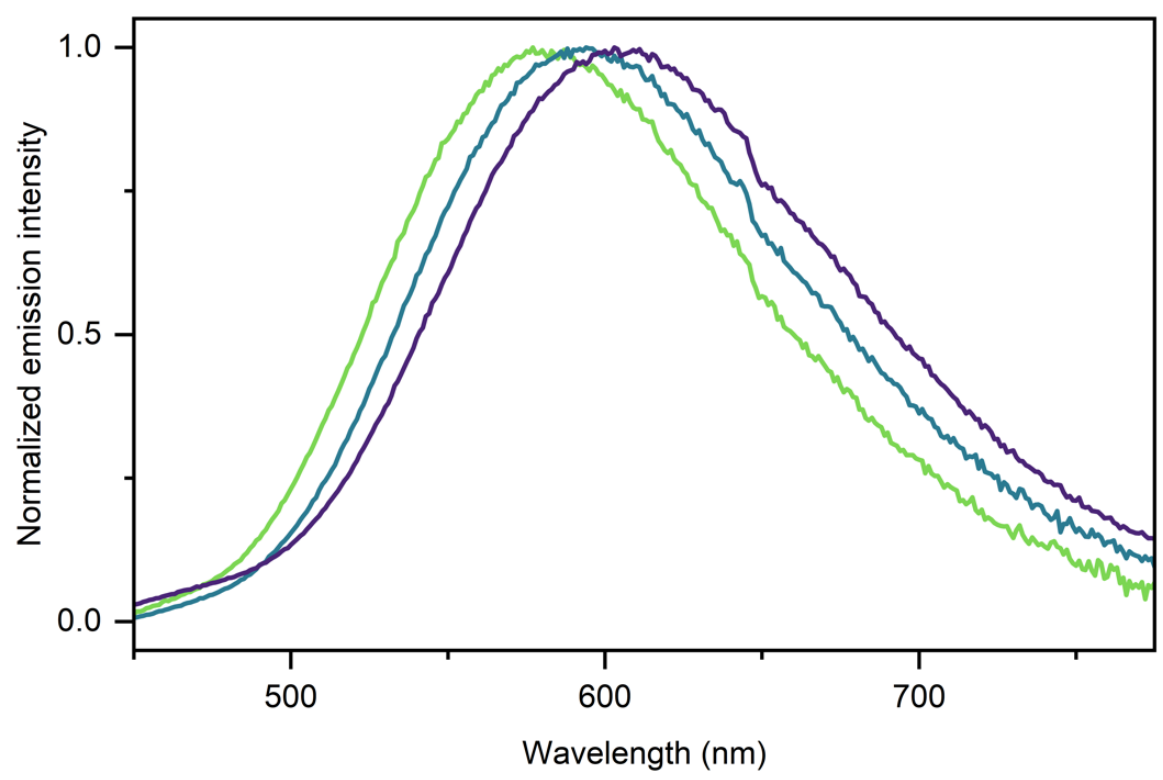

Figure S19. Emission spectra of complexes *S*-**1** in air-equilibrated PMMA 120k polymer-doped spin-coated thin films at 5 (green), 25 (teal), and 50 (blue) wt.% concentration upon excitation at λ_{exc} = 420 nm.

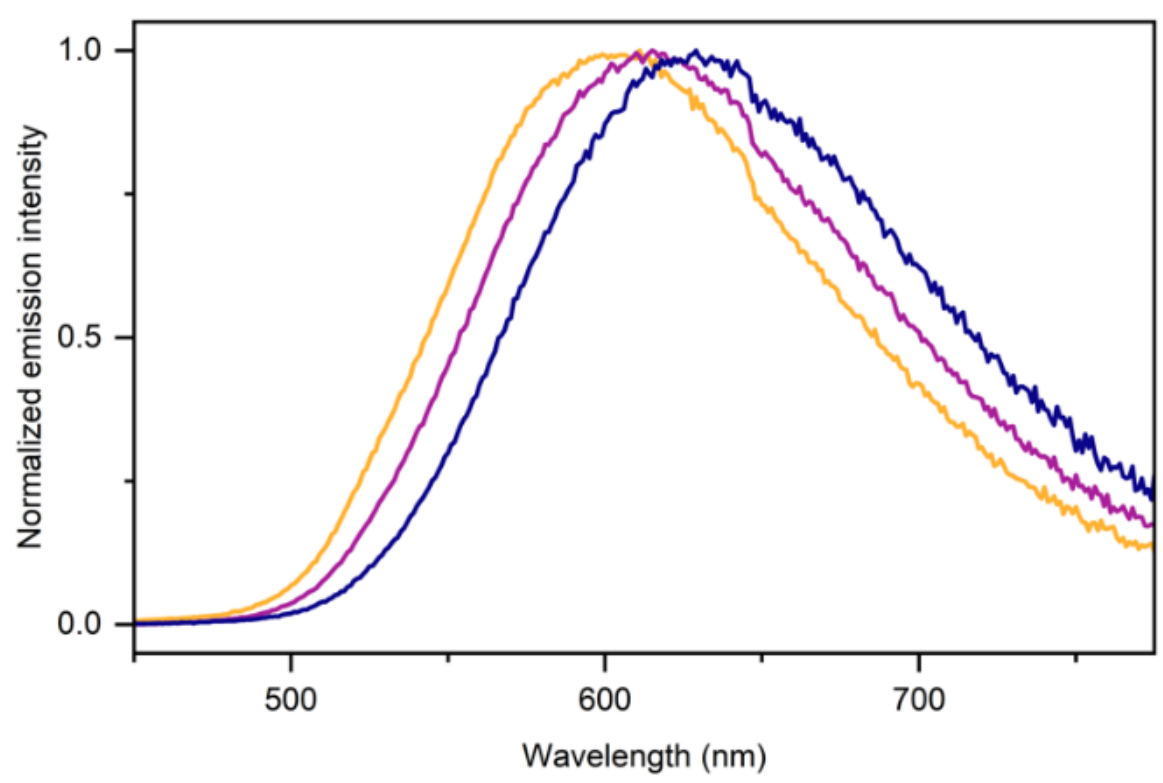

Figure S20. Emission spectra of complexes *S*-**2** in air-equilibrated PMMA 120k polymer-doped spin-coated thin films at 5 (orange), 25 (violet), and 50 (blue) wt.% concentration upon excitation at λ_{exc} = 420 nm.

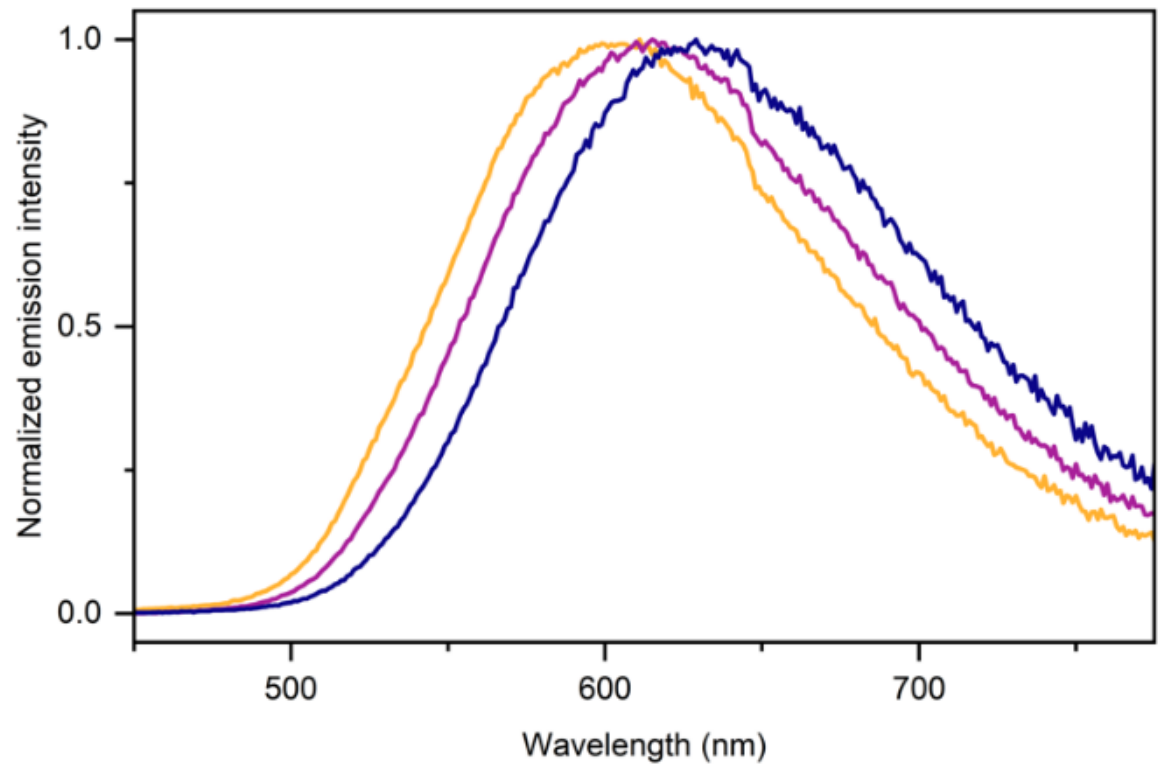

Figure S21. Emission spectra of complexes *S*-**3** in air-equilibrated PMMA 120k polymer-doped spin-coated thin films at 5 (orange), 25 (violet), and 50 (blue) wt.% concentration upon excitation at λ_{exc} = 420 nm.

Table S6. Photophysical data for *R*-**1**, *R*-**2** and *R*-**3** as air-equilibrated polymer-doped spin-coated thin films.

Cmpd	Doping wt.%	λ_{em} [nm]	PLQY (%)<br>CIE (x, y)	τ_{obs} [μs]<br>(% A_{rel})	$\bar{\tau}$
R-**1**	**5**	**585**	5<br>(0.33, 0.36)	153 μs (13%)<br>44.3 μs (39%)<br>10.5 μs (49%)	90.7 μs
	**25**	**585**	5<br>(0.37, 0.39)	207 μs (14%)<br>56.2 μs (42%)<br>14.9 μs (44%)	125 μs
	**50**	**600**	4<br>(0.40, 0.39)	160 μs (2%)<br>48.1 μs (13%)<br>16.0 μs (42%)<br>5.59 μs (43%)	50.0 μs
R-**2**	**5**	**610**	17<br>(0.47, 0.43)	208 μs (5%)<br>62.4 μs (18%)<br>19.7 μs (39%)<br>5.90 μs (38%)	99.5 μs
	**25**	**615**	16<br>(0.50, 0.42)	192 μs (3%)<br>53.0 μs (13%)<br>15.5 μs (39%)<br>4.52 μs (45%)	73.4 μs
	**50**	**620**	12<br>(0.51, 0.39)	85.7 μs (1%)<br>26.2 μs (10%)<br>8.48 μs (41%)<br>2.85 μs (47%)	22.7 μs
R-**3**	**5**	**610**	26<br>(0.46, 0.41)	101 μs (2%)<br>29.6 μs (11%)<br>8.63 μs (37%)<br>2.68 μs (50%)	33.9 μs
	**25**	**620**	31<br>(0.52, 0.42)	87.3 μs (1%)<br>24.5 μs (9%)<br>7.25 μs (36%)<br>2.34 μs (54%)	24.3 μs
	**50**	**630**	21<br>(0.53, 0.40)	28.4 μs (1%)<br>5.76 μs (16%)<br>1.52 μs (82%)	7.39 μs

Table S7. Photophysical data for *S*-**1**, *S*-**2** and *S*-**3** as air-equilibrated polymer-doped spin-coated thin films.

	Doping wt.%	λ_{em} $[nm]$	$PLQY$ (%)<br>CIE (x, y)	τ_{obs} [µs]<br>(%A_{rel})	$\bar{\tau}$
S-**1**	**5**	**578**	5<br>(0.33, 0.35)	155 µs (9%)<br>46.3 µs (38%)<br>10.7 µs (53%)	82.6 µs
	**25**	**591**	6<br>(0.40, 0.40)	218 µs (8%)<br>54.2 µs (37%)<br>13.9 µs (55%)	109 µs
	**50**	**607**	3<br>(0.39, 0.37)	177 µs (1%)<br>42.3 µs (8%)<br>11.6 µs (39%)<br>3.42 µs (52%)	43.3 µs
S-**2**	**5**	**602**	15<br>(0.47, 0.43)	184 µs (3%)<br>57.8 µs (16%)<br>17.2 µs (40%)<br>4.89 µs (42%)	68.8 µs
	**25**	**615**	13<br>(0.46, 0.39)	172 µs (2%)<br>54.2 µs (14%)<br>17.4 µs (40%)<br>5.39 µs (44%)	57.3 µs
	**50**	**630**	10<br>(0.45, 0.36)	137 µs (2%)<br>42.9 µs (12%)<br>13.3 µs (44%)<br>4.14 µs (42%)	41.1 µs
S-**3**	**5**	**609**	33<br>(0.51, 0.44)	149 µs (2%)<br>51.7 µs (12%)<br>16.9 µs (37%)<br>5.03 µs (50%)	46.2 µs
	**25**	**623**	33<br>(0.54, 0.43)	98.8 µs (1%)<br>25.3 µs (15%)<br>5.62 µs (83%)	26.6 µs
	**50**	**636**	25<br>(0.56, 0.41)	10.3 µs (2%)<br>2.97 µs (18%)<br>1.27 µs (80%)	2.85 µs

Chiroptical measurements

Instrument details and methods

Electronic circular dichroism (ECD) spectra in solution and as polymer-doped spin-coated thin films on fused silica were recorded at room temperature using a Jasco J-1500 spectropolarimeter with parameters as follows: scan speed – 100 nm/min , bandwidth – 1 nm, data interval – 0.2 nm, and integration time – 1 sec, for solutions, and scan speed – 200 nm/min, bandwidth – 1 sec, data interval – 1 nm, and integration time – 1 sec, for thin films. ECD signal is obtained as ellipticity (θ) and reported in units of absorbance (ΔA) for solid samples or molar extinction (Δε) for samples in solution according to eqn. 3 and 4:

$$\Delta Abs = \frac{\theta}{32980} \qquad \textit{eqn. 3}$$

$$\Delta\varepsilon = \frac{\theta}{32980 \cdot b \cdot c} \qquad \textit{eqn. 4}$$

Where b and c are the molar concentration of the sample and the cell path length in cm, respectively. The dissymmetry factor g_{abs} at a specific absorption wavelength can be calculated according to eqn. 5:

$$g_{abs}(\lambda) = \frac{ECD}{Abs} = \frac{\theta}{32980 \cdot Abs} \qquad \textit{eqn. 5}$$

Circularly polarized luminescence (CPL) spectra were recorded with a home-made spectrofluoropolarimeter[4] using a 365 nm LED excitation source.

Thermogravimetric measurements

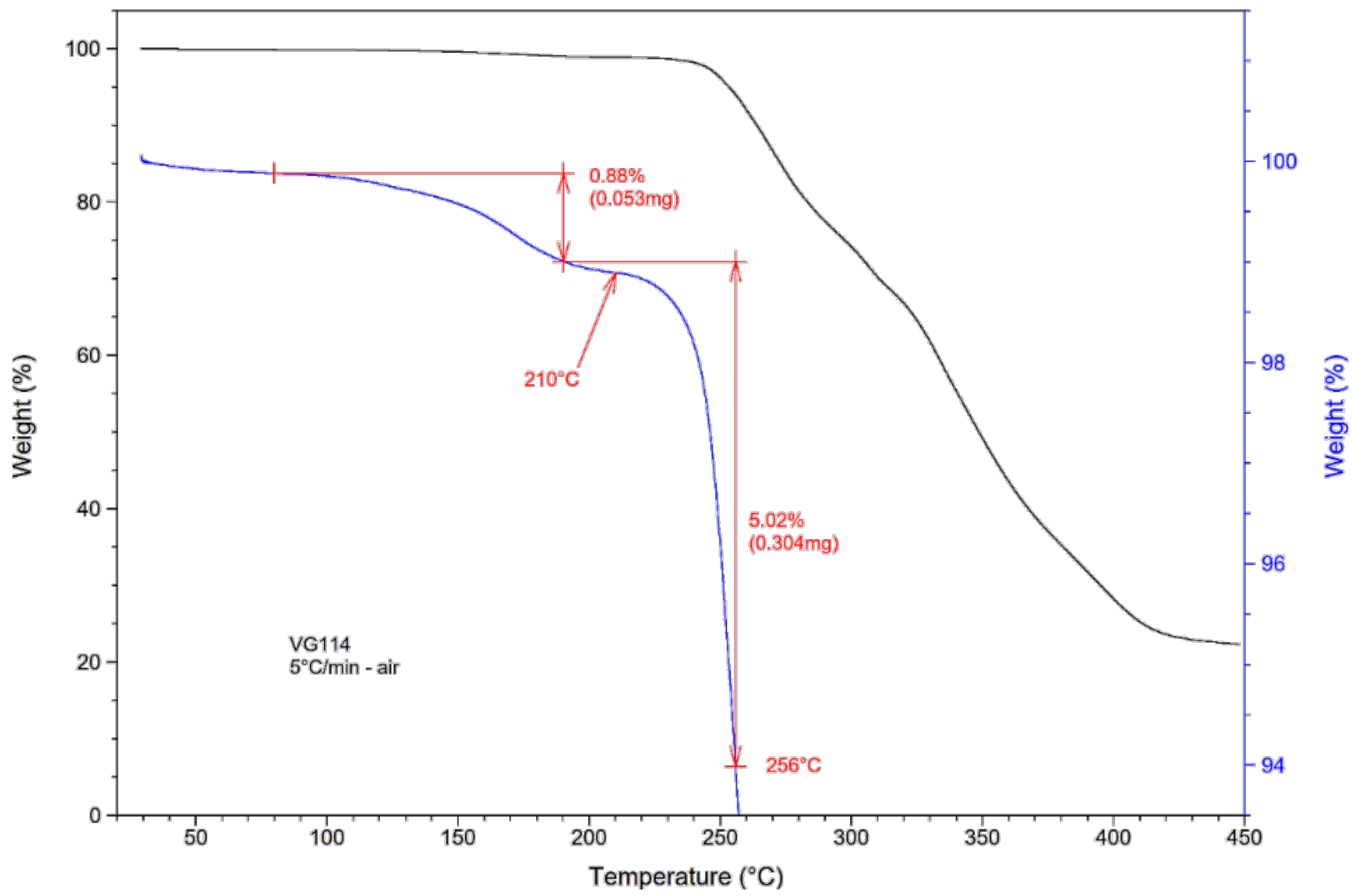

Figure S22. TGA curve of *R*-**1**. The product contains a volatile amount released between ca. 120 and 180°C. Significant weight loss from starting degradation observed above ca. 210°C. 5% weight loss temperature at 5°C/min: $T_{5\%}$ = 256°C (± 3°C).

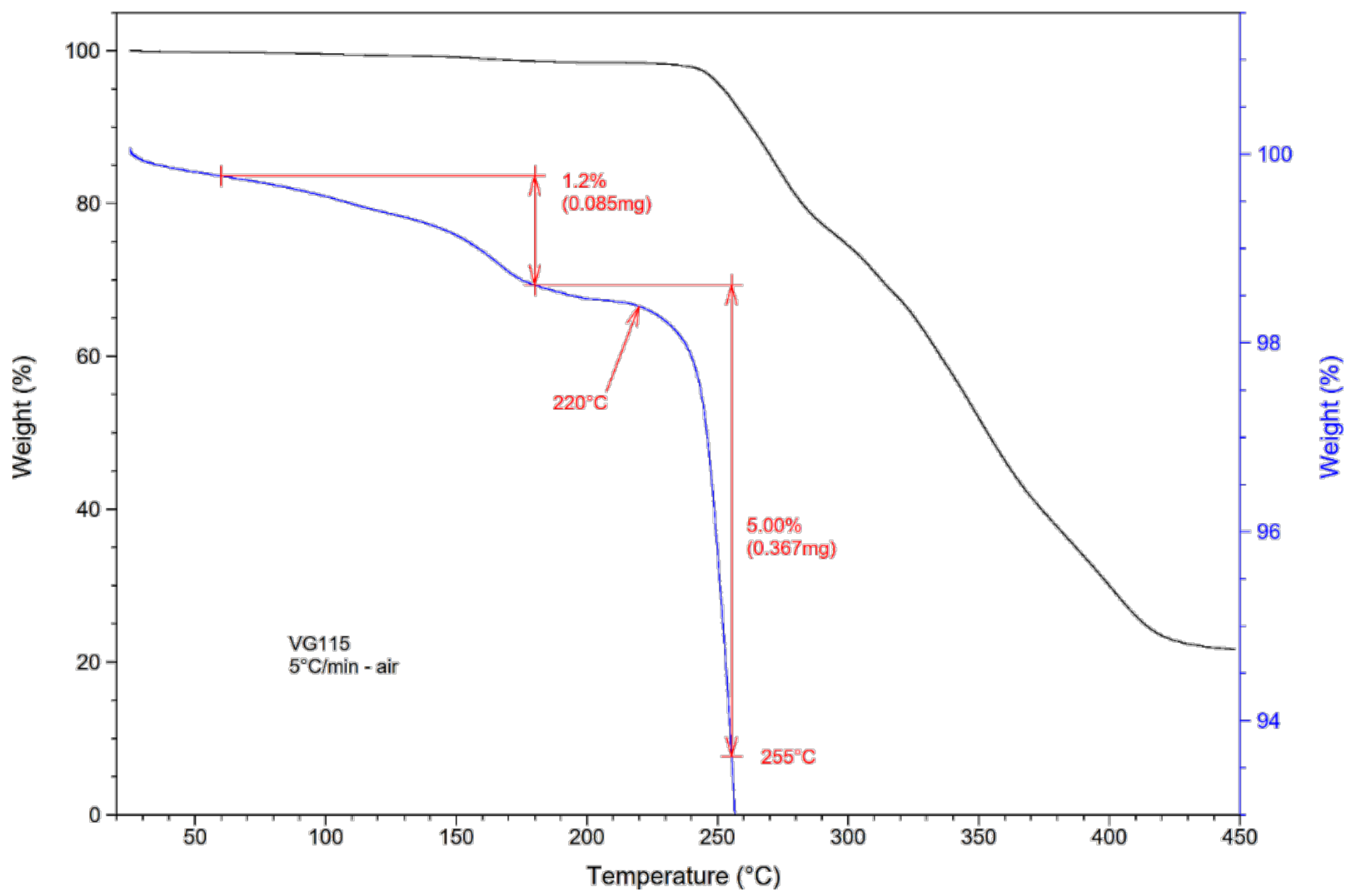

Figure S23. TGA curve of *S*-**1**. The product contains a small volatile amount released below 180°C. Significant weight loss from starting degradation observed above ca. 220°C. 5% weight loss temperature at 5°C/min: $T_{5\%}$ = 255°C (± 3°C).

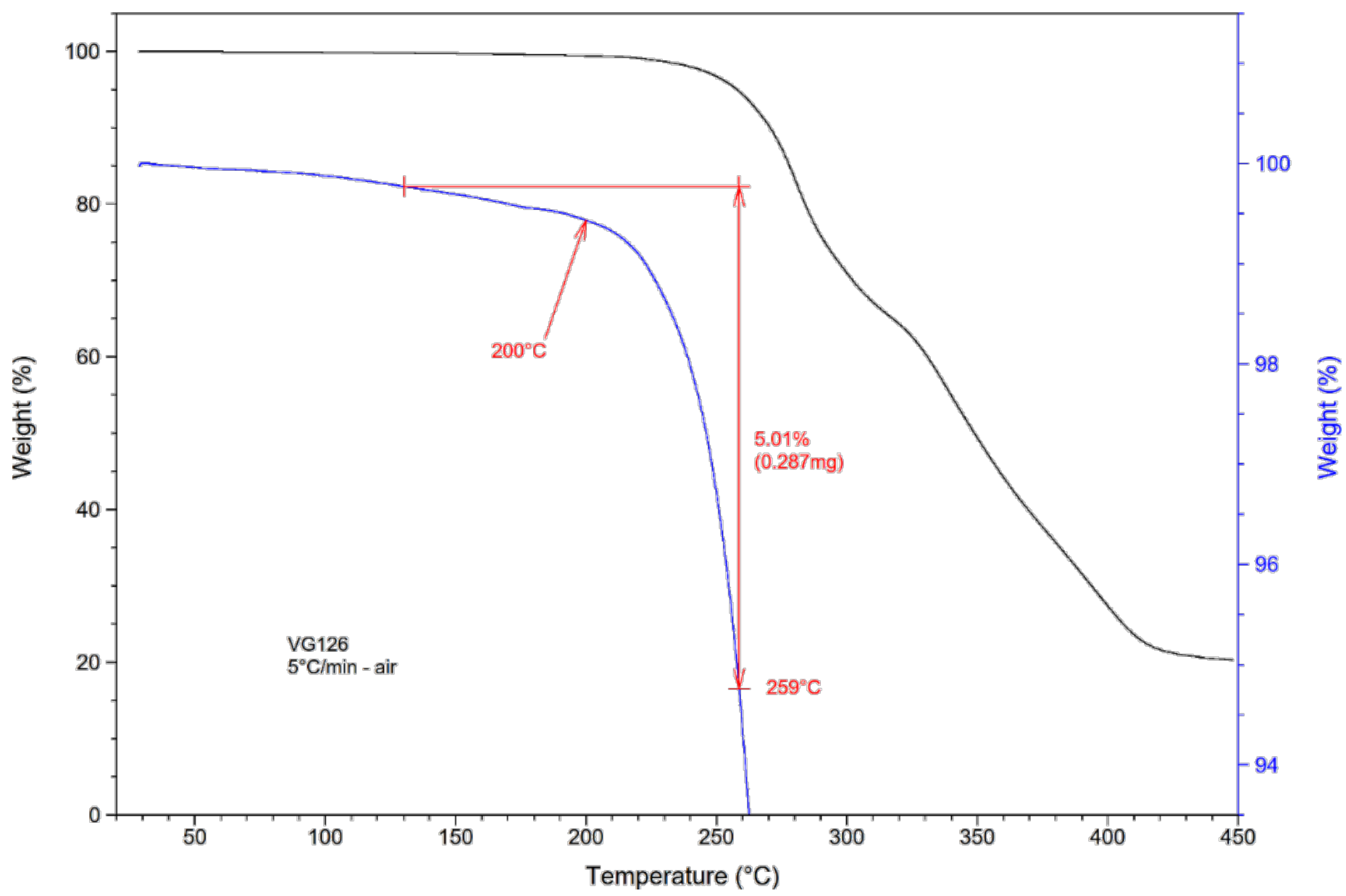

Figure S24. TGA curve of *R*-**2**. Significant weight loss from starting degradation observed above ca. 200°C. 5% weight loss temperature at 5°C/min: $T_{5\%}$ = 259°C (± 5°C).

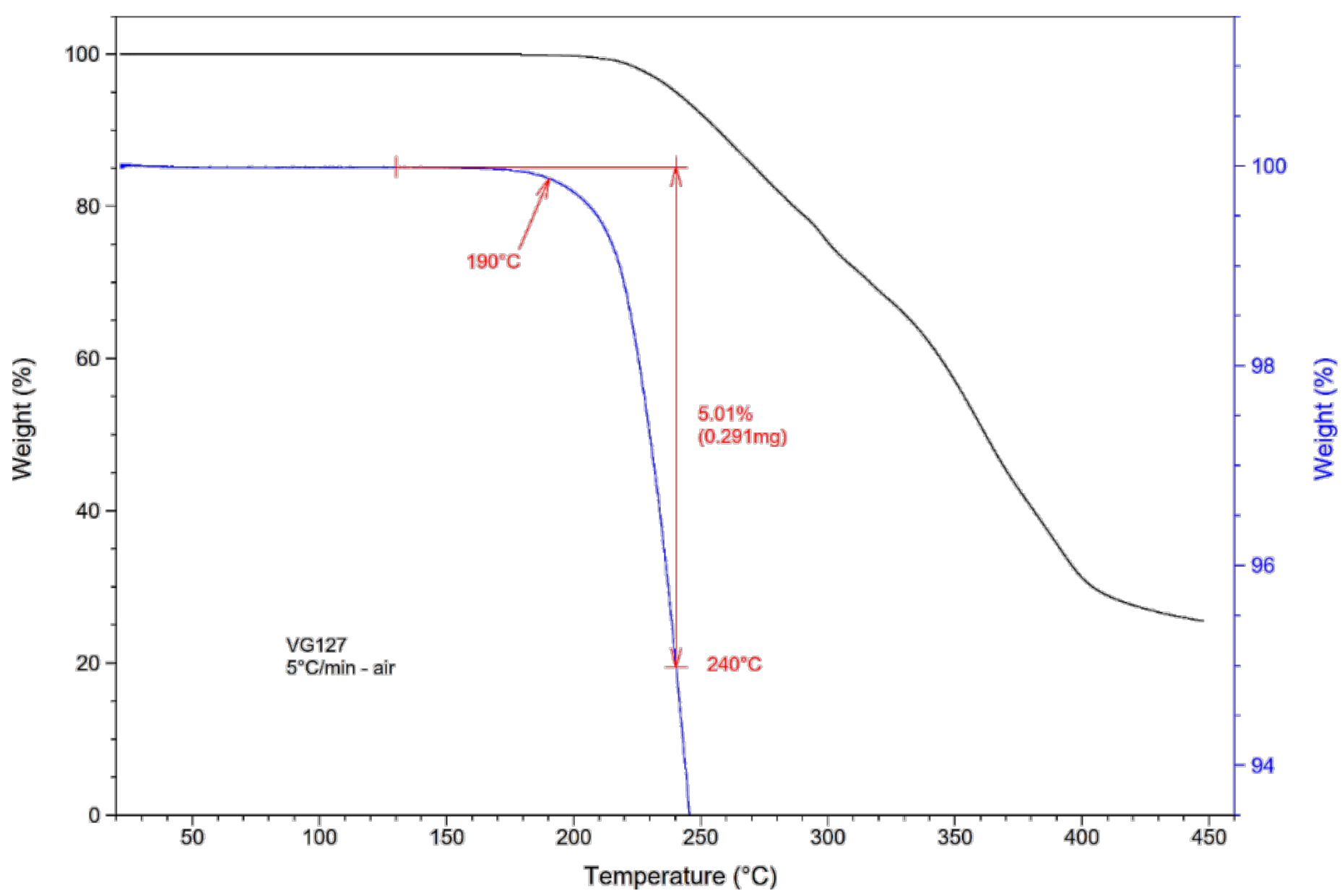

Figure S25. TGA curve of *S*-**2**. Significant weight loss from starting degradation observed above ca. 190°C. 5% weight loss temperature at 5°C/min: $T_{5\%}$ = 240°C (± 3°C).

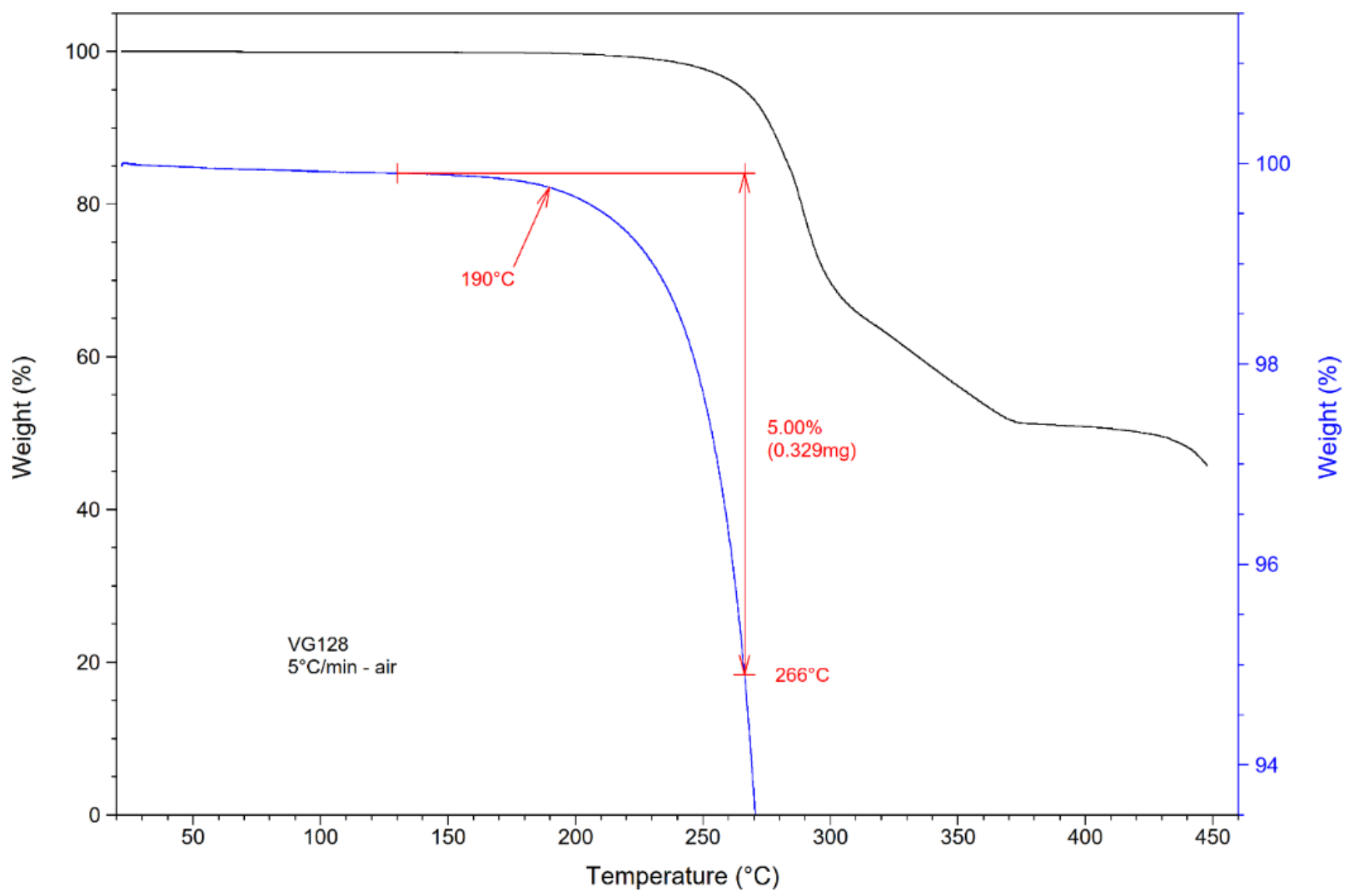

Figure S26. TGA curve of *R*-**3**. A sample measured after a preliminary drying at 180°C under nitrogen flux displayed significant weight loss from starting degradation above ca. 190°C. 5% weight loss temperature at 5°C/min: $T_{5\%}$ = 266°C (± 3°C).

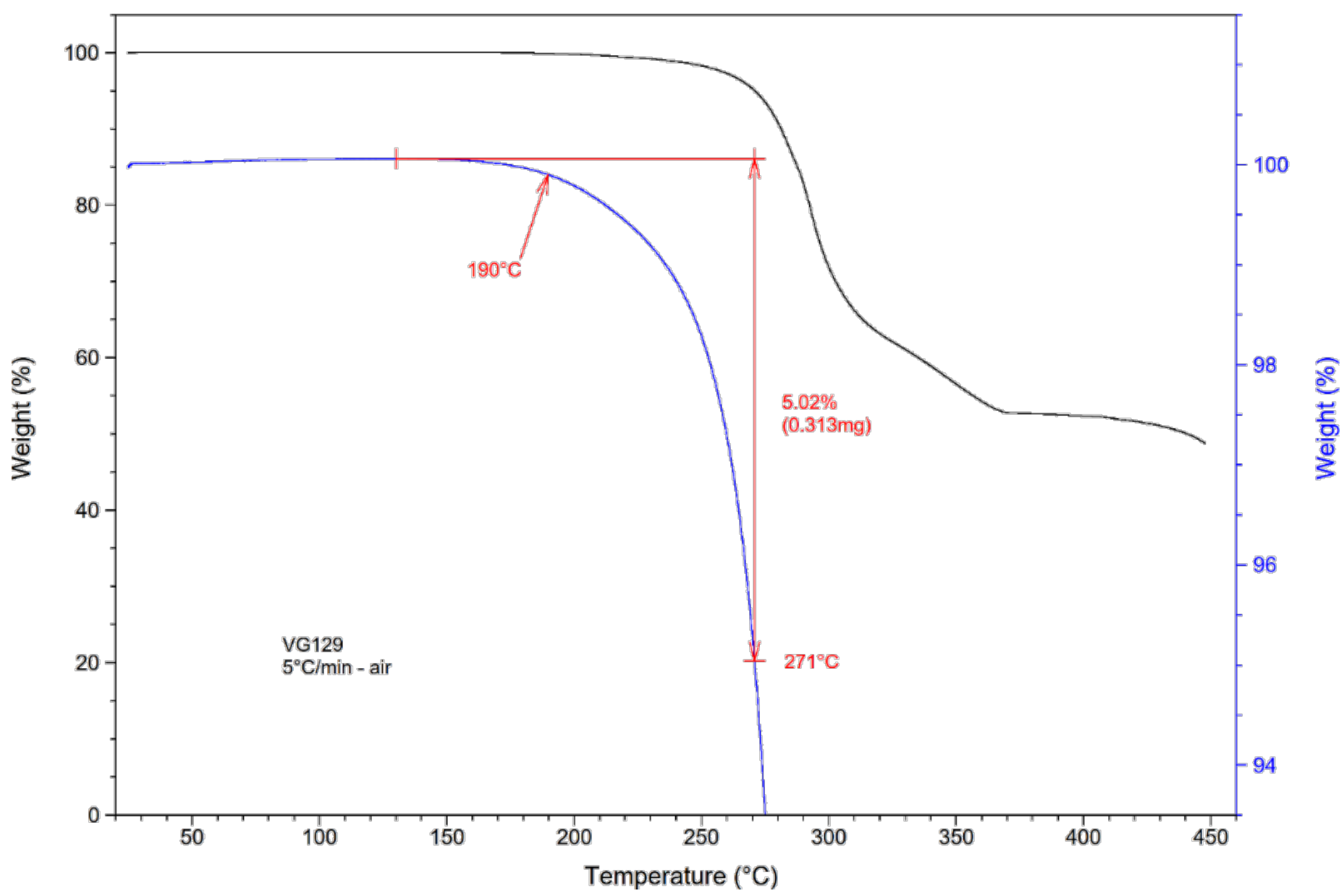

Figure S27. TGA curve of *S*-**3**. A sample measured after a preliminary drying at 180°C under nitrogen flux displayed significant weight loss from starting degradation above ca. 190°C. 5% weight loss temperature at 5°C/min: $T_{5\%}$ = 271°C (± 3°C).

Computational studies

All calculation were made using the ADF-2019 code[5] at the density functional theory with B3LYP functional (DFT).[6] All atoms were described with the TZP basis-set.[7] Scalar relativistic were introduced through the zero-order relativistic approximation (ZORA) Hamiltonian.[8] All calculations were made with a non-explicit dichloromethane solvent within the Conductor-like-screening model (COSMO)[9]. Weak interactions were introduced through Grimme D3 corrections.[10] The structures of all complexes were fully optimized and the absorption spectra computed through time dependent DFT (TD-DFT) using the Tamm-Dancoff approximation (TDA).[11] Spin-orbit coupling corrections were introduced by perturbation of the computed spectra. ECD spectra were computed in the same conditions but without Tamm-Dancoff corrections. The nature of the excited states has been analyzed with TheoDORE software.[12] Emission properties were determined after the optimization of triplet states geometries using unrestricted wavefunction approach, in the same conditions. Electron density difference and spin density maps were computed using the dgrid package and visualized using ChimeraX software[13] starting from ADF wavefunction.

Table S8. Absorption energies (E_{abs} in eV), absorption wavelength (λ_{abs} in nm) and oscillator strength (f_{osc}) of the computed singlet states of *R*-**1**, *R*-**2** and *R*-**3** within the TDA approximation.

	R-**1**			*R*-**2**			*R*-**3**		
State	E_{abs}	λ_{abs}	f_{osc}	E_{abs}	λ_{abs}	f_{osc}	E_{abs}	λ_{abs}	f_{osc}
S1	2.768	448	6.55E-02	2.646	469	1.23E-01	2.553	486	1.27E-01
S2	3.264	380	6.00E-04	3.045	407	7.74E-04	2.957	419	3.97E-04
S3	3.389	366	2.41E-03	3.198	388	1.05E-02	3.124	397	8.65E-03
S4	3.438	361	1.19E-01	3.476	357	9.64E-02	3.404	364	3.92E-02
S5	3.577	347	3.01E-02	3.515	353	1.11E+00	3.424	362	2.05E-02
S6	3.719	333	5.64E-02	3.589	345	1.57E-02	3.463	358	1.02E-01
S7	3.790	327	5.40E-01	3.602	344	1.37E-02	3.497	355	3.16E-01
S8	3.805	326	1.76E-02	3.627	342	2.63E-03	3.529	351	1.27E-02
S9	3.843	323	1.58E-02	3.700	335	1.79E-02	3.550	349	2.82E-01
S10	3.865	321	2.04E-01	3.744	331	7.90E-02	3.575	347	3.59E-01
S11	3.886	319	1.40E-01	3.750	331	4.27E-02	3.601	344	3.65E-03
S12	3.942	315	2.54E-02	3.803	326	1.05E-03	3.607	344	1.24E-02
S13	3.986	311	6.23E-03	3.834	323	7.98E-02	3.625	342	4.23E-02
S14	4.002	310	1.31E-01	3.852	322	3.65E-04	3.640	341	1.71E-02
S15	4.041	307	5.38E-02	3.878	320	2.78E-02	3.705	335	3.51E-03
S16	4.054	306	1.28E-01	3.950	314	7.04E-03	3.750	331	3.43E-02
S17	4.091	303	4.93E-03	3.983	311	1.10E-03	3.797	327	8.09E-02
S18	4.117	301	4.76E-02	3.997	310	3.40E-03	3.843	323	8.64E-02
S19	4.134	300	5.97E-03	4.032	307	2.01E-02	3.871	320	1.19E-02
S20	4.143	299	1.42E-02	4.085	304	4.67E-03	3.942	315	1.11E-02
S21	4.204	295	5.00E-02	4.113	301	9.48E-02	3.955	313	3.85E-03
S22	4.215	294	2.81E-02	4.134	300	8.82E-03	3.960	313	3.75E-03
S23	4.283	289	6.29E-02	4.137	300	2.34E-03	4.049	306	6.70E-03
S24	4.329	286	1.43E-03	4.156	298	2.57E-03	4.058	306	9.94E-03
S25	4.336	286	1.06E-02	4.178	297	3.85E-02	4.077	304	2.43E-03
S26	4.343	285	2.03E-02	4.179	297	1.87E-02	4.098	303	4.56E-03
S27	4.346	285	6.29E-02	4.238	293	6.91E-03	4.143	299	6.52E-02
S28	4.354	285	8.43E-02	4.286	289	5.83E-04	4.206	295	4.29E-03
S29	4.379	283	3.27E-02	4.293	289	1.28E-03	4.215	294	8.47E-03

S30	4.392	282	3.83E-02	4.307	288	1.10E-02	4.237	293	1.75E-02
S31	4.426	280	9.58E-02	4.327	287	1.75E-01	4.267	291	2.49E-02
S32	4.446	279	5.24E-02	4.360	284	1.02E-01	4.319	287	1.33E-02
S33	4.450	279	1.22E-02	4.411	281	1.14E-02	4.335	286	3.03E-01
S34	4.464	278	2.55E-02	4.434	280	1.26E-01	4.364	284	6.23E-03
S35	4.470	277	2.54E-02	4.444	279	1.40E-01	4.384	283	9.02E-03
S36	4.480	277	1.72E-01	4.451	279	1.77E-02	4.386	283	9.51E-03
S37	4.562	272	3.78E-02	4.478	277	5.78E-02	4.395	282	4.83E-02
S38	4.576	271	1.88E-02	4.488	276	2.20E-02	4.400	282	1.33E-01
S39	4.581	271	1.35E-02	4.492	276	6.93E-03	4.412	281	6.86E-02
S40	4.595	270	1.48E-02	4.538	273	1.00E-02	4.427	280	5.23E-02
S41	4.603	269	1.28E-03	4.544	273	5.85E-03	4.434	280	3.06E-02
S42	4.609	269	3.17E-02	4.558	272	7.74E-03	4.460	278	3.78E-02
S43	4.637	267	1.28E-02	4.559	272	3.00E-02	4.474	277	3.52E-03
S44	4.649	267	7.92E-02	4.577	271	7.09E-02	4.490	276	1.26E-02
S45	4.669	266	3.29E-02	4.597	270	5.17E-02	4.508	275	4.61E-02
S46	4.677	265	4.42E-02	4.602	269	7.44E-02	4.512	275	4.72E-02
S47	4.684	265	1.07E-01	4.612	269	9.40E-02	4.516	275	3.16E-02
S48	4.702	264	1.04E-04	4.626	268	6.47E-02	4.533	274	1.36E-02
S49	4.720	263	1.00E-02	4.629	268	1.23E-01	4.536	273	5.99E-03
S50	4.744	261	7.91E-02	4.660	266	6.28E-03	4.581	271	7.38E-02

Table S9. Absorption energies (E_{abs} in eV) and absorption wavelength (λ_{abs} in nm) of the computed triplet states of *R*-**1**, *R*-**2** and *R*-**3** within the TDA approximation.

	R-**1**			*R*-**2**			*R*-**3**		
State	E_{abs}	λ_{abs}		E_{abs}	λ_{abs}		E_{abs}	λ_{abs}	
T1	2.705	458		2.546	487		2.433	510	
T2	3.034	409		2.733	454		2.733	454	
T3	3.072	404		2.988	415		2.892	429	
T4	3.134	396		3.029	409		2.908	426	
T5	3.262	380		3.082	402		2.979	416	
T6	3.354	370		3.152	393		3.084	402	
T7	3.375	367		3.185	389		3.115	398	
T8	3.388	366		3.295	376		3.183	389	
T9	3.512	353		3.405	364		3.336	372	
T10	3.683	337		3.486	356		3.407	364	
T11	3.693	336		3.563	348		3.443	360	
T12	3.762	330		3.603	344		3.460	358	
T13	3.788	327		3.688	336		3.528	351	
T14	3.807	326		3.705	335		3.556	349	
T15	3.825	324		3.784	328		3.564	348	
T16	3.855	322		3.793	327		3.601	344	
T17	3.869	320		3.804	326		3.613	343	
T18	3.915	317		3.817	325		3.624	342	
T19	3.947	314		3.833	323		3.648	340	
T20	3.972	312		3.846	322		3.703	335	
T21	3.987	311		3.939	315		3.792	327	
T22	3.993	311		3.947	314		3.805	326	
T23	4.027	308		3.972	312		3.831	324	
T24	4.072	305		3.978	312		3.852	322	
T25	4.096	303		3.985	311		3.857	321	
T26	4.106	302		3.989	311		3.880	320	
T27	4.115	301		4.014	309		3.927	316	
T28	4.142	299		4.059	305		3.934	315	
T29	4.169	297		4.063	305		3.948	314	
T30	4.177	297		4.088	303		3.950	314	
T31	4.202	295		4.111	302		3.977	312	
T32	4.239	292		4.123	301		4.006	309	
T33	4.242	292		4.136	300		4.010	309	
T34	4.245	292		4.147	299		4.024	308	

T35	4.269	290		4.152	299		4.054	306	
T36	4.278	290		4.157	298		4.061	305	
T37	4.313	287		4.167	298		4.072	304	
T38	4.325	287		4.214	294		4.075	304	
T39	4.329	286		4.222	294		4.106	302	
T40	4.336	286		4.243	292		4.120	301	
T41	4.342	286		4.265	291		4.123	301	
T42	4.369	284		4.274	290		4.148	299	
T43	4.378	283		4.277	290		4.152	299	
T44	4.384	283		4.285	289		4.162	298	
T45	4.398	282		4.296	289		4.202	295	
T46	4.429	280		4.310	288		4.215	294	
T47	4.453	278		4.351	285		4.232	293	
T48	4.456	278		4.393	282		4.259	291	
T49	4.469	277		4.415	281		4.297	289	
T50	4.472	277		4.437	279		4.313	287	

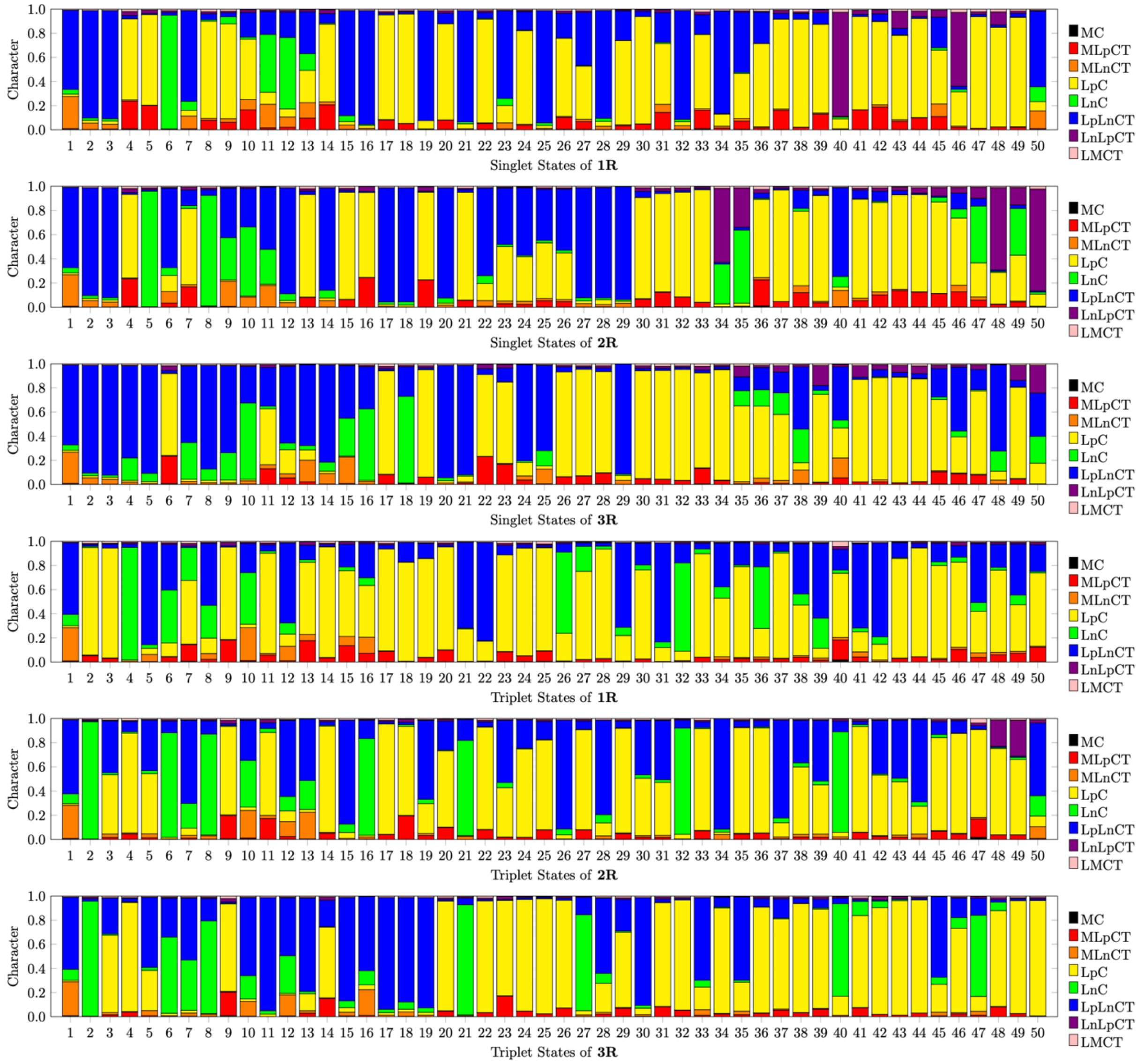

Figure S28. Theodore analysis of the singlet and triplet states of the three complexes computed at Franck-Condon geometry within the TDA approximation. Lp stands for the P^P ligand and Ln for the N^N ligand.

Table S10. Absorption energies (E_{abs} in eV), absorption wavelength (λ_{abs} in nm), oscillator strength (f_{osc}) and Rotatory strength (R) of the computed singlet states of *R*-**1** without the TDA approximation.

R-**1**				
State	E_{abs}	λ_{abs}	R	f_{osc}
S1	2.752	451	17.2	6.08E-02
S2	3.264	380	2.0	7.58E-04
S3	3.387	366	-0.6	1.51E-03
S4	**3.425**	**362**	**136.5**	**1.20E-01**
S5	3.537	351	-53.1	3.10E-02
S6	3.684	337	-40.6	4.61E-01
S7	3.733	332	-27.5	2.26E-01
S8	3.777	328	197.4	9.39E-02
S9	3.788	327	-161.4	5.21E-02
S10	3.838	323	43.3	5.30E-02
S11	3.856	322	-92.3	3.17E-02
S12	3.917	316	30.8	2.81E-02
S13	3.927	316	-64.3	9.29E-02
S14	3.970	312	-0.2	1.00E-03
S15	3.995	310	59.1	1.51E-01
S16	4.021	308	28.8	2.25E-02
S17	4.041	307	7.0	6.48E-02
S18	4.085	303	3.1	2.25E-03
S19	4.111	302	-42.0	2.78E-02
S20	4.130	300	-0.9	2.46E-03
S21	4.168	297	11.1	5.16E-02
S22	4.210	294	-3.6	1.55E-02
S23	4.267	291	22.6	7.42E-02
S24	4.311	288	-49.1	6.31E-02
S25	4.316	287	18.5	8.90E-02
S26	4.324	287	-4.4	4.95E-03
S27	4.327	287	-15.6	1.26E-02
S28	4.337	286	-23.0	1.70E-02
S29	4.354	285	-4.1	4.61E-02
S30	4.384	283	-17.7	1.89E-02
S31	4.407	281	-207.9	1.47E-01
S32	4.423	280	4.1	8.55E-03
S33	4.438	279	-64.8	7.09E-02
S34	4.445	279	232.6	2.20E-01
S35	4.464	278	1.7	1.23E-04
S36	4.469	277	-8.1	1.09E-02
S37	4.536	273	6.0	3.81E-02
S38	4.563	272	36.3	2.37E-02
S39	4.573	271	9.4	1.17E-02
S40	4.589	270	23.8	2.56E-02
S41	4.594	270	-41.8	2.78E-02
S42	4.602	269	-1.0	1.78E-03
S43	4.617	269	73.9	8.20E-02
S44	4.628	268	-12.0	3.64E-02
S45	4.650	267	-163.4	6.17E-02
S46	4.658	266	103.6	1.07E-01
S47	4.672	265	77.6	5.05E-02
S48	4.698	264	-13.5	2.52E-03
S49	4.709	263	-25.3	5.28E-03
S50	4.724	262	-2.6	1.40E-01

Table S11. Absorption energies (E_{abs} in eV), absorption wavelength (λ_{abs} in nm), oscillator strength (f_{osc}) and Rotatory strength (R) of the computed singlet states of *R*-**2** without the TDA approximation.

R-**2**				
State	E_{abs}	λ_{abs}	R	f_{osc}
S1	2.616	474	38.8	1.18E-01
S2	3.045	407	0.4	7.03E-04
S3	3.193	388	1.4	9.05E-03
S4	3.425	362	-34.8	9.53E-01
S5	3.453	359	167.7	8.35E-02
S6	3.562	348	5.7	4.63E-02
S7	3.579	346	-45.1	1.18E-02
S8	3.595	345	-62.1	1.74E-02
S9	3.662	339	-10.5	5.89E-02
S10	3.691	336	-2.3	6.76E-02
S11	3.743	331	7.8	1.37E-02
S12	3.763	329	-98.0	9.48E-02
S13	3.798	326	-1.1	4.89E-04
S14	3.846	322	0.3	2.21E-05
S15	3.865	321	19.1	1.82E-02
S16	3.919	316	0.3	7.72E-03
S17	3.981	311	1.6	8.48E-04
S18	3.991	311	-1.6	1.57E-02
S19	3.994	310	-0.4	2.88E-03
S20	4.029	308	32.1	9.23E-03
S21	4.055	306	-12.4	1.21E-01
S22	4.080	304	-1.0	1.53E-03
S23	4.122	301	-2.8	4.20E-03
S24	4.144	299	1.9	4.29E-04
S25	4.159	298	5.7	4.14E-02
S26	4.173	297	1.5	1.92E-03
S27	4.233	293	-1.2	7.39E-03
S28	4.247	292	4.6	1.04E-02
S29	4.283	289	-0.1	1.53E-03
S30	4.291	289	0.2	2.50E-03
S31	4.298	288	-220.9	2.27E-01
S32	4.322	287	-45.8	4.17E-02
S33	4.357	285	-1.9	2.33E-01
S34	4.370	284	-7.5	3.52E-03
S35	4.431	280	-4.5	1.17E-03
S36	4.442	279	23.1	2.15E-02
S37	4.457	278	-40.9	5.97E-02
S38	4.482	277	-1.0	4.60E-03
S39	4.492	276	31.2	2.42E-02
S40	4.505	275	8.3	6.02E-03
S41	4.532	274	18.6	4.23E-03
S42	4.539	273	-27.8	2.11E-02
S43	4.545	273	-8.9	2.85E-02
S44	4.561	272	-65.1	1.84E-01
S45	4.563	272	65.3	9.12E-02
S46	4.587	270	80.2	1.11E-01
S47	4.594	270	68.7	4.21E-02
S48	4.595	270	94.1	1.13E-01
S49	4.627	268	11.9	2.33E-02
S50	4.646	267	-6.5	2.14E-03

Table S12. Absorption energies (E_{abs} in eV), absorption wavelength (λ_{abs} in nm), oscillator strength (f_{osc}) and Rotatory strength (R) of the computed singlet states of *R*-**3** without the TDA approximation.

R-**3**				
State	E_{abs}	λ_{abs}	R	f_{osc}
S1	2.515	493	33.3	1.13E-01
S2	2.957	419	-0.1	3.82E-04
S3	3.118	398	1.5	6.77E-03
S4	3.372	368	40.2	2.14E-01
S5	3.415	363	-10.2	4.52E-02
S6	3.417	363	186.7	8.22E-02
S7	3.454	359	-29.6	4.37E-01
S8	3.518	352	6.8	9.02E-02
S9	3.529	351	-50.1	1.85E-01
S10	3.547	350	-3.8	1.29E-02
S11	3.584	346	-58.5	1.43E-02
S12	3.589	345	-3.0	2.97E-03
S13	3.621	342	7.1	1.42E-02
S14	3.627	342	-16.5	1.06E-02
S15	3.667	338	-12.3	6.50E-03
S16	3.693	336	-1.3	3.45E-03
S17	3.741	331	-117.7	8.73E-02
S18	3.786	327	10.5	7.42E-02
S19	3.860	321	25.6	1.75E-02
S20	3.931	315	11.1	4.78E-03
S21	3.939	315	1.0	5.47E-03
S22	3.953	314	0.5	1.70E-03
S23	3.984	311	-9.9	3.95E-03
S24	4.046	306	22.2	1.45E-02
S25	4.052	306	2.9	4.39E-03
S26	4.061	305	-1.9	4.64E-04
S27	4.068	305	6.5	8.20E-02
S28	4.155	298	21.6	2.19E-02
S29	4.205	295	-1.2	2.35E-03
S30	4.212	294	28.6	1.03E-02
S31	4.243	292	-35.0	1.51E-02
S32	4.276	290	-11.5	1.56E-02
S33	4.288	289	-301.3	2.76E-01
S34	4.333	286	-39.7	2.50E-01
S35	4.356	285	-7.9	3.56E-03
S36	4.376	283	4.2	8.06E-03
S37	4.381	283	21.7	9.42E-03
S38	4.386	283	-22.8	2.88E-02
S39	4.401	282	-1.2	2.34E-02
S40	4.416	281	-42.6	1.66E-02
S41	4.425	280	55.4	2.67E-02
S42	4.443	279	9.3	2.76E-02
S43	4.450	279	-8.1	2.23E-02
S44	4.486	276	-5.8	2.17E-02
S45	4.499	276	-119.1	5.09E-02
S46	4.505	275	5.5	3.38E-02
S47	4.506	275	4.8	2.53E-02
S48	4.516	275	13.3	1.79E-02
S49	4.520	274	-26.0	2.84E-02
S50	4.559	272	46.7	1.93E-01

References

(1) Crosby, G. A.; Demas, J. N. Quantum Efficiencies of Transition-Metal Complexes. I. d-d Luminescence. *J. Am. Chem. Soc.* **1970**, *92* (25), 7262–7270. https://doi.org/10.1021/ja00728a006.

(2) Ishida, H.; Tobita, S.; Hasegawa, Y.; Katoh, R.; Nozaki, K. Recent Advances in Instrumentation for Absolute Emission Quantum Yield Measurements. *Coord. Chem. Rev.* **2010**, *254* (21), 2449–2458. https://doi.org/10.1016/j.ccr.2010.04.006.

(3) Lakowicz, J. R. *Principles of Fluorescence Spectroscopy*, Third edition, corrected at 4. printing.; Springer: New York, NY, 2010.

(4) Zinna, F.; Bruhn, T.; Guido, C. A.; Ahrens, J.; Bröring, M.; Di Bari, L.; Pescitelli, G. Circularly Polarized Luminescence from Axially Chiral BODIPY DYEmers: An Experimental and Computational Study. *Chem. – Eur. J.* **2016**, *22* (45), 16089–16098. https://doi.org/10.1002/chem.201602684.

(5) Vrije Universiteit, Amsterdam. *ADF Manual 2019.1.* SCM. http://www.scm.com (accessed 2025-09-26).

(6) Stephens, P. J.; Devlin, F. J.; Chabalowski, C. F.; Frisch, M. J. Ab Initio Calculation of Vibrational Absorption and Circular Dichroism Spectra Using Density Functional Force Fields. *J. Phys. Chem.* **1994**, *98* (45), 11623–11627. https://doi.org/10.1021/j100096a001.

(7) Van Lenthe, E.; Baerends, E. J. Optimized Slater-Type Basis Sets for the Elements 1–118. *J. Comput. Chem.* **2003**, *24* (9), 1142–1156. https://doi.org/10.1002/jcc.10255.

(8) van Lenthe, E.; Ehlers, A.; Baerends, E.-J. Geometry Optimizations in the Zero Order Regular Approximation for Relativistic Effects. *J. Chem. Phys.* **1999**, *110* (18), 8943–8953. https://doi.org/10.1063/1.478813.

(9) Klamt, A.; Schüürmann, G. COSMO: A New Approach to Dielectric Screening in Solvents with Explicit Expressions for the Screening Energy and Its Gradient. *J. Chem. Soc., Perkin Trans. 2* **1993**, No. 5, 799–805. https://doi.org/10.1039/P29930000799.

(10) Grimme, S.; Antony, J.; Ehrlich, S.; Krieg, H. A Consistent and Accurate *Ab Initio* Parametrization of Density Functional Dispersion Correction (DFT-D) for the 94 Elements H-Pu. *J. Chem. Phys.* **2010**, *132* (15), 154104. https://doi.org/10.1063/1.3382344.

(11) Hirata, S.; Head-Gordon, M. Time-Dependent Density Functional Theory within the Tamm–Dancoff Approximation. *Chem. Phys. Lett.* **1999**, *314* (3), 291–299. https://doi.org/10.1016/S0009-2614(99)01149-5.

(12) Plasser, F. TheoDORE: A Toolbox for a Detailed and Automated Analysis of Electronic Excited State Computations. *J. Chem. Phys.* **2020**, *152* (8), 084108. https://doi.org/10.1063/1.5143076.

(13) Pettersen, E. F.; Goddard, T. D.; Huang, C. C.; Couch, G. S.; Greenblatt, D. M.; Meng, E. C.; Ferrin, T. E. UCSF Chimera—A Visualization System for Exploratory Research and Analysis. *J. Comput. Chem.* **2004**, *25* (13), 1605–1612. https://doi.org/10.1002/jcc.20084.
